# Supplementary material for: Maintaining and breaking symmetry in homomeric coiled-coil assemblies
Source: Nat Commun. 2018 Oct 8;9:4132. doi: 10.1038/s41467-018-06391-y (PMC6175849; doi:10.1038/s41467-018-06391-y)
Supplement: Supplementary file 1 — Supplementary Information [file 41467_2018_6391_MOESM1_ESM.pdf]

Maintaining and breaking symmetry in homomeric coiled-coil assemblies

Rhys *et al.*

Sequence List

gabcde	**Sequence**
β at ***a*** and ***d***	*cdefgab cdefgab cdefgab cdefgab*
CC-Type2-IV	Ac-G EVAQAIK EVAKAIK EVAWAIK EVAQAIK G-NH$_2$
CC-Type2-II	Ac-G EIAQAIK EIAKAIK EIAWAIK EIAQAIK G-NH$_2$
CC-Type2-VI	Ac-G EIAQAVK EIAKAVK EIAWAVK EIAQAVK G-NH$_2$
CC-Type2-VV	Ac-G EVAQAVK EVAKAVK EVAWAVK EVAQAVK G-NH$_2$
β only at ***d***	
CC-Type2-LI	Ac-G EIAQALK EIAKALK EIAWALK EIAQALK G-NH$_2$
CC-Type2-deLI	Ac-G EIAQAXK EIAKAXK EIAWAXK EIAQAXK G-NH$_2$
CC-Type2-LV	Ac-G EVAQALK EVAKALK EVAWALK EVAQALK G-NH$_2$
β only at ***a***	
CC-Type2-VL	Ac-G ELAQAVK ELAKAVK ELAWAVK ELAQAVK G-NH$_2$
CC-Type2-IL-Sg	Ac-G ELAQSIK ELAKSIK ELAWSIK ELAQSIK G-NH$_2$
CC-Type2-IL-Sg-L17E	Ac-G ELAQSIK ELAKSIK EEAWSIK ELAQSIK G-NH$_2$
	gabcdef gabcdef gabcdef gabcdef
CC-Type2-IL-Eg	Ac-G EIKALAQ EIKALAK EIKALAW EIKALAQ G-NH$_2$
No β residues	*cdefgab cdefgab cdefgab cdefgab*
CC-Type2-LL	Ac-G ELAQALK ELAKALK ELAWALK ELAQALK G-NH$_2$
CC-Type2-LL-Sg	Ac-G ELAQSLK ELAKSLK ELAWSLK ELAQSLK G-NH$_2$
CC-Type2-LL-L17Q	Ac-G ELAQALK ELAKALK EQAWALK ELAQALK G-NH$_2$
CC-Type2-LL-L17E	Ac-G ELAQALK ELAKALK EEAWALK ELAQALK G-NH$_2$
5H2L_2.1-I9L	Ac- TQEYLLK ELMKLLK EQIKLLK EQIKMLK ELEKQ-NH$_2$
Phenylalanine Sequences	
CC-Type2-IF	Ac-G EFAQAIK EFAKAIK EFAWAIK EFAQAIK G-NH$_2$
CC-Type2-FV	Ac-G EVAQAFK EVAKAFK EVAWAFK EVAQAFK G-NH$_2$
CC-Type2-FI	Ac-G EIAQAFK EIAKAFK EIAWAFK EIAQAFK G-NH$_2$
CC-Type2-LF	Ac-G EFAQALK EFAKALK EFAWALK EFAQALK G-NH$_2$
CC-Type2-FL	Ac-G ELAQAFK ELAKAFK ELAWAFK ELAQAFK G-NH$_2$
CC-Type2-FF	Ac-G EFAQAFK EFAKAFK EFAWAFK EFAQAFK G-NH$_2$

Supplementary Table 1 Peptide sequences discussed in this chapter written with their corresponding six-letter repeat name. X = 4,5-dehydroleucine or three-letter code deL.

Matrix-assisted laser desorption/ionisation - time of flight (MALDI-TOF) and Analytical high-pressure liquid chromatography (HPLC)

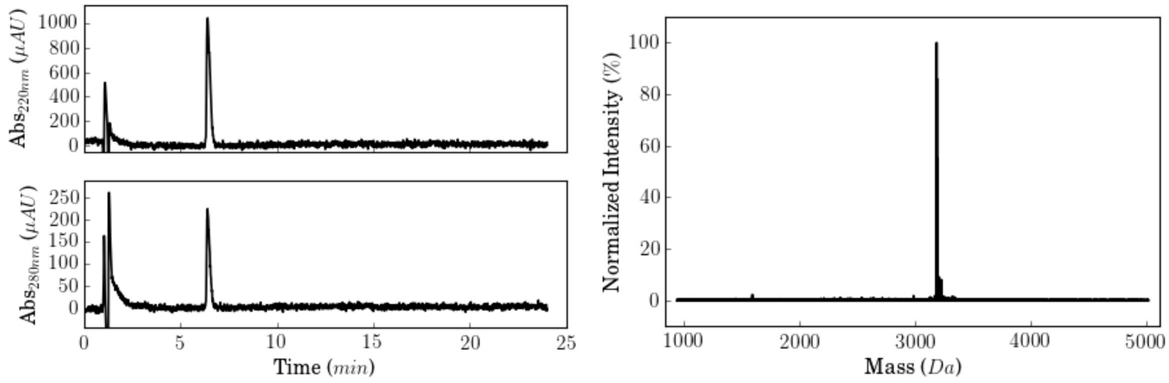

Supplementary Figure 1 CC-Type2-IV - HPLC traces from a gradient of 40 to 100% MeCN (0.1% TFA) in H_2O (0.1% TFA) (left, 220 and 280 nm) and MALDI-TOF MS (right). Calculated mass = 3191.7 Da, observed mass = 3191 Da.

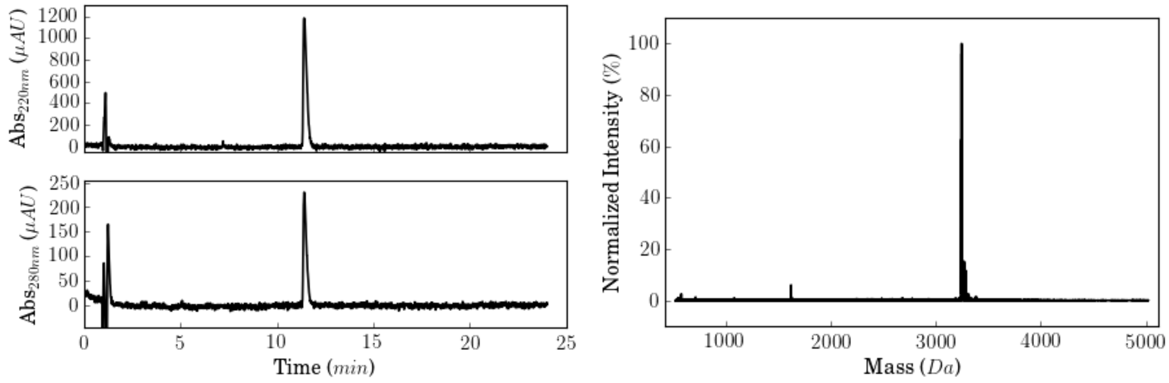

Supplementary Figure 2 CC-Type2-II - HPLC traces from a gradient of 40 to 100% MeCN (0.1% TFA) in H_2O (0.1% TFA) (left, 220 and 280 nm) and MALDI-TOF MS (right). Calculated mass = 3245.9 Da, observed mass = 3246 Da.

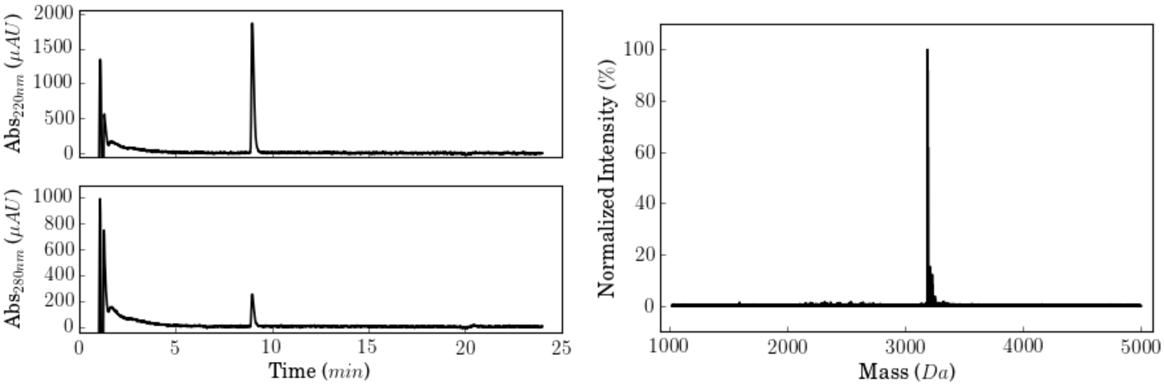

Supplementary Figure 3 CC-Type2-VI - HPLC traces from a gradient of 40 to 100% MeCN (0.1% TFA) in H_2O (0.1% TFA) (left, 220 and 280 nm) and MALDI-TOF MS (right). Calculated mass = 3189.8 Da, observed mass = 3191 Da.

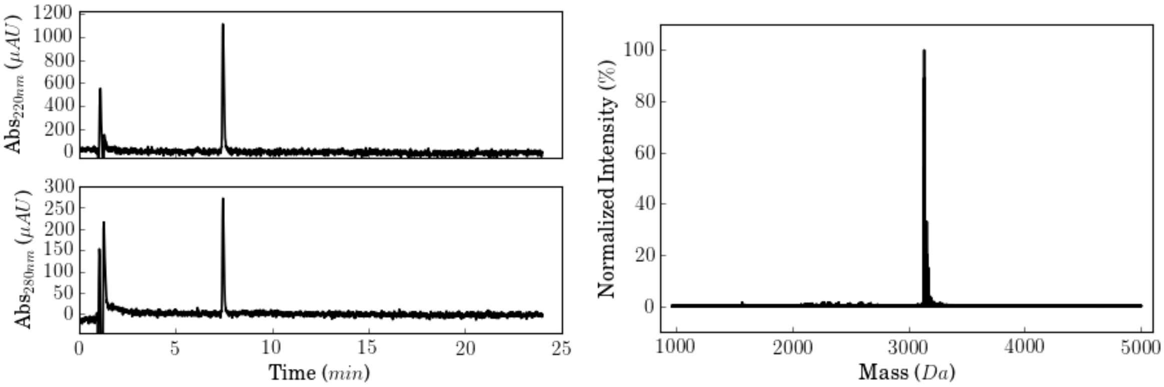

Supplementary Figure 4 CC-Type2-VV - HPLC traces from a gradient of 40 to 100% MeCN (0.1% TFA) in H_2O (0.1% TFA) (left, 220 and 280 nm) and MALDI-TOF MS (right). Calculated mass = 3133.8 Da, observed mass = 3134 Da.

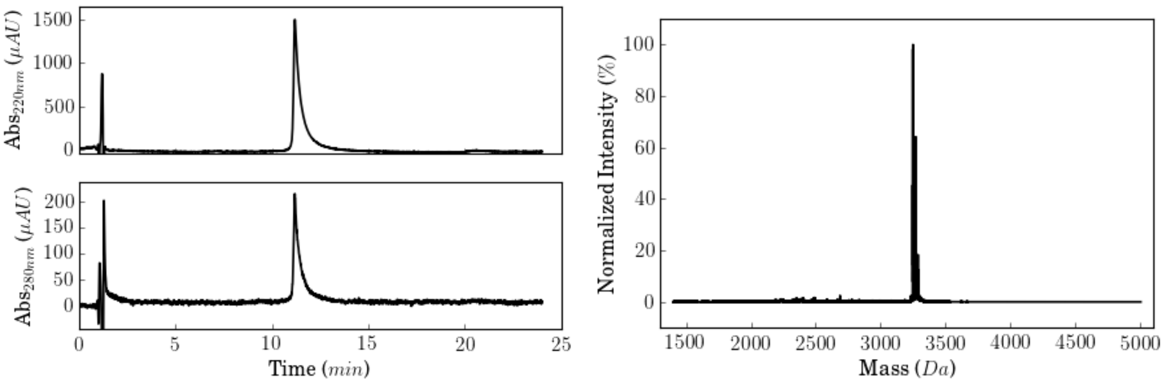

Supplementary Figure 5 CC-Type2-LI - HPLC traces from a gradient of 40 to 100% MeCN (0.1% TFA) in H_2O (0.1% TFA) (left, 220 and 280 nm) and MALDI-TOF MS (right). Calculated mass = 3245.9 Da, observed mass = 3246 Da.

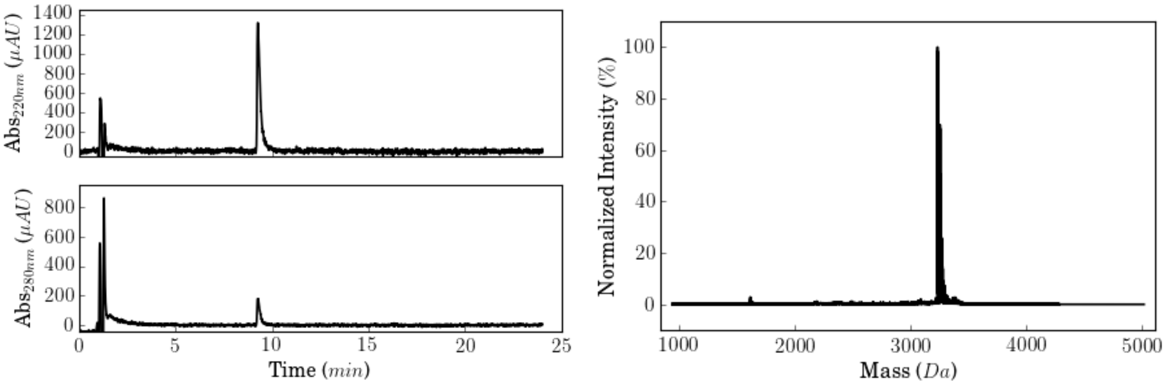

Supplementary Figure 6 CC-Type2-deLI - HPLC traces from a gradient of 40 to 100% MeCN (0.1% TFA) in H_2O (0.1% TFA) (left, 220 and 280 nm) and MALDI-TOF MS (right). Calculated mass = 3236.6 Da, observed mass = 3238 Da.

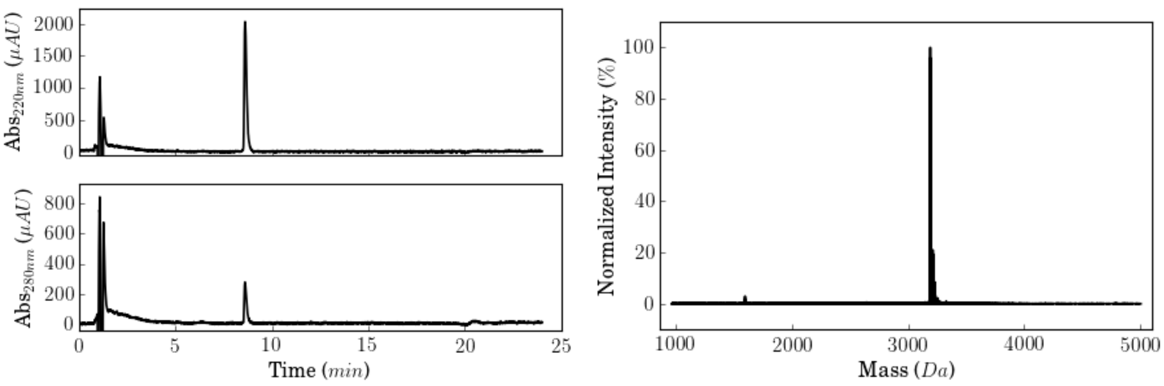

Supplementary Figure 7 CC-Type2-LV - HPLC traces from a gradient of 40 to 100% MeCN (0.1% TFA) in H_2O (0.1% TFA) (left, 220 and 280 nm) and MALDI-TOF MS (right). Calculated mass = 3189.8 Da, observed mass = 3190 Da.

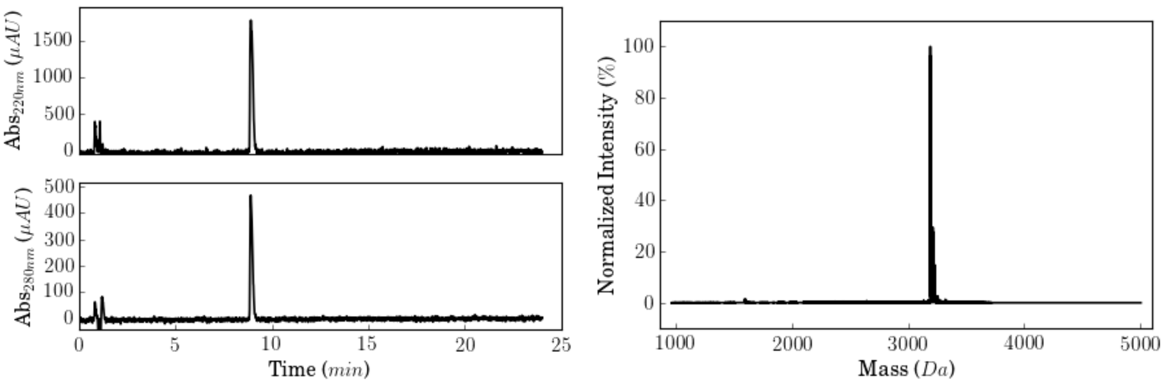

Supplementary Figure 8 CC-Type2-VL - HPLC traces from a gradient of 40 to 100% MeCN (0.1% TFA) in H_2O (0.1% TFA) (left, 220 and 280 nm) at 50 °C and MALDI-TOF MS (right). Calculated mass = 3189.8 Da, observed mass = 3190 Da.

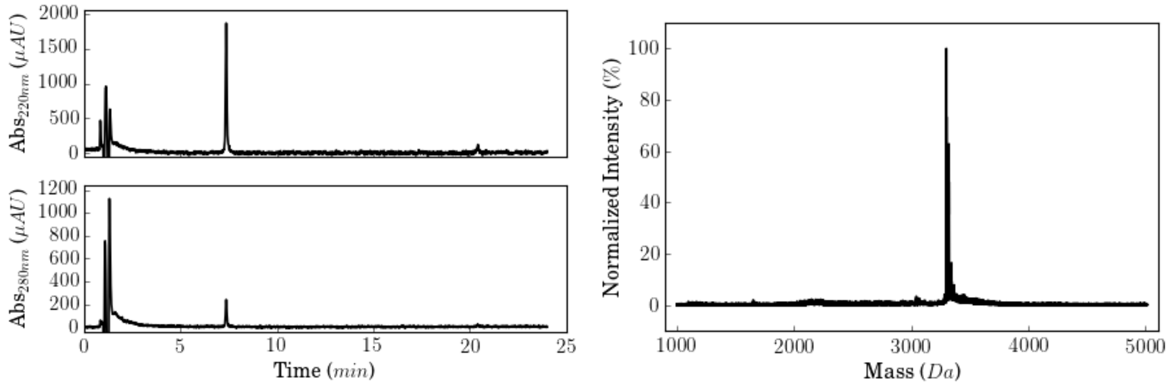

Supplementary Figure 9 CC-Type2-IL-Sg - HPLC traces from a gradient of 40 to 100% MeCN (0.1% TFA) in H_2O (0.1% TFA) (left, 220 and 280 nm) and MALDI-TOF MS (right). Calculated mass = 3309.9 Da, observed mass = 3300 Da.

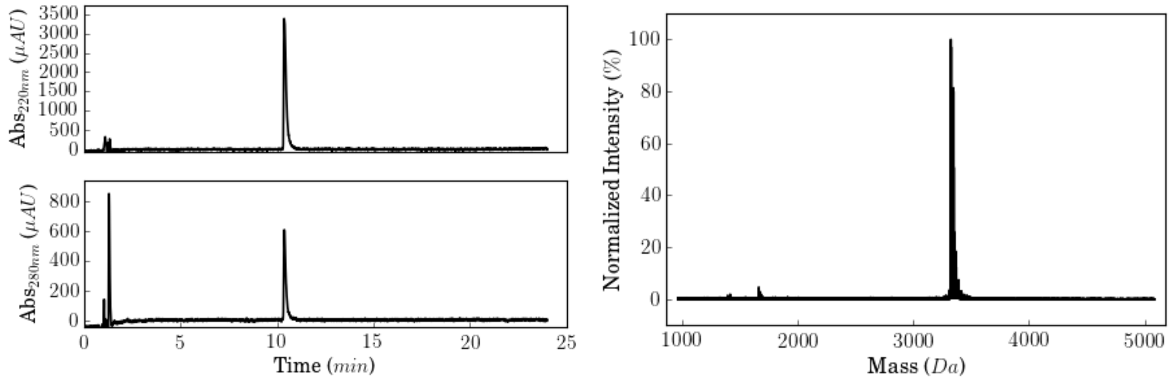

Supplementary Figure 10 CC-Type2-IL-Sg-L17E - HPLC traces from a gradient of 20 to 80 MeCN (0.1% TFA) in H_2O (0.1% TFA) (left, 220 and 280 nm) and MALDI-TOF MS (right). Calculated mass = 3325.8 Da, observed mass = 3325 Da.

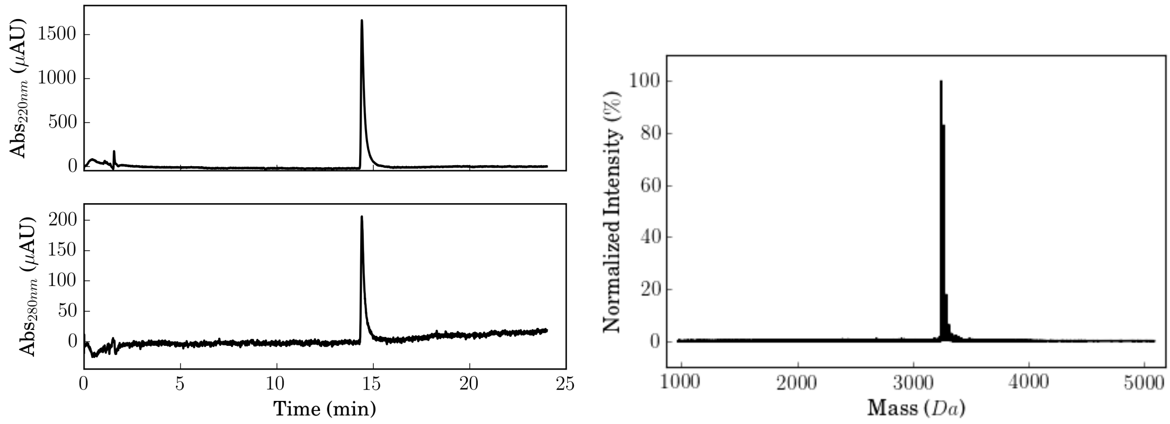

Supplementary Figure 11 CC-Type2-IL-Eg - HPLC traces from a gradient of 20 to 80% MeCN (0.1% TFA) in H_2O (0.1% TFA) (left, 220 and 280 nm) and MALDI-TOF MS (right). Calculated mass = 3245.9 Da, observed mass = 3247 Da.

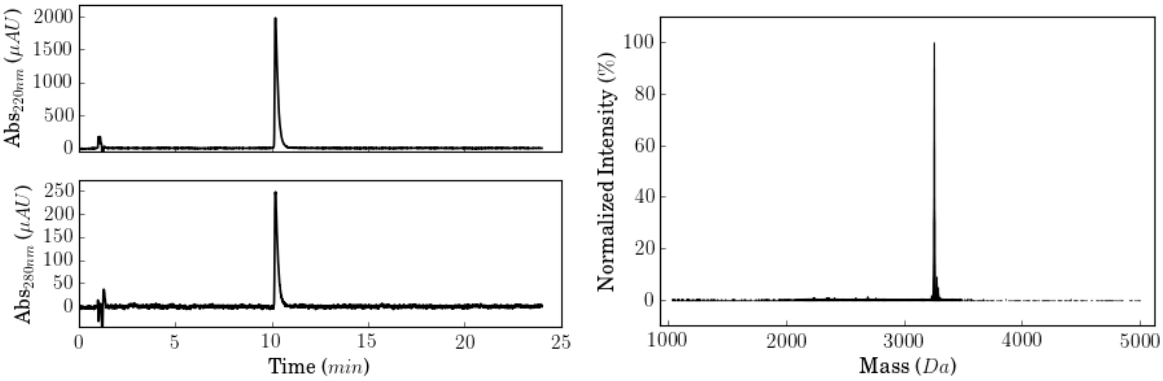

Supplementary Figure 12 CC-Type2-LL - HPLC traces from a gradient of 40 to 100% MeCN (0.1% TFA) in H_2O (0.1% TFA) (left, 220 and 280 nm) and MALDI-TOF MS (right). Calculated mass = 3245.9 Da, observed mass = 3247 Da.

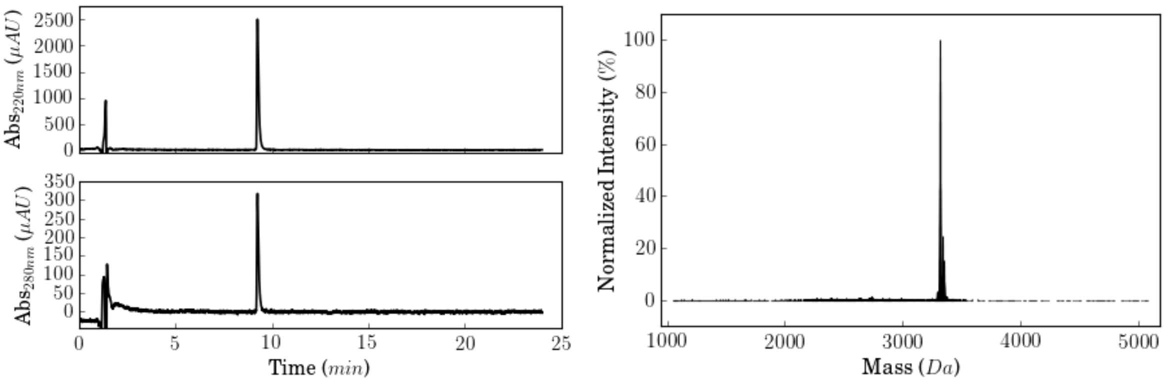

Supplementary Figure 13 CC-Type2-LL-Sg - HPLC traces from a gradient of 40 to 100% MeCN (0.1% TFA) in H_2O (0.1% TFA) (left, 220 and 280 nm) and MALDI-TOF MS (right). Calculated mass = 3309.9 Da, observed mass = 3311 Da.

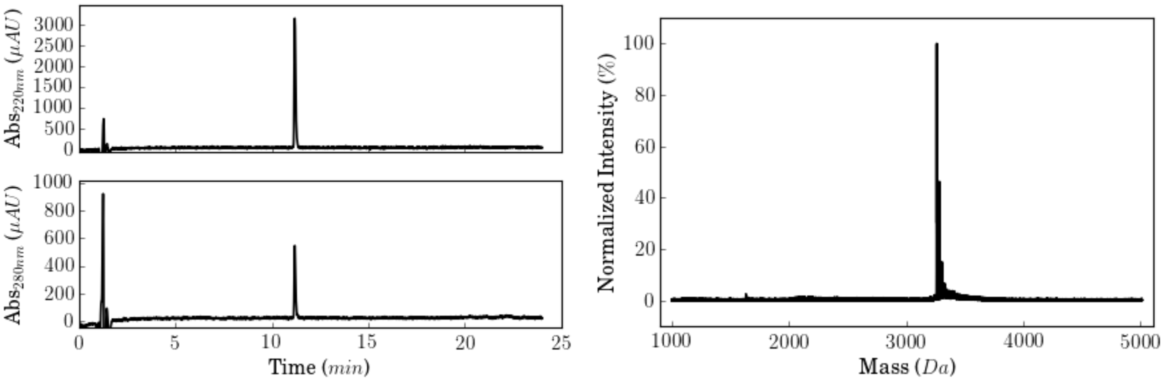

Supplementary Figure 14 CC-Type2-LL-L17Q - HPLC traces from a gradient of 20 to 80% MeCN (0.1% TFA) in H_2O (0.1% TFA) (left, 220 and 280 nm) and MALDI-TOF MS (right). Calculated mass = 3260.9 Da, observed mass = 3260 Da.

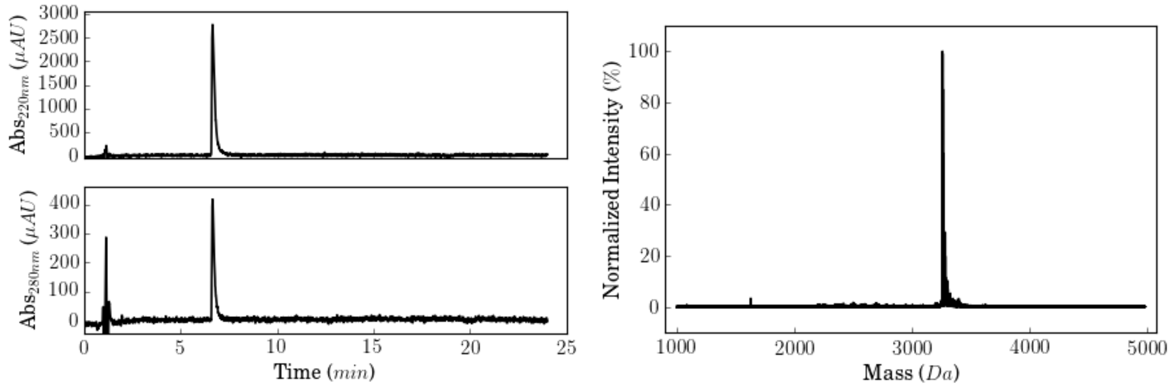

Supplementary Figure 15 CC-Type2-LL-L17E - HPLC traces from a gradient of 20 to 80% MeCN (0.1% TFA) in H_2O (0.1% TFA) (left, 220 and 280 nm) and MALDI-TOF MS (right). Calculated mass = 3261.8 Da, observed mass = 3263 Da.

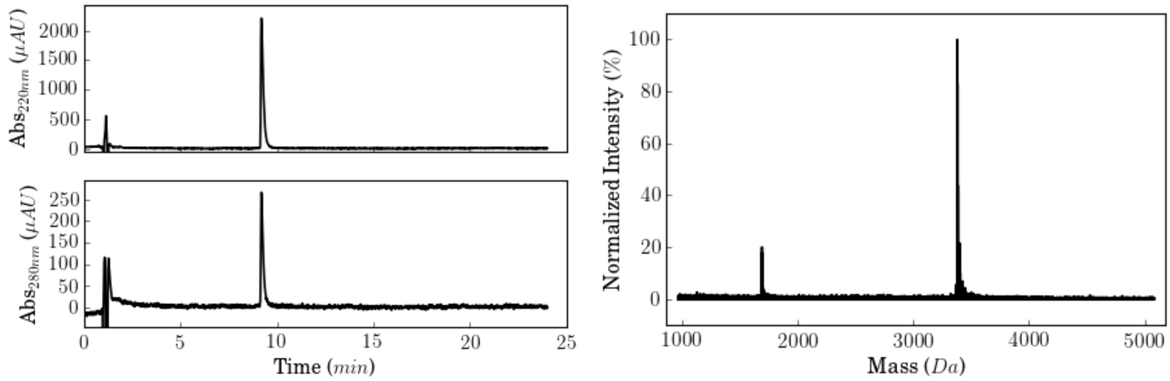

Supplementary Figure 16 CC-Type2-IF - HPLC traces from a gradient of 40 to 100% MeCN (0.1% TFA) in H_2O (0.1% TFA) (left, 220 and 280 nm) and MALDI-TOF MS (right). Calculated mass = 3381.8 Da, observed mass = 3382 Da.

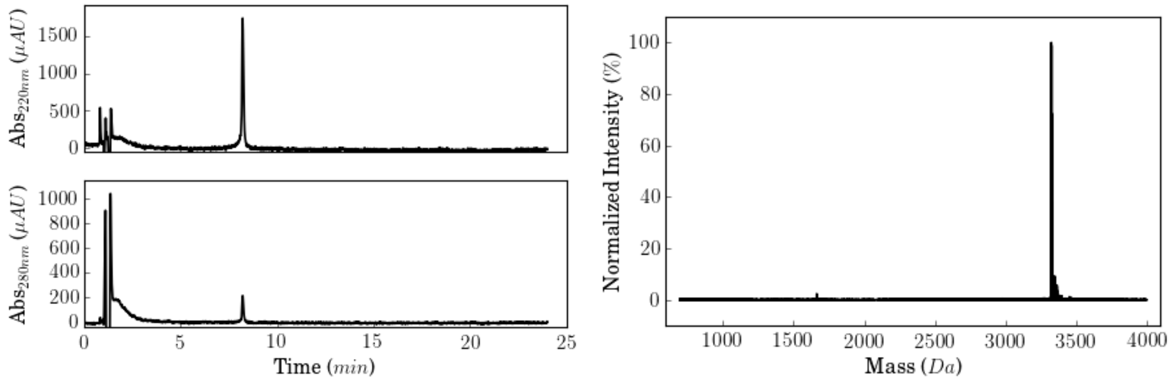

Supplementary Figure 17 CC-Type2-FV - HPLC traces from a gradient of 40 to 100% MeCN (0.1% TFA) in H_2O (0.1% TFA) (left, 220 and 280 nm) at 50 °C and MALDI-TOF MS (right). Calculated mass = 3325.8 Da, observed mass = 3325 Da.

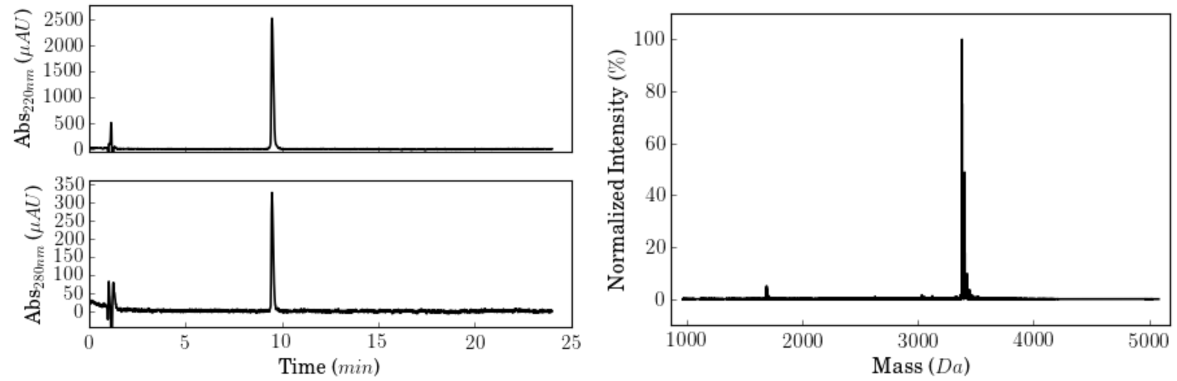

Supplementary Figure 18 CC-Type2-FI - HPLC traces from a gradient of 40 to 100% MeCN (0.1% TFA) in H_2O (0.1% TFA) (left, 220 and 280 nm) and MALDI-TOF MS (right). Calculated mass = 3381.8 Da, observed mass = 3382 Da.

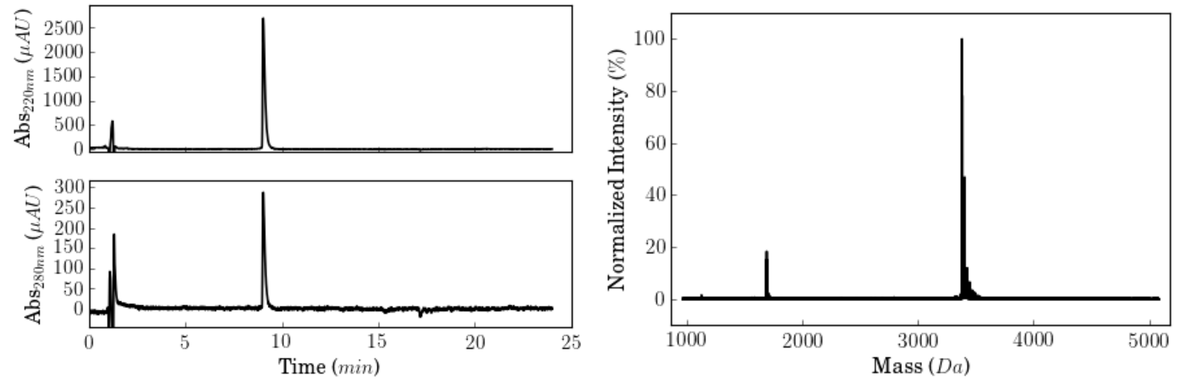

Supplementary Figure 19 CC-Type2-LF - HPLC traces from a gradient of 40 to 100% MeCN (0.1% TFA) in H_2O (0.1% TFA) (left, 220 and 280 nm) and MALDI-TOF MS (right). Calculated mass = 3381.8 Da, observed mass = 3381 Da.

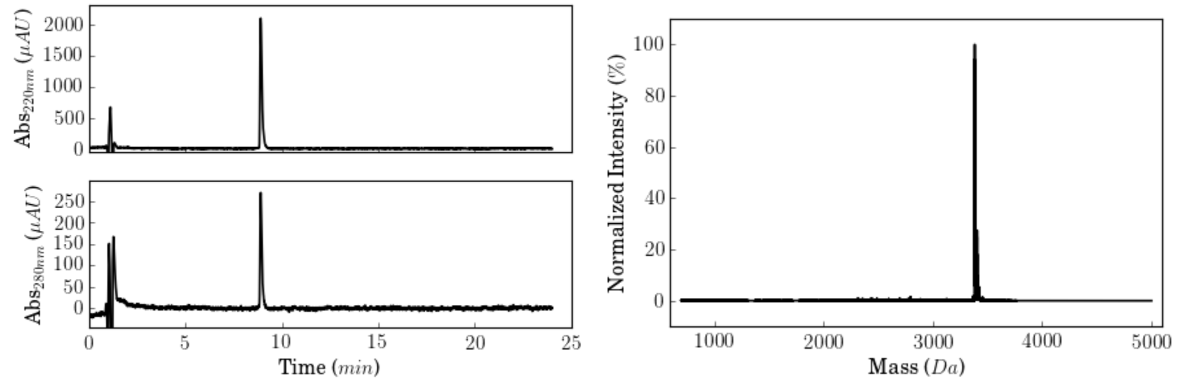

Supplementary Figure 20 CC-Type2-FL - HPLC traces from a gradient of 40 to 100% MeCN (0.1% TFA) in H_2O (0.1% TFA) (left, 220 and 280 nm) and MALDI-TOF MS (right). Calculated mass = 3381.8 Da, observed mass = 3383 Da.

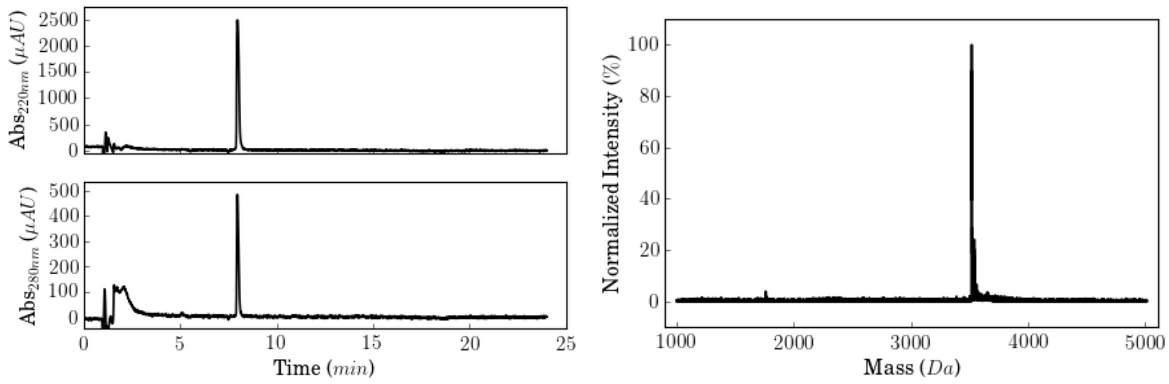

Supplementary Figure 21 CC-Type2-FF - HPLC traces from a gradient of 40 to 100% MeCN (0.1% TFA) in H_2O (0.1% TFA) (left, 220 and 280 nm) and MALDI-TOF MS (right). Calculated mass = 3517.8 Da, observed mass = 3518 Da.

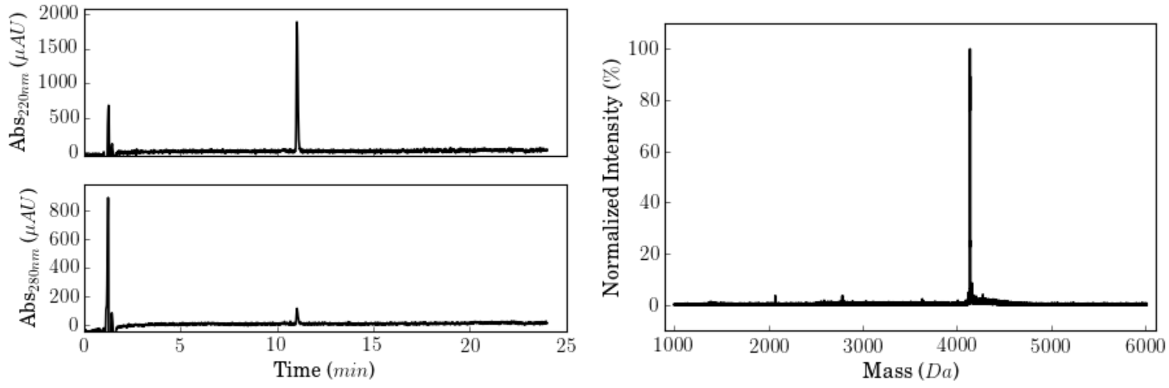

Supplementary Figure 22 5H2L_2.1-I9L - HPLC traces from a gradient of 20 to 80% MeCN (0.1% TFA) in H_2O (0.1% TFA) (left, 220 and 280 nm) and MALDI-TOF MS (right). Calculated mass = 4141.5 Da, observed mass = 4140 Da.

Circular dichroism (CD)

The circular dichrosim experiments for CC-Type2-II, CC-Type2-VI, CC-Type2-LI and CC-Type2-LV (formerly known as AIKEIA, AVKEIA, CC-Hept and ALKEVA) have been reported previously.[1]

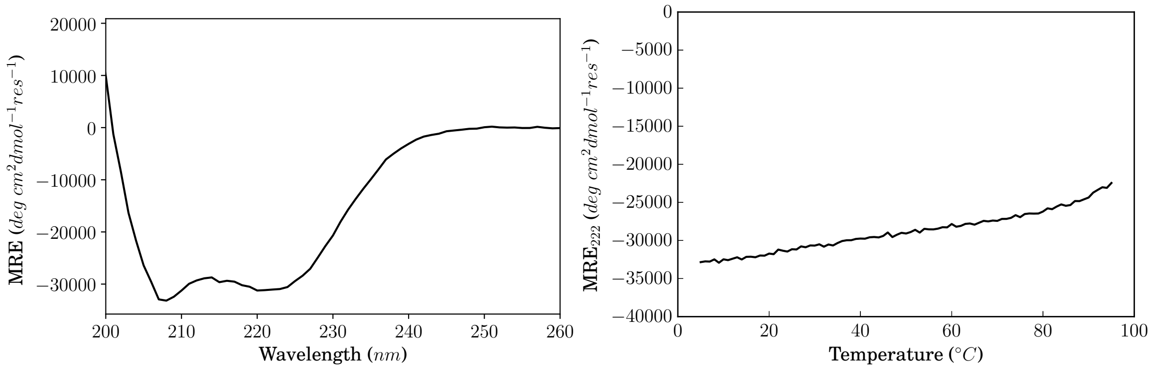

Supplementary Figure 23 CC-Type2-IV - CD spectrum at 20 °C (left) and thermal denaturation profile monitored at 222 nm (right). Conditions: 10 μM peptide concentration, PBS (pH 7.4).

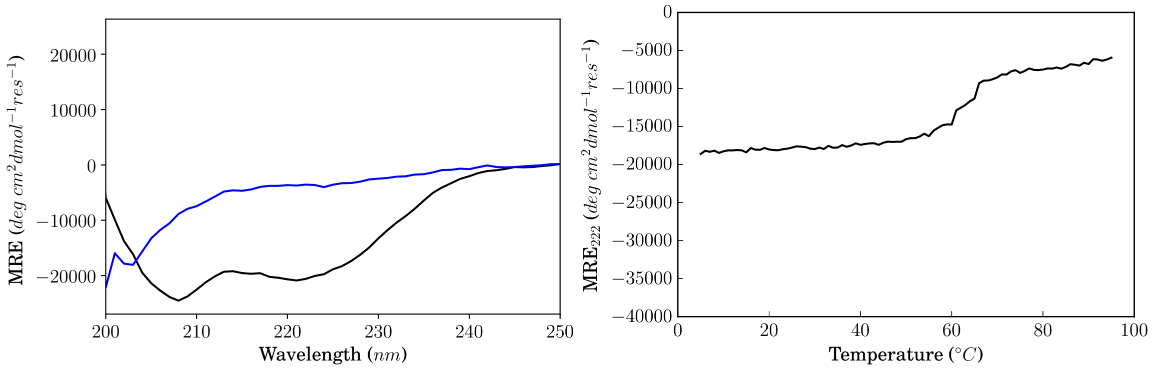

Supplementary Figure 24 CC-Type2-VV - CD spectrum at 20 °C (left) and thermal denaturation profile monitored at 222 nm (right). Blue-line plot reports 20 °C scan upon cooling the sample from 95 °C. Conditions: 10 μM peptide concentration, PBS (pH 7.4).

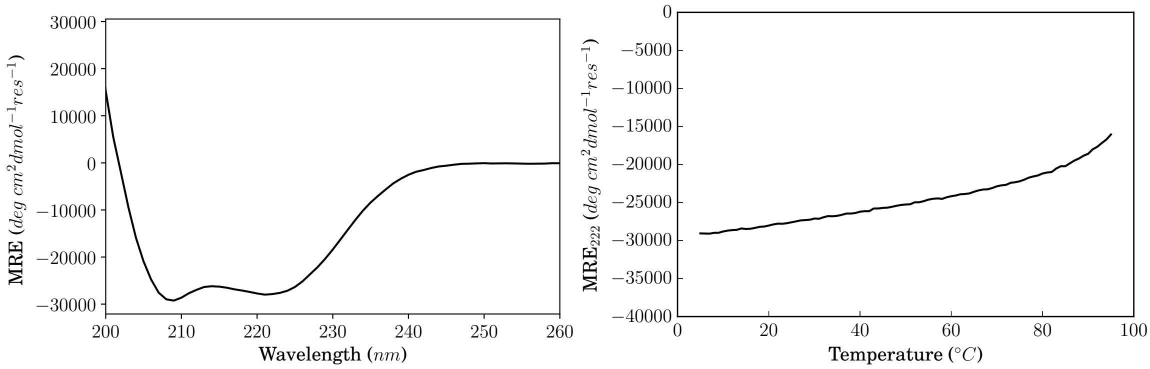

Supplementary Figure 25 CC-Type2-deLI - CD spectrum at 20 °C (left) and thermal denaturation profile monitored at 222 nm (right). Conditions: 10 μM peptide concentration, PBS (pH 7.4).

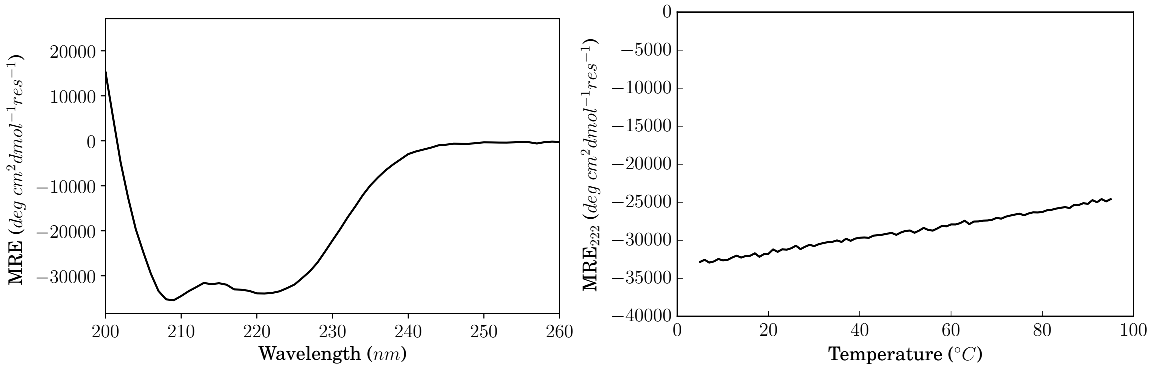

Supplementary Figure 26 CC-Type2-VL - CD spectrum at 20 °C (left) and thermal denaturation profile monitored at 222 nm (right). Conditions: 10 μM peptide concentration, PBS (pH 7.4).

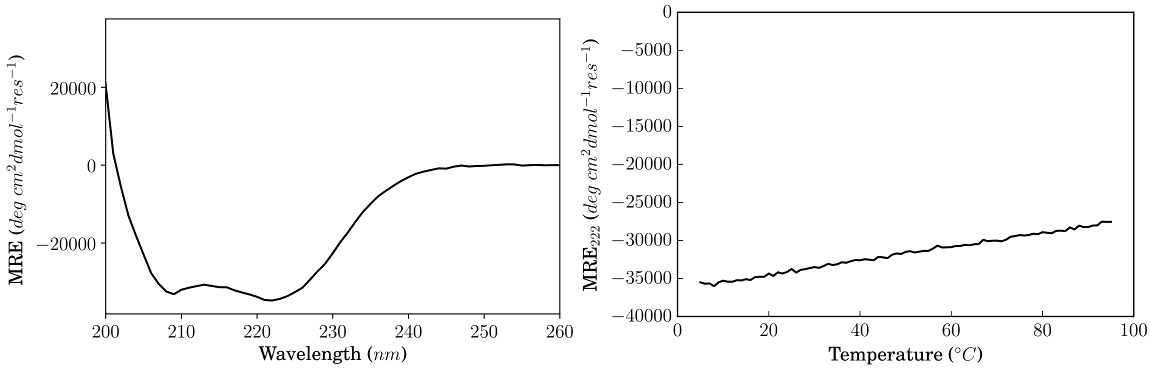

Supplementary Figure 27 CC-Type2-IL-Sg - CD spectrum at 20 °C (left) and thermal denaturation profile monitored at 222 nm (right). Conditions: 10 μM peptide concentration, PBS (pH 7.4).

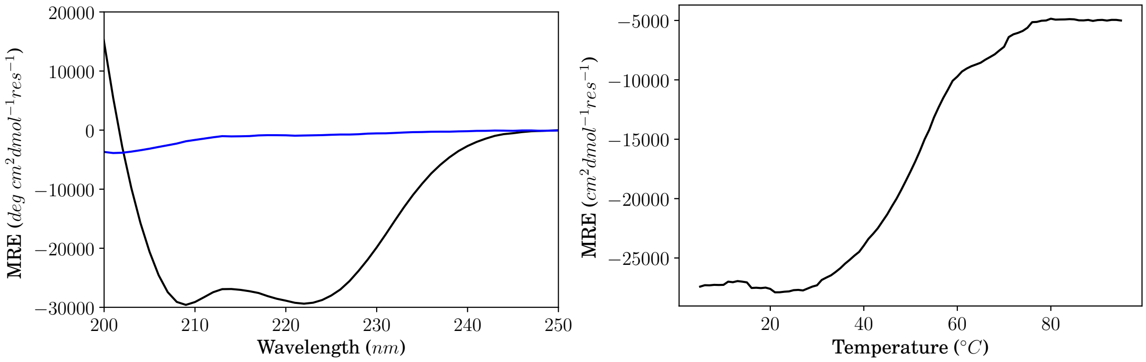

Supplementary Figure 28 CC-Type2-IL-Sg-L17E - CD spectrum at 20 °C (left) and thermal denaturation profile monitored at 222 nm (right). Blue-line plot reports 20 °C scan upon cooling the sample from 95 °C. Conditions: 100 μM peptide concentration, PBS (pH 7.4).

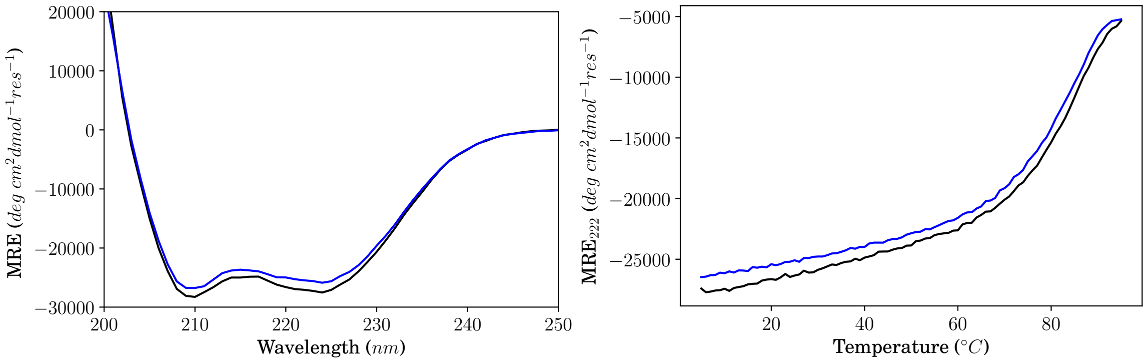

Supplementary Figure 29 CC-Type2-IL-Eg - CD spectrum at 20 °C (left) and thermal denaturation profile monitored at 222 nm (right). Blue-line plot reports 20 °C scan upon cooling the sample from 95 °C (left) and additional blue-line plot on thermal denaturation profile shows cooling from 95 °C to 5 °C to highlight that some peptide had precipitated. Conditions: 10 μM peptide concentration, PBS (pH 7.4).

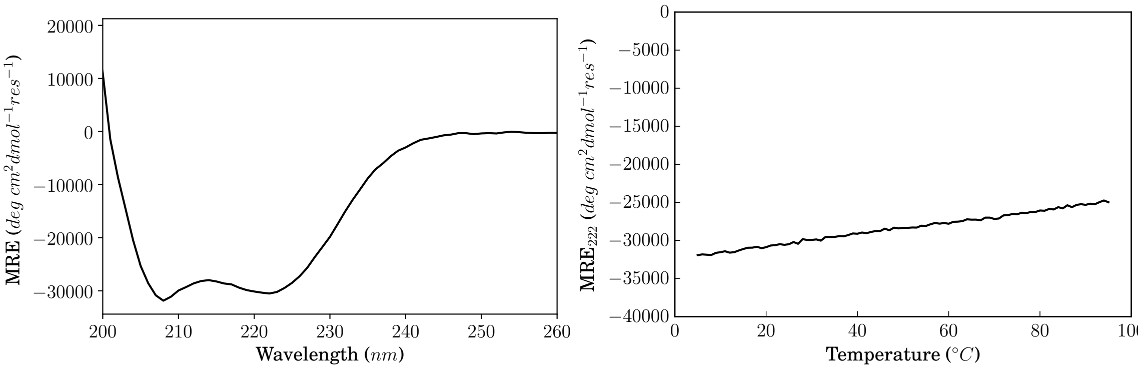

Supplementary Figure 30 CC-Type2-LL - CD spectrum at 20 °C (left) and thermal denaturation profile monitored at 222 nm (right). Conditions: 10 μM peptide concentration, PBS (pH 7.4).

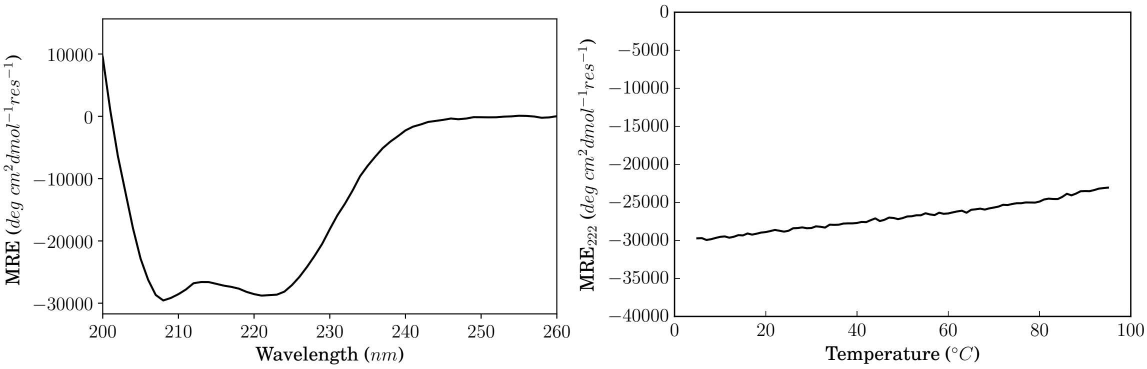

Supplementary Figure 31 CC-Type2-LL-Sg - CD spectrum at 20 °C (left) and thermal denaturation profile monitored at 222 nm (right). Conditions: 10 μM peptide concentration, PBS (pH 7.4).

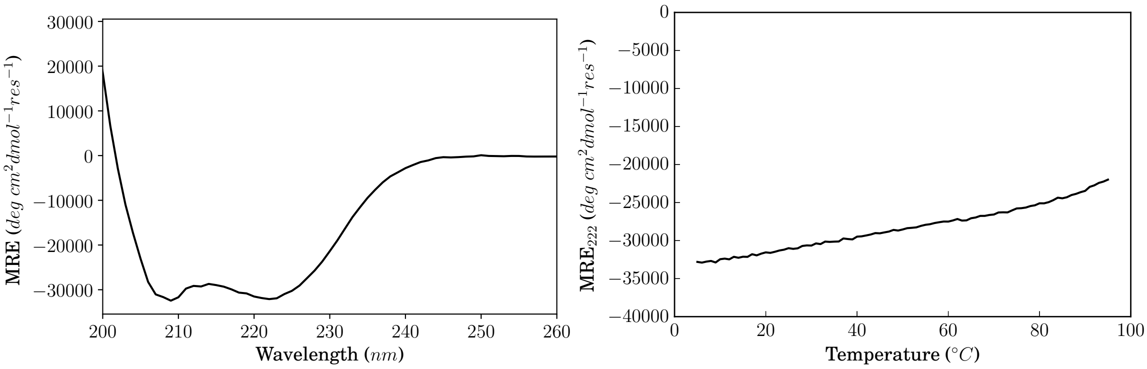

Supplementary Figure 32 CC-Type2-LL-L17Q - CD spectrum at 20 °C (left) and thermal denaturation profile monitored at 222 nm (right). Conditions: 10 μM peptide concentration, PBS (pH 7.4).

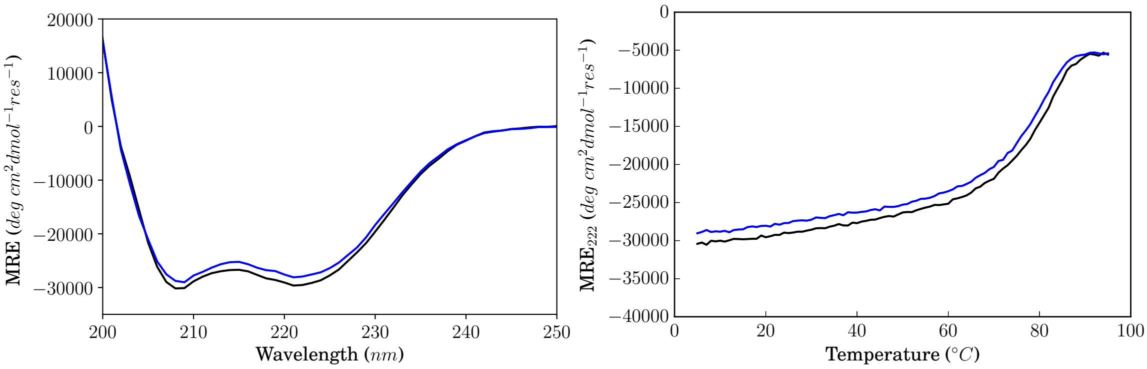

Supplementary Figure 33 CC-Type2-LL-L17E - CD spectrum at 20 °C (left) and thermal denaturation profile monitored at 222 nm (right). Blue-line plot reports 20 °C scan upon cooling the sample from 95 °C (left) and additional blue-line plot on thermal denaturation profile shows cooling from 95 °C to 5 °C to highlight that some peptide had precipitated. Conditions: 10 μM peptide concentration, PBS (pH 7.4).

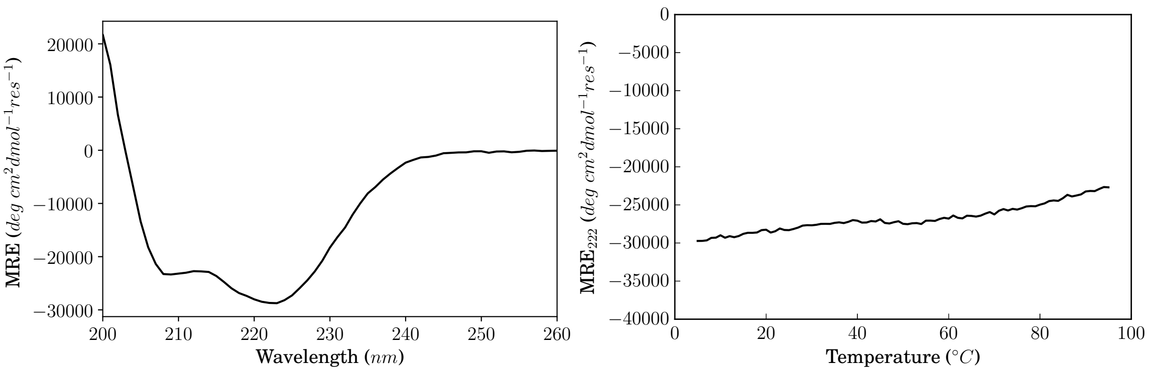

Supplementary Figure 34 CC-Type2-IF - CD spectrum at 20 °C (left) and thermal denaturation profile monitored at 222 nm (right). Conditions: 10 μM peptide concentration, PBS (pH 7.4).

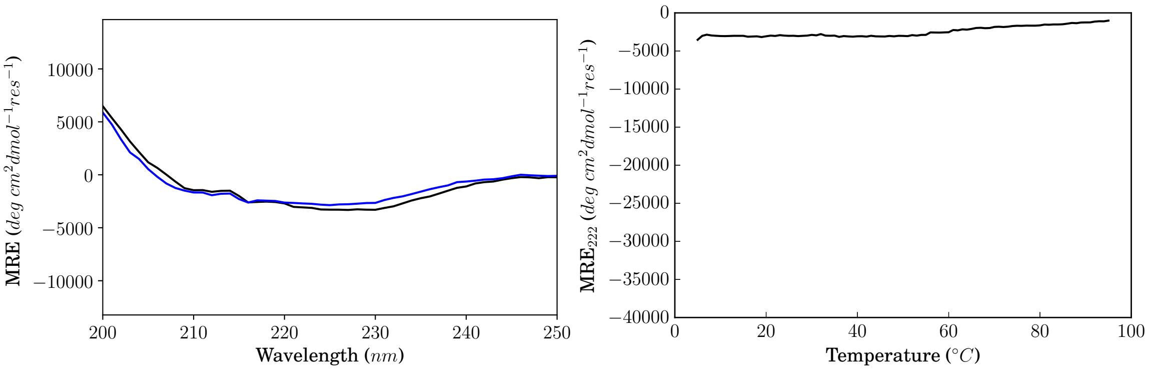

Supplementary Figure 35 CC-Type2-FV - CD spectrum at 20 °C (left) and thermal denaturation profile monitored at 222 nm (right). Blue-line plot reports 20 °C scan upon cooling the sample from 95 °C. Conditions: 100 μM peptide concentration, PBS (pH 7.4).

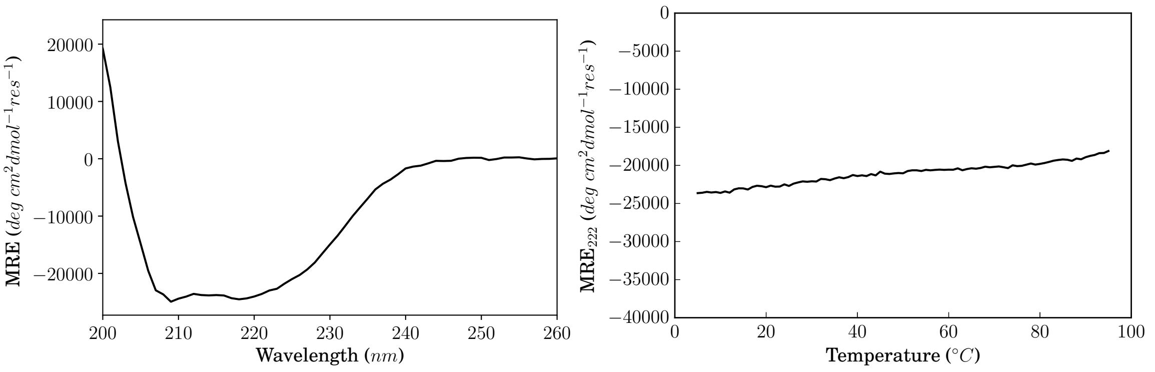

Supplementary Figure 36 CC-Type2-FI - CD spectrum at 20 °C (left) and thermal denaturation profile monitored at 222 nm (right). Conditions: 10 μM peptide concentration, PBS (pH 7.4).

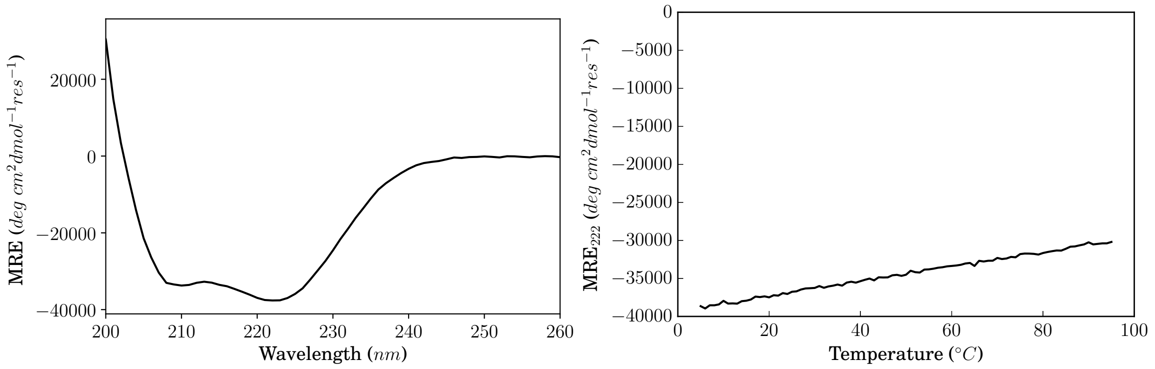

Supplementary Figure 37 CC-Type2-LF - CD spectrum at 20 °C (left) and thermal denaturation profile monitored at 222 nm (right). Conditions: 10 μM peptide concentration, PBS (pH 7.4).

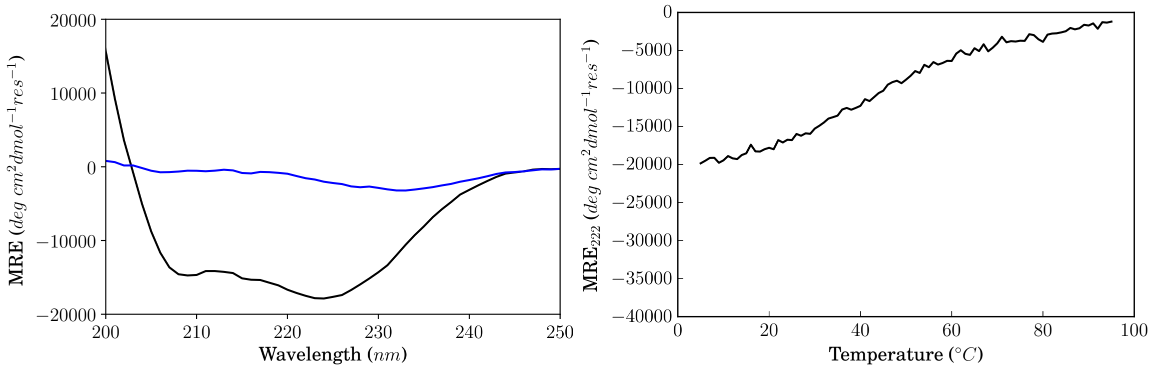

Supplementary Figure 38 CC-Type2-FL - CD spectrum at 20 °C (left) and thermal denaturation profile monitored at 222 nm (right). Blue-line plot reports 20 °C scan upon cooling the sample from 95 °C. Conditions: 100 μM peptide concentration, PBS (pH 7.4).

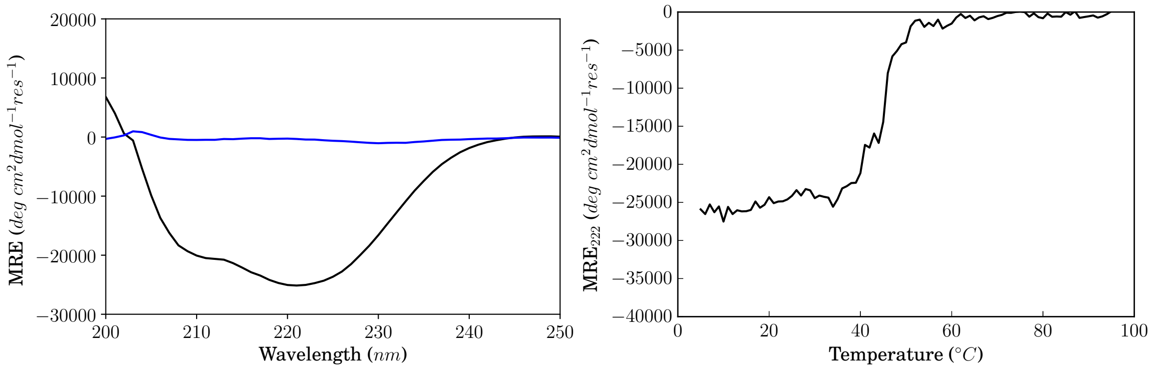

Supplementary Figure 39 CC-Type2-FF - CD spectrum at 20 °C (left) and thermal denaturation profile monitored at 222 nm (right). Blue-line plot reports 20 °C scan upon cooling the sample from 95 °C. Conditions: 10 μM peptide concentration, PBS (pH 7.4).

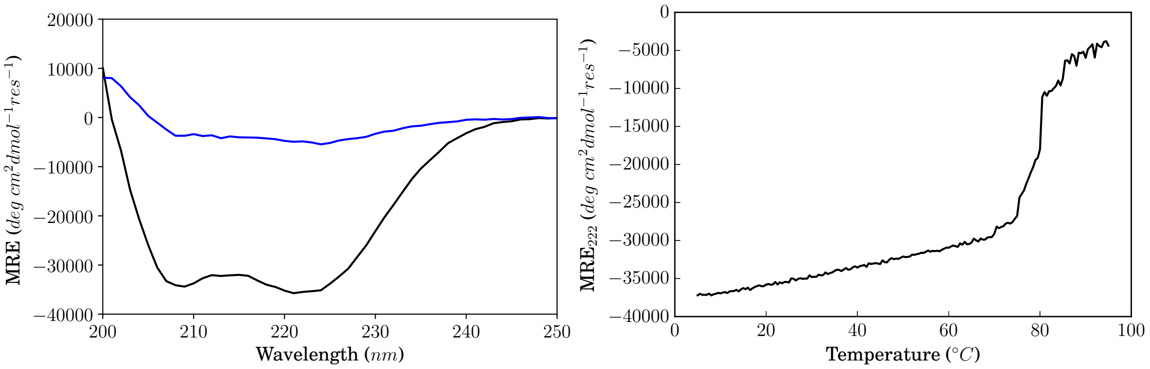

Supplementary Figure 40 5H2L_ 2.1-I9L - CD spectrum at 20 °C (left) and thermal denaturation profile monitored at 222 nm (right). Blue-line plot reports 20 °C scan upon cooling the sample from 95 °C. Conditions: 10 μM peptide concentration, PBS (pH 7.4).

Analytical ultracentrifugation (AUC)

The sedimentation-velocity experiments for CC-Type2-II, CC-Type2-LI, CC-Type2-LV and CC-Type2-VI (formerly known as AIKEIA, CC-Hept, ALKEVA and AVKEIA) have been reported previously.[1] Due to low α helicity, the AUC experiments were not conducted for the sequence CC-Type2-FV. CC-Type2-FL aggregated at 3,000 rpm and CC-Type2-FF slowly precipitated during the SE experiment.

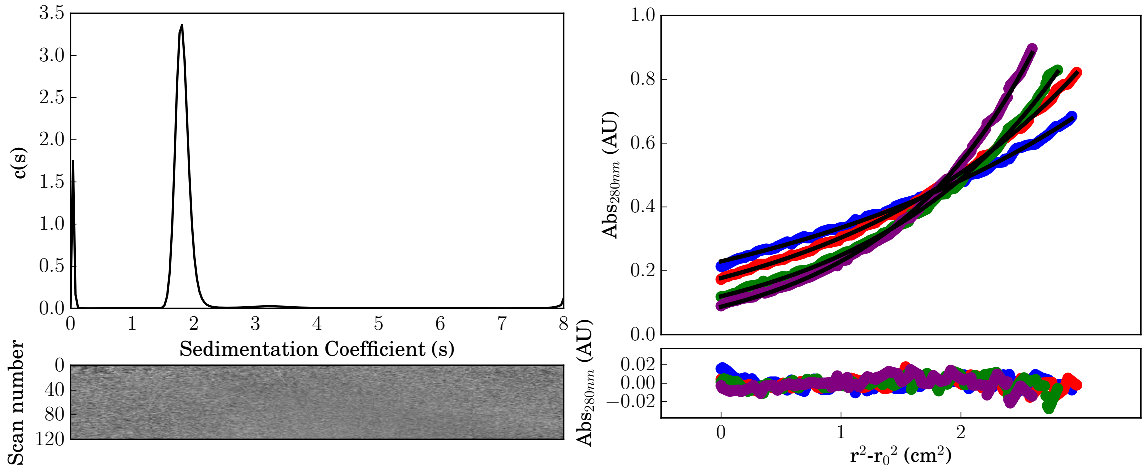

Supplementary Figure 41 CC-Type2-IV - AUC data and fits (top) and residuals (bottom) ($\bar{v}$ = 0.770 cm^3 g^{-1}). Left: continuous c(s) distribution from sedimentation-velocity data at 50k rpm returning s = 1.817 S, s20,w = 2.182 S, f/f0 = 1.261 and mw = 21,660 Da (6.8 × monomer mass) at 95% confidence level. Conditions: 150 μM peptide concentration, PBS (pH 7.4). Residuals for sedimentation-velocity experiments are shown as a bitmap in which the greyscale shade indicates the difference between the fit and raw data. Scans are ordered vertically, with earlier scans at the top. The horizontal axis is the radial range over which the data were fitted. Right: sedimentation-equilibrium data (top, dots) and fitted single-ideal species model curves at 18k (blue), 21k (red), 24k (green) and 27k (purple) rpm. The fit returns a mass of 21,150 Da (6.6 × monomer mass, 95% confidence limits 21,088 – 21,252). Bottom: residuals for the above fits using the same colour scheme as above. Conditions: 70 μM peptide concentration, PBS (pH 7.4).

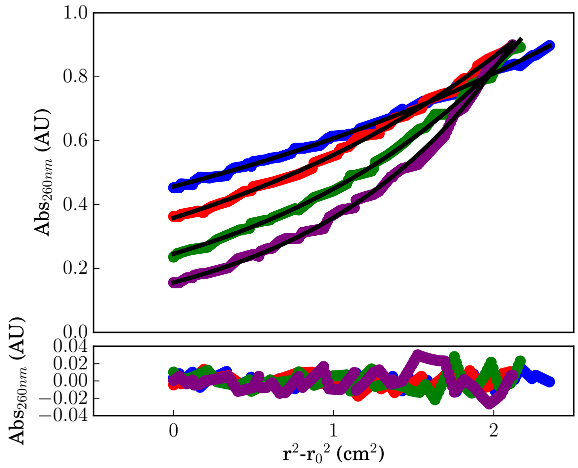

Supplementary Figure 42 CC-Type2-II - AUC data and fits (top) and residuals (bottom) ($\bar{\upsilon}$ = 0.777 cm^3 g^{-1}). sedimentation-equilibrium data (top, dots) and fitted single-ideal species model curves at 18 k (blue), 22 k (red), 26 k (green) and 30 k (purple) rpm. The fit returns a mass of 18,620 Da (5.7 × monomer mass, 95% confidence limits 18,525 – 18,709). Bottom: residuals for the above fits using the same colour scheme as above. Conditions: 150 μM peptide concentration, PBS (pH 7.4).

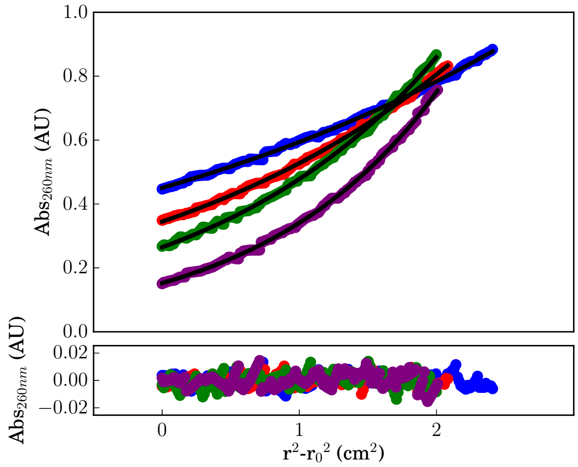

Supplementary Figure 43 CC-Type2-VI - AUC data and fits (top) and residuals (bottom) ($\bar{\upsilon}$ = 0.770 cm^3 g^{-1}). sedimentation-equilibrium data (top, dots) and fitted single-ideal species model curves at 18 k (blue), 22 k (red), 26 k (green) and 30 k (purple) rpm. The fit returns a mass of 16,940 Da (5.3 × monomer mass, 95% confidence limits 16,884 – 17,003). Bottom: residuals for the above fits using the same colour scheme as above. Conditions: 150 μM peptide concentration, PBS (pH 7.4).

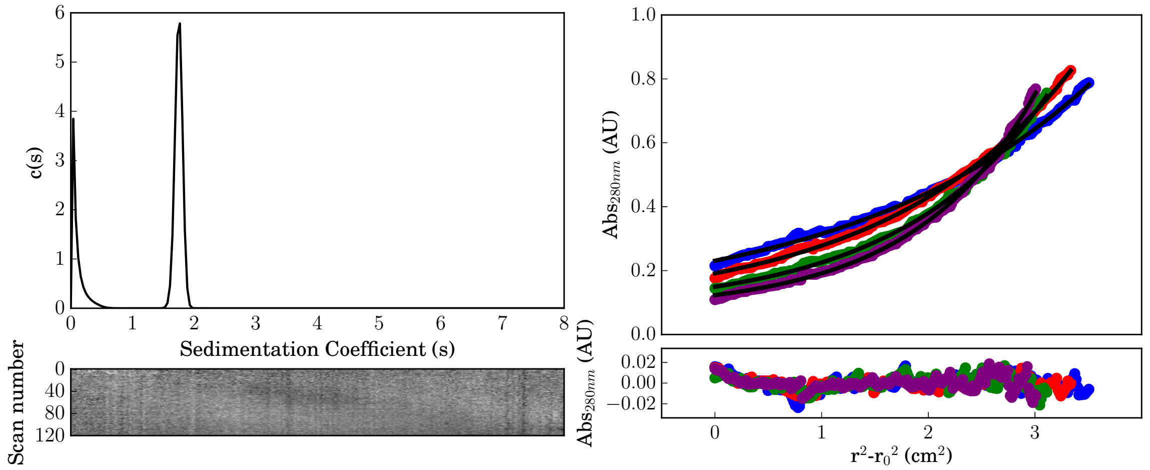

Supplementary Figure 44 CC-Type2-VV - AUC data and fits (top) and residuals (bottom) ($\bar{v}$ = 0.762 cm^3 g^{-1}). Left: continuous c(s) distribution from sedimentation-velocity data at 50k rpm returning s = 1.750 S, s20,w = 2.034 S, f/f0 = 1.214 and mw = 18,320 Da (5.8 × monomer mass) at 95% confidence level. Conditions: 150 μM peptide concentration, PBS (pH 7.4). Residuals for sedimentation-velocity experiments are shown as a bitmap in which the greyscale shade indicates the difference between the fit and raw data. Scans are ordered vertically, with earlier scans at the top. The horizontal axis is the radial range over which the data were fitted. Right: sedimentation-equilibrium data (top, dots) and fitted single-ideal species model curves at 21k (blue), 24k (red), 27k (green) and 30k (purple) rpm. The fit returns a mass of 18,760 Da (6.0 × monomer mass, 95% confidence limits 18,513 – 18,798). Bottom: residuals for the above fits using the same colour scheme as above. Conditions: 70 μM peptide concentration, PBS (pH 7.4).

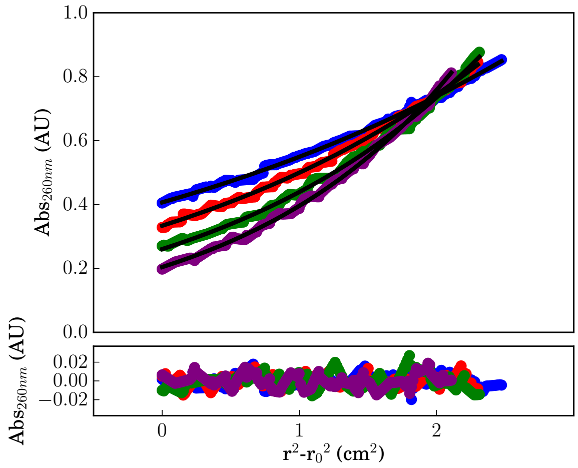

Supplementary Figure 45 CC-Type2-LI - AUC data and fits (top) and residuals (bottom) ($\bar{v}$ = 0.777 cm^3 g^{-1}). sedimentation-equilibrium data (top, dots) and fitted single-ideal species model curves at 18 k (blue), 21 k (red), 24 k (green) and 27 k (purple) rpm. The fit returns a mass of 17,770 Da (5.3 × monomer mass, 95% confidence limits 16,771 – 18,791). Bottom: residuals for the above fits using the same colour scheme as above. Conditions: 150 μM peptide concentration, PBS (pH 7.4).

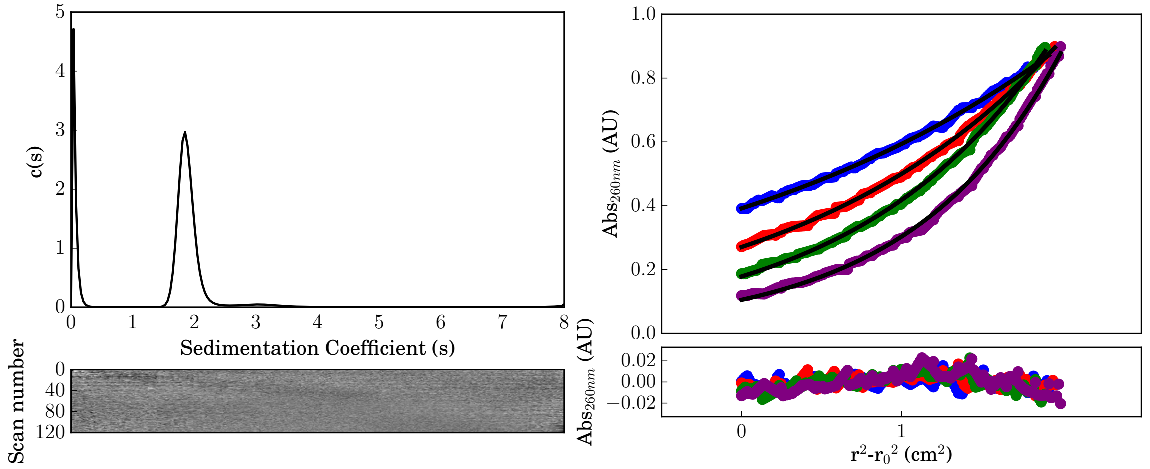

Supplementary Figure 46 CC-Type2-deLI - AUC data and fits (top) and residuals (bottom) ($\bar{v}$ = 0.777 cm^3 g^{-1}). Left: continuous c(s) distribution from sedimentation-velocity data at 50k rpm returning s = 1.876 S, s20,w = 2.329 S, f/f0 = 1.199 and mw = 22,234 Da (6.9 × monomer mass) at 95% confidence level. Conditions: 150 μM peptide concentration, PBS (pH 7.4). Residuals for sedimentation-velocity experiments are shown as a bitmap in which the greyscale shade indicates the difference between the fit and raw data. Scans are ordered vertically, with earlier scans at the top. The horizontal axis is the radial range over which the data were fitted. Right: sedimentation-equilibrium data (top, dots) and fitted single-ideal species model curves at 20 k (blue), 24 k (red), 28 k (green) and 32 k (purple) rpm. The fit returns a mass of 21,390 Da (6.6 × monomer mass, 95% confidence limits 21,230 – 21,429). Bottom: residuals for the above fits using the same colour scheme as above. Conditions: 150 μM peptide concentration, PBS (pH 7.4).

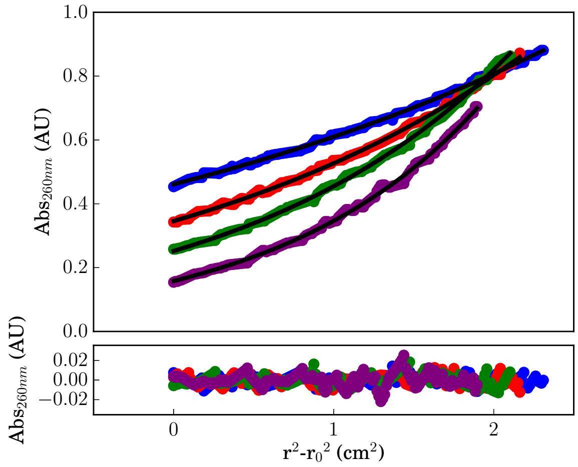

Supplementary Figure 47 CC-Type2-LV - AUC data and fits (top) and residuals (bottom) ($\bar{v}$ = 0.770 cm^3 g^{-1}). sedimentation-equilibrium data (top, dots) and fitted single-ideal species model curves at 18 k (blue), 21 k (red), 24 k (green) and 27 k (purple) rpm. The fit returns a mass of 17,180 Da (5.4 × monomer mass, 95% confidence limits 17,125 – 17,224). Bottom: residuals for the above fits using the same colour scheme as above. Conditions: 150 μM peptide concentration, PBS (pH 7.4).

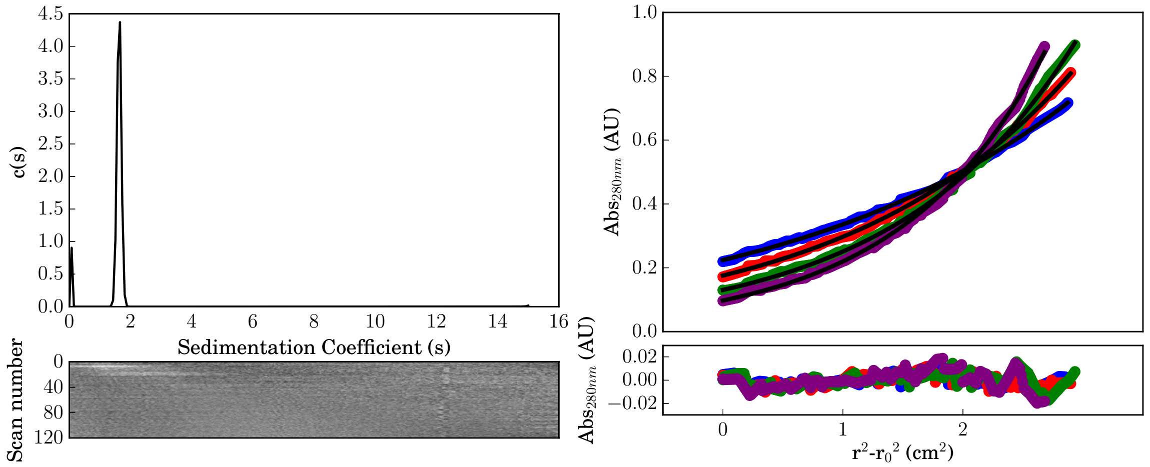

Supplementary Figure 48 CC-Type2-VL - AUC data and fits (top) and residuals (bottom) ($\bar{v}$ = 0.770 cm^3 g^{-1}). Left: continuous c(s) distribution from sedimentation-velocity data at 50k rpm returning s = 1.630 S, s20,w = 1.958 S, f/f0 = 1.133 and mw = 15,681 Da (4.9 × monomer mass) at 95% confidence level. Conditions: 150 μM peptide concentration, PBS (pH 7.4). Residuals for sedimentation-velocity experiments are shown as a bitmap in which the greyscale shade indicates the difference between the fit and raw data. Scans are ordered vertically, with earlier scans at the top. The horizontal axis is the radial range over which the data were fitted. Right: sedimentation-equilibrium data (top, dots) and fitted single-ideal species model curves at 21k (blue), 24k (red), 27k (green) and 30k (purple) rpm. The fit returns a mass of 18,080 Da (5.7 × monomer mass, 95% confidence limits 17,996 – 18,209). Bottom: residuals for the above fits using the same colour scheme as above. Conditions: 70 μM peptide concentration, PBS (pH 7.4).

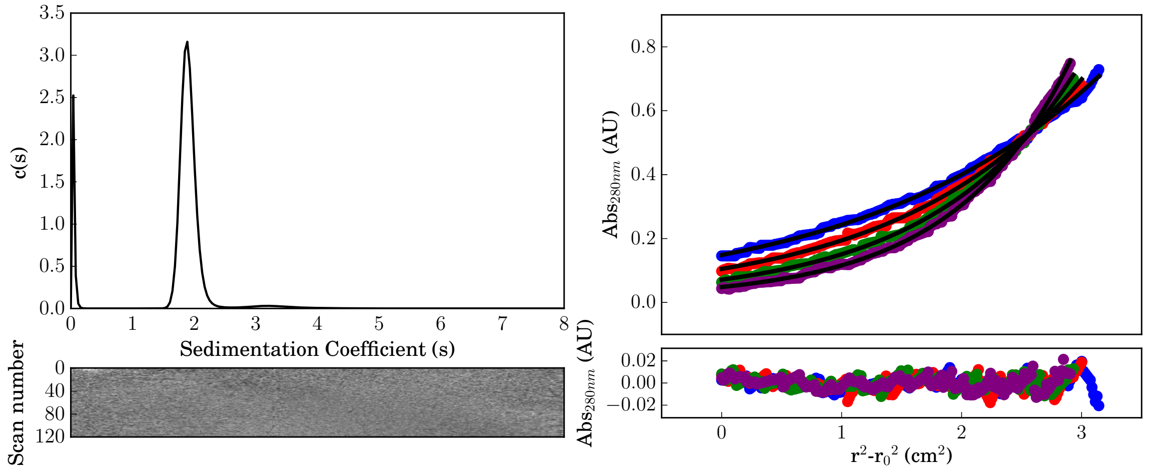

Supplementary Figure 49 CC-Type2-IL-Sg - AUC data and fits (top) and residuals (bottom) ($\bar{v}$ = 0.765 cm^3 g^{-1}). Left: continuous c(s) distribution from sedimentation-velocity data at 50k rpm returning s = 1.901 S, s20,w = 2.231 S, f/f0 = 1.222 and mw = 21,271 Da (6.4 × monomer mass) at 95% confidence level. Conditions: 150 μM peptide concentration, PBS (pH 7.4). Residuals for sedimentation-velocity experiments are shown as a bitmap in which the greyscale shade indicates the difference between the fit and raw data. Scans are ordered vertically, with earlier scans at the top. The horizontal axis is the radial range over which the data were fitted. Right: sedimentation-equilibrium data (top, dots) and fitted single-ideal species model curves at 21k (blue), 24k (red), 27k (green) and 30k (purple) rpm. The fit returns a mass of 21,950 Da (6.6 × monomer mass, 95% confidence limits 21,816 – 22,078). Bottom: residuals for the above fits using the same colour scheme as above. Conditions: 70 μM peptide concentration, PBS (pH 7.4).

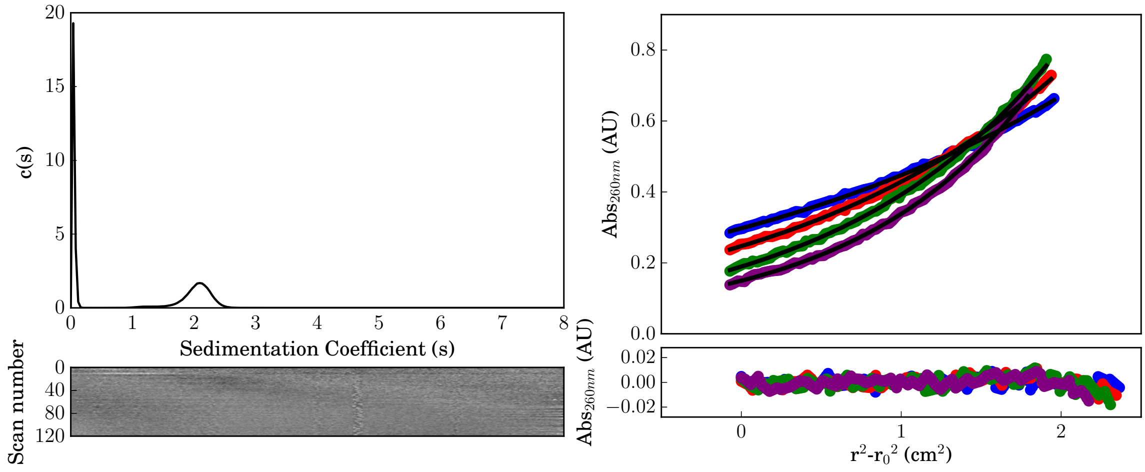

Supplementary Figure 50 CC-Type2-IL-Sg-L17E - AUC data and fits (top) and residuals (bottom) ($\bar{\nu}$ = 0.765 cm^3 g^{-1}). Left: continuous c(s) distribution from sedimentation-velocity data at 50k rpm returning s = 2.054 S, s20,w = 2.321 S, f/f0 = 1.121 and mw = 19,710 Da (5.9 × monomer mass) at 95% confidence level. Conditions: 150 μM peptide concentration, PBS (pH 7.4). Residuals for sedimentation-velocity experiments are shown as a bitmap in which the greyscale shade indicates the difference between the fit and raw data. Scans are ordered vertically, with earlier scans at the top. The horizontal axis is the radial range over which the data were fitted. Right: sedimentation-equilibrium data (top, dots) and fitted single-ideal species model curves at 18 k (blue), 21 k (red), 24 k (green) and 27 k (purple) rpm. The fit returns a mass of 19,760 Da (5.9 × monomer mass, 95% confidence limits 19,638 – 19,925). Bottom: residuals for the above fits using the same colour scheme as above. Conditions: 150 μM peptide concentration, PBS (pH 7.4).

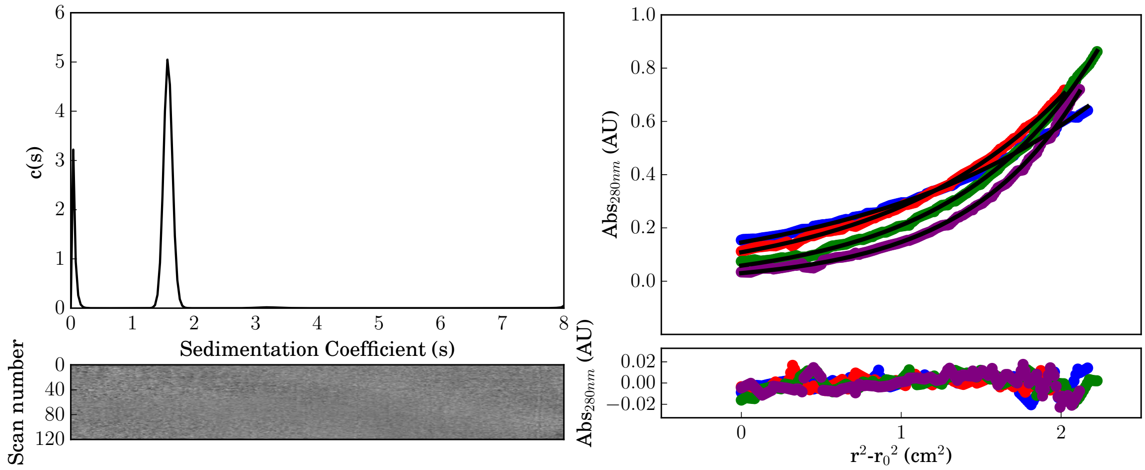

Supplementary Figure 51 CC-Type2-IL-Eg - AUC data and fits (top) and residuals (bottom) ($\bar{\nu}$ = 0.777 cm^3 g^{-1}). Left: continuous c(s) distribution from sedimentation-velocity data at 50k rpm returning s = 1.578 S, s20,w = 1.957 S, f/f0 = 1.236 and mw = 17,926 Da (5.5 × monomer mass) at 95% confidence level. Conditions: 150 μM peptide concentration, PBS (pH 7.4). Residuals for sedimentation-velocity experiments are shown as a bitmap in which the greyscale shade indicates the difference between the fit and raw data. Scans are ordered vertically, with earlier scans at the top. The horizontal axis is the radial range over which the data were fitted. Right: sedimentation-equilibrium data (top, dots) and fitted single-ideal species model curves at 28 k (blue), 32 k (red), 36 k (green) and 40 k (purple) rpm. The fit returns a mass of 17,410 Da (5.5 × monomer mass, 95% confidence limits 17,352 – 17,467). Bottom: residuals for the above fits using the same colour scheme as above. Conditions: 150 μM peptide concentration, PBS (pH 7.4).

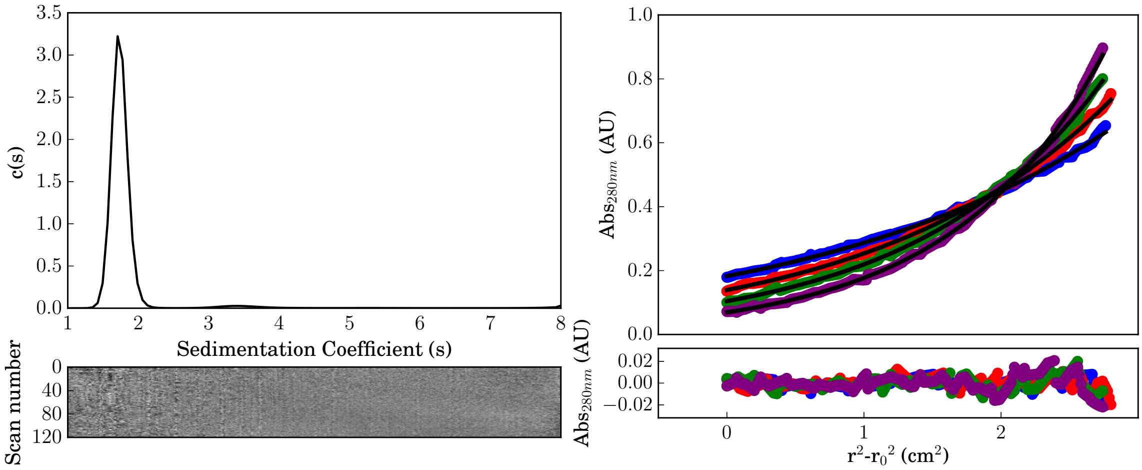

Supplementary Figure 52 CC-Type2-LL - AUC data and fits (top) and residuals (bottom) ($\bar{v}$ = 0.777 cm^3 g^{-1}). Left: continuous c(s) distribution from sedimentation-velocity data at 50k rpm returning s = 1.737 S, s20,w = 2.154 S, f/f0 = 1.201 and mw = 19,825 Da (6.1 × monomer mass) at 95% confidence level. Conditions: 150 μM peptide concentration, PBS (pH 7.4). Residuals for sedimentation-velocity experiments are shown as a bitmap in which the greyscale shade indicates the difference between the fit and raw data. Scans are ordered vertically, with earlier scans at the top. The horizontal axis is the radial range over which the data were fitted. Right: sedimentation-equilibrium data (top, dots) and fitted single-ideal species model curves at 21k (blue), 24k (red), 27k (green) and 30k (purple) rpm. The fit returns a mass of 20,520 Da (6.3 × monomer mass, 95% confidence limits 20,358 – 20,691). Bottom: residuals for the above fits using the same colour scheme as above. Conditions: 70 μM peptide concentration, PBS (pH 7.4).

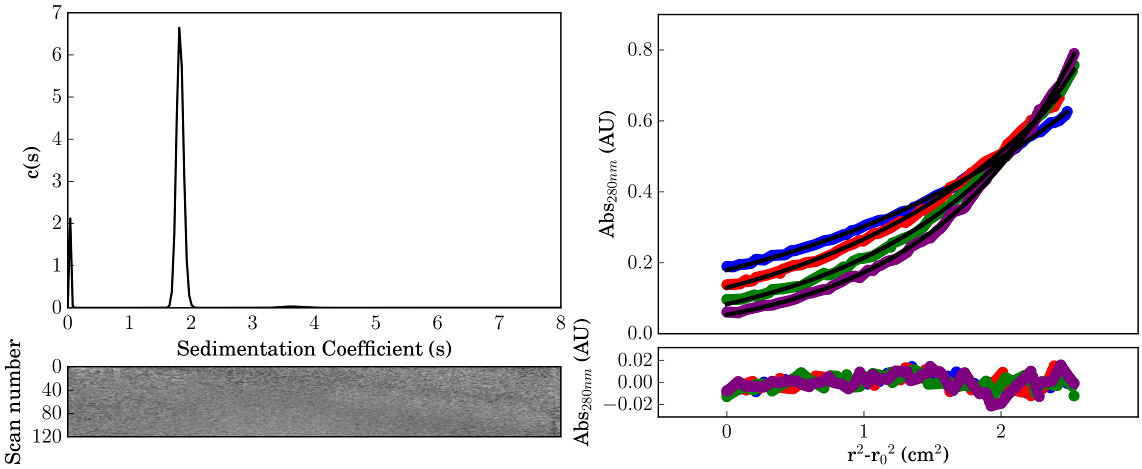

Supplementary Figure 53 CC-Type2-LL-Sg - AUC data and fits (top) and residuals (bottom) ($\bar{v}$ = 0.765 cm^3 g^{-1}). Left: continuous c(s) distribution from sedimentation-velocity data at 50k rpm returning s = 1.822 S, s20,w = 2.138 S, f/f0 = 1.237 and mw = 20,338 Da (6.1 × monomer mass) at 95% confidence level. Conditions: 150 μM peptide concentration, PBS (pH 7.4). Residuals for sedimentation-velocity experiments are shown as a bitmap in which the greyscale shade indicates the difference between the fit and raw data. Scans are ordered vertically, with earlier scans at the top. The horizontal axis is the radial range over which the data were fitted. Right: sedimentation-equilibrium data (top, dots) and fitted single-ideal species model curves at 21k (blue), 24k (red), 27k (green) and 30k (purple) rpm. The fit returns a mass of 19,670 Da (5.9 × monomer mass, 95% confidence limits 19,536 – 19,803). Bottom: residuals for the above fits using the same colour scheme as above. Conditions: 70 μM peptide concentration, PBS (pH 7.4).

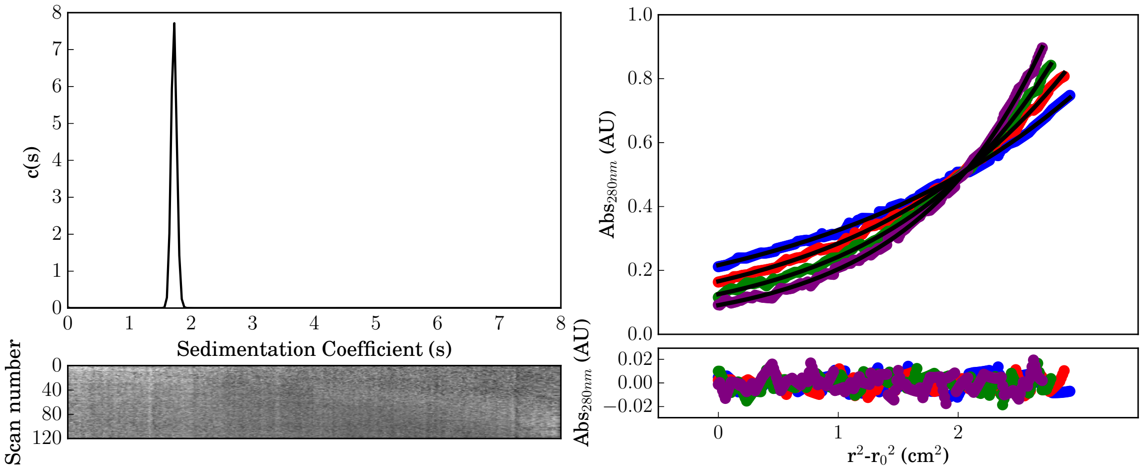

Supplementary Figure 54 CC-Type2-LL-L17Q - AUC data and fits (top) and residuals (bottom) ($\bar{\upsilon}$ = 0.769 cm^3 g^{-1}). Left: continuous c(s) distribution from sedimentation-velocity data at 50k rpm returning s = 1.724 S, s20,w = 2.058 S, f/f0 = 1.251 and mw = 19,589 Da (6.0 × monomer mass) at 95% confidence level. Conditions: 150 μM peptide concentration, PBS (pH 7.4). Residuals for sedimentation-velocity experiments are shown as a bitmap in which the greyscale shade indicates the difference between the fit and raw data. Scans are ordered vertically, with earlier scans at the top. The horizontal axis is the radial range over which the data were fitted. Right: sedimentation-equilibrium data (top, dots) and fitted single-ideal species model curves at 21k (blue), 24k (red), 27k (green) and 30k (purple) rpm. The fit returns a mass of 19,700 Da (6.0 × monomer mass, 95% confidence limits 19,619 – 19,785). Bottom: residuals for the above fits using the same colour scheme as above. Conditions: 70 μM peptide concentration, PBS (pH 7.4).

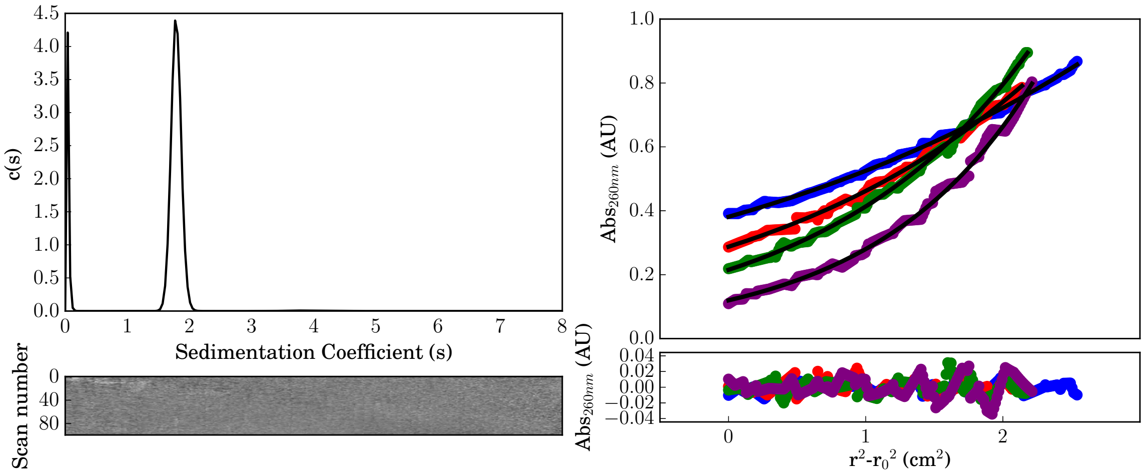

Supplementary Figure 55 CC-Type2-LL-L17E - AUC data and fits (top) and residuals (bottom) ($\bar{\upsilon}$ = 0.768 cm^3 g^{-1}). Left: continuous c(s) distribution from sedimentation-velocity data at 50k rpm returning s = 1.787 S, s20,w = 2.129 S, f/f0 = 1.230 and mw = 20,059 Da (6.1 × monomer mass) at 95% confidence level. Conditions: 150 μM peptide concentration, PBS (pH 7.4). Residuals for sedimentation-velocity experiments are shown as a bitmap in which the greyscale shade indicates the difference between the fit and raw data. Scans are ordered vertically, with earlier scans at the top. The horizontal axis is the radial range over which the data were fitted. Right: sedimentation-equilibrium data (top, dots) and fitted single-ideal species model curves at 18 k (blue), 22 k (red), 26 k (green) and 30 k (purple) rpm. The fit returns a mass of 19,060 Da (5.8 × monomer mass, 95% confidence limits 18,838 – 19,078). Bottom: residuals for the above fits using the same colour scheme as above. Conditions: 150 μM peptide concentration, PBS (pH 7.4).

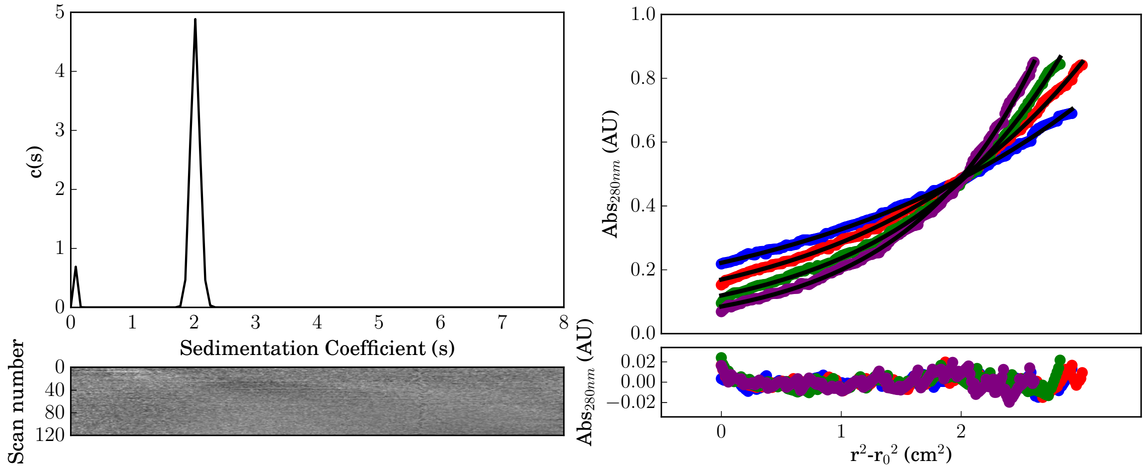

Supplementary Figure 56 CC-Type2-IF - AUC data and fits (top) and residuals (bottom) ($\bar{v}$ = 0.759 cm^3 g^{-1}). Left: continuous c(s) distribution from sedimentation-velocity data at 50k rpm returning s = 2.019 S, s20,w = 2.314 S, f/f0 = 1.101 and mw = 19,136 Da (5.7 × monomer mass) at 95% confidence level. Conditions: 150 μM peptide concentration, PBS (pH 7.4). Residuals for sedimentation-velocity experiments are shown as a bitmap in which the greyscale shade indicates the difference between the fit and raw data. Scans are ordered vertically, with earlier scans at the top. The horizontal axis is the radial range over which the data were fitted. Right: sedimentation-equilibrium data (top, dots) and fitted single-ideal species model curves at 18k (blue), 21k (red), 24k (green) and 27k (purple) rpm. The fit returns a mass of 23,390 Da (6.9 × monomer mass, 95% confidence limits 23,308 – 23,628). Bottom: residuals for the above fits using the same colour scheme as above. Conditions: 70 μM peptide concentration, PBS (pH 7.4).

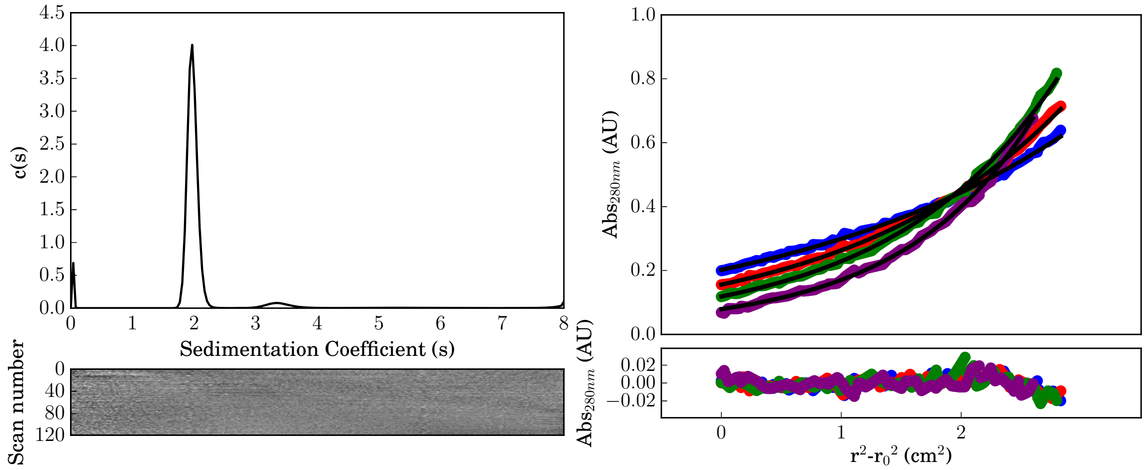

Supplementary Figure 57 CC-Type2-FV - AUC data and fits (top) and residuals (bottom) ($\bar{v}$ = 0.759 cm^3 g^{-1}). Left: continuous c(s) distribution from sedimentation-velocity data at 50k rpm returning s = 1.974 S, s20,w = 2.262 S, f/f0 = 1.134 and mw = 19,362 Da (5.7 × monomer mass) at 95% confidence level. Conditions: 150 μM peptide concentration, PBS (pH 7.4). Residuals for sedimentation-velocity experiments are shown as a bitmap in which the greyscale shade indicates the difference between the fit and raw data. Scans are ordered vertically, with earlier scans at the top. The horizontal axis is the radial range over which the data were fitted. Right: sedimentation-equilibrium data (top, dots) and fitted single-ideal species model curves at 18k (blue), 21k (red), 24k (green) and 27k (purple) rpm. The fit returns a mass of 23550 Da (7.0 × monomer mass, 95% confidence limits 23,377 – 23,719). Bottom: residuals for the above fits using the same colour scheme as above. Conditions: 70 μM peptide concentration, PBS (pH 7.4).

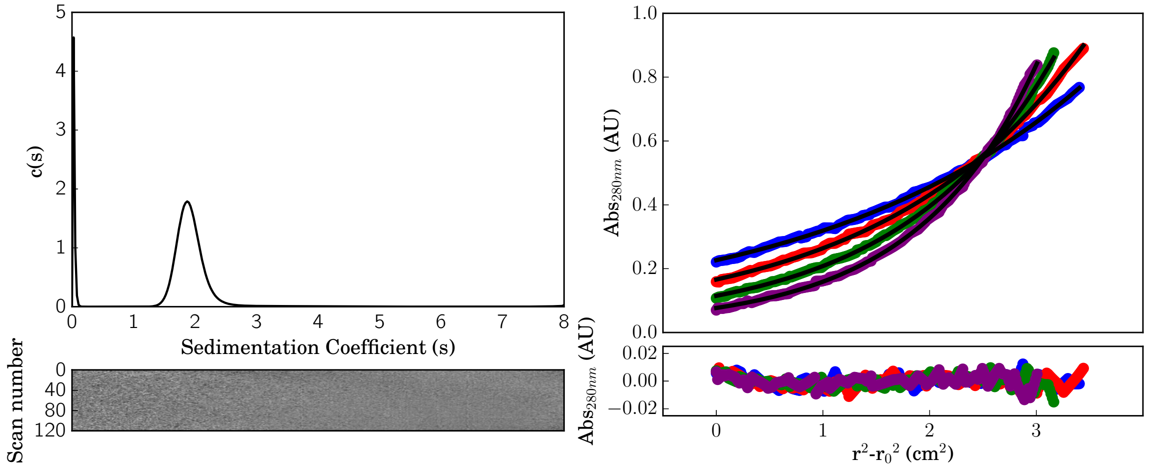

Supplementary Figure 58 CC-Type2-LF - AUC data and fits (top) and residuals (bottom) ($\bar{\upsilon}$ = 0.759 cm^3 g^{-1}). Left: continuous c(s) distribution from sedimentation-velocity data at 50k rpm returning s = 1.906 S, s20,w = 2.183 S, f/f0 = 1.184 and mw = 19,581 Da (5.8 × monomer mass) at 95% confidence level. Conditions: 150 μM peptide concentration, PBS (pH 7.4). Residuals for sedimentation-velocity experiments are shown as a bitmap in which the greyscale shade indicates the difference between the fit and raw data. Scans are ordered vertically, with earlier scans at the top. The horizontal axis is the radial range over which the data were fitted. Right: sedimentation-equilibrium data (top, dots) and fitted single-ideal species model curves at 18k (blue), 21k (red), 24k (green) and 27k (purple) rpm. The fit returns a mass of 22,700 Da (6.7 × monomer mass, 95% confidence limits 22,590 – 22,813). Bottom: residuals for the above fits using the same colour scheme as above. Conditions: 70 μM peptide concentration, PBS (pH 7.4).

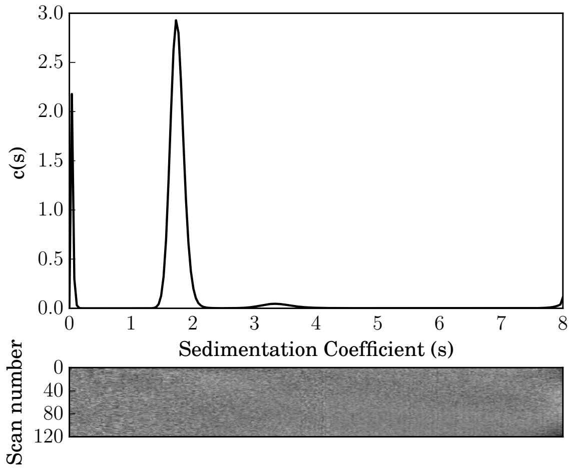

Supplementary Figure 59 CC-Type2-FF - AUC data and fits (top) and residuals (bottom) ($\bar{\upsilon}$ = 0.742 cm^3 g^{-1}). continuous c(s) distribution from sedimentation-velocity data at 50k rpm returning s = 1.748 S, s20,w = 1.871 S, f/f0 = 1.157 and mw = 14,833 Da (4.2 × monomer mass) at 95% confidence level. Conditions: 150 μM peptide concentration, PBS (pH 7.4). Residuals for sedimentation-velocity experiments are shown as a bitmap in which the greyscale shade indicates the difference between the fit and raw data. Scans are ordered vertically, with earlier scans at the top. The horizontal axis is the radial range over which the data were fitted.

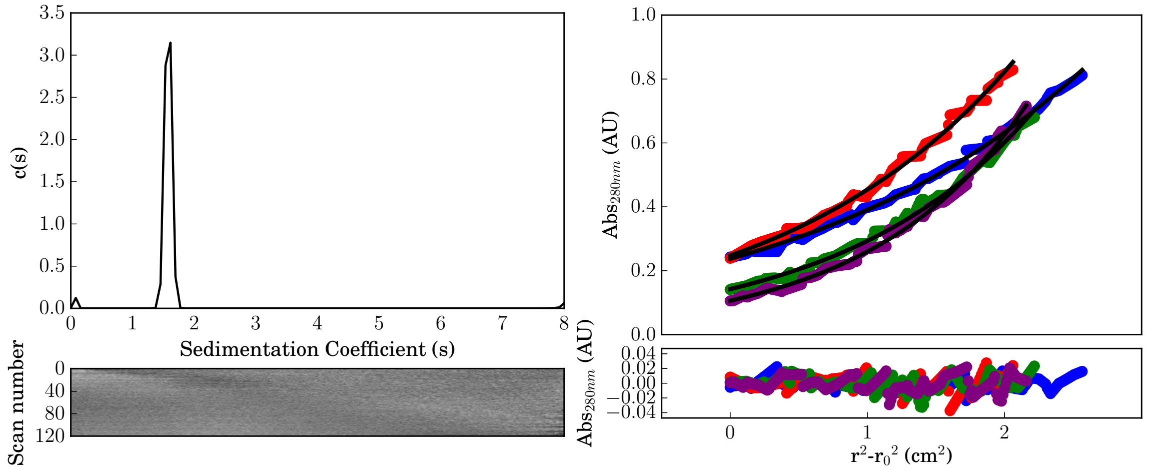

Supplementary Figure 60 5H2L_2.1-I9L - AUC data and fits (top) and residuals (bottom) ($\bar{v}$ = 0.781 cm^3 g^{-1}). Left: continuous c(s) distribution from sedimentation-velocity data at 50k rpm returning s = 1.579 S, s20,w = 1.997 S, f/f0 = 1.198 and mw = 17,675 Da (4.3 × monomer mass) at 95% confidence level. Conditions: 150 μM peptide concentration, PBS (pH 7.4). Residuals for sedimentation-velocity experiments are shown as a bitmap in which the greyscale shade indicates the difference between the fit and raw data. Scans are ordered vertically, with earlier scans at the top. The horizontal axis is the radial range over which the data were fitted. Right: sedimentation-equilibrium data (top, dots) and fitted single-ideal species model curves at 24k (blue), 27k (red), 30k (green) and 33k (purple) rpm. The fit returns a mass of 16,490 Da (4.0 × monomer mass, 95% confidence limits 16,354 – 16,622). Bottom: residuals for the above fits using the same colour scheme as above. Conditions: 70 μM peptide concentration, PBS (pH 7.4).

Hydrophobic-dye binding assays

β-Branched at a and d positions

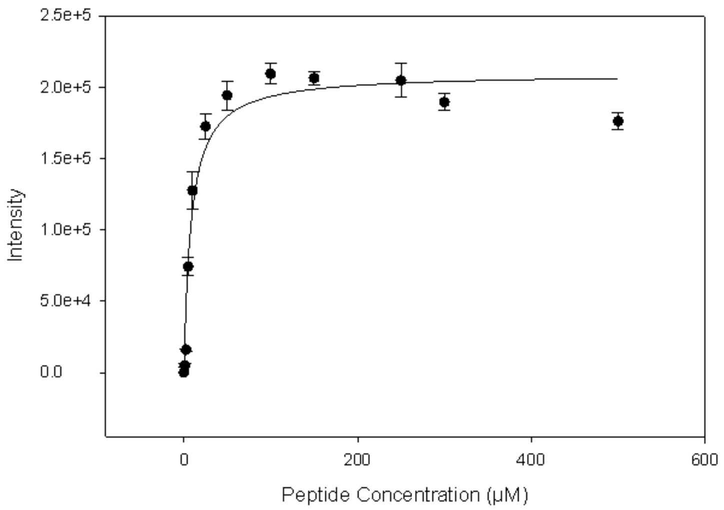

Supplementary Figure 61 CC-Type2-IV - Fluorescence DPH binding data fitted to a one-site saturation curve. Intensity values displayed are normalised to blank readings. K_d to peptide = 8.3 μM (standard error +/− 2.0 μM).

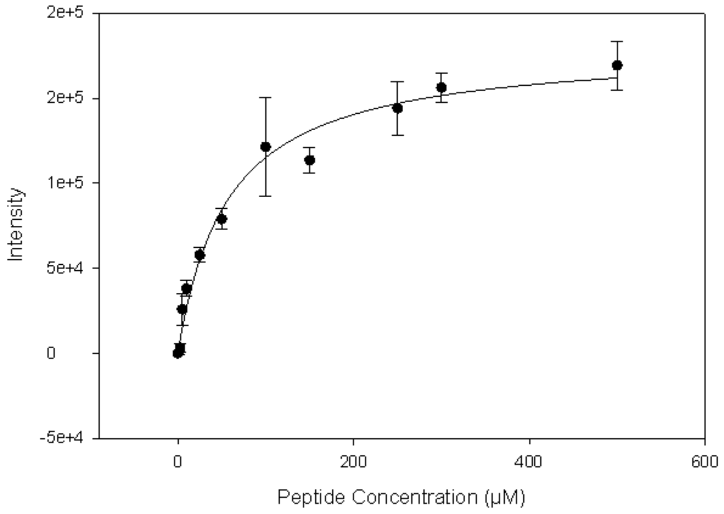

Supplementary Figure 62 CC-Type2-II - Fluorescence DPH binding data fitted to a one-site saturation curve. Intensity values displayed are normalised to blank readings. K_d to peptide = 56.5 μM (standard error +/− 9.5 μM).

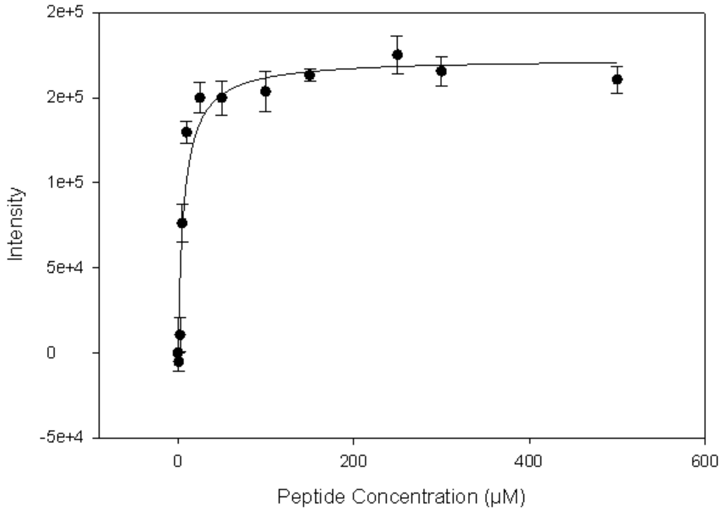

Supplementary Figure 63 CC-Type2-VI - Fluorescence DPH binding data fitted to a one-site saturation curve. Intensity values displayed are normalised to blank readings. K_d to peptide = 7.0 μM (standard error +/− 1.9 μM).

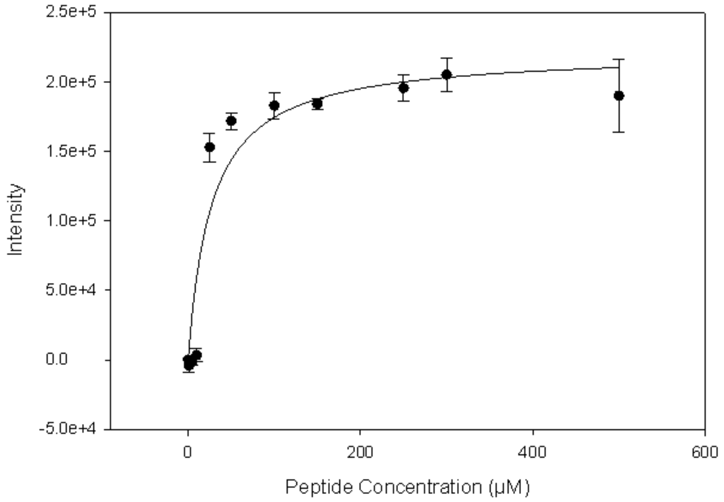

Supplementary Figure 64 CC-Type2-VV - Fluorescence DPH binding data fitted to a one-site saturation curve. Intensity values displayed are normalised to blank readings. K_d to peptide = 27.0 μM (standard error +/− 10.9 μM).

β-Branched only at *d* positions

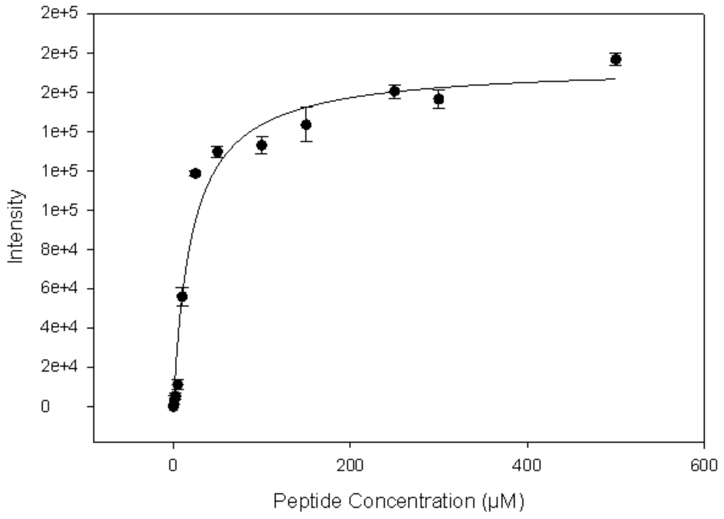

Supplementary Figure 65 CC-Type2-LI - Fluorescence DPH binding data fitted to a one-site saturation curve. Intensity values displayed are normalised to blank readings. K_d to peptide = 20.9 μM (standard error $+/-$ 3.0 μM).

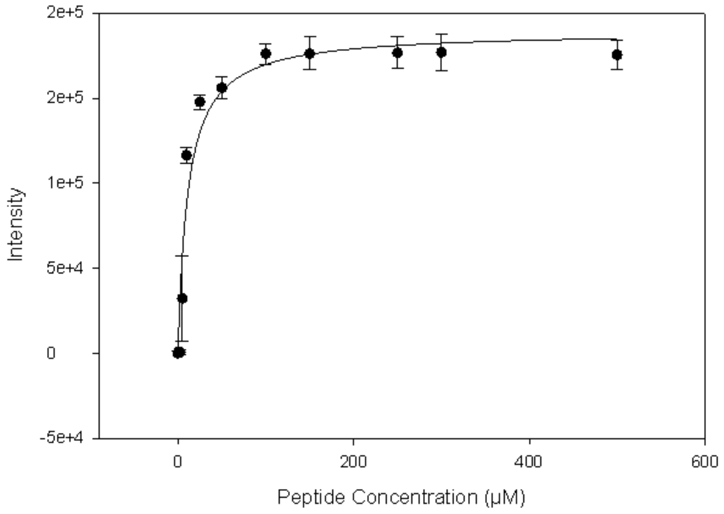

Supplementary Figure 66 CC-Type2-deLI - Fluorescence DPH binding data fitted to a one-site saturation curve. Intensity values displayed are normalised to blank readings. K_d to peptide = 11.5 μM (standard error $+/-$ 3.0 μM).

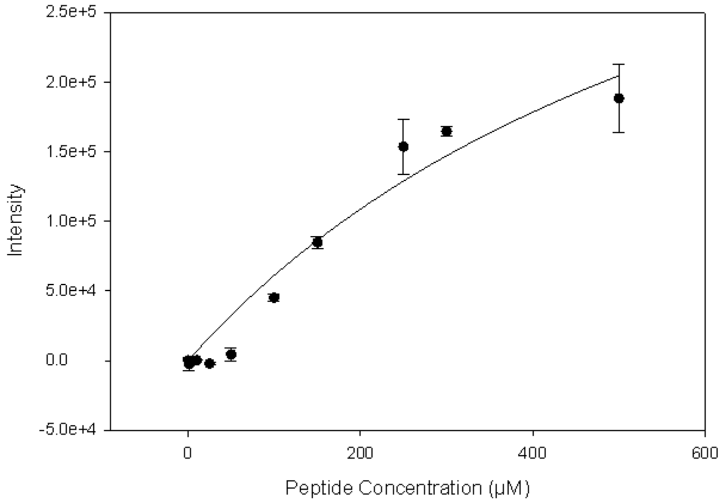

Supplementary Figure 67 CC-Type2-LV - Fluorescence DPH binding data fitted to a one-site saturation curve. Intensity values displayed are normalised to blank readings. K_d to peptide not determined due to not reaching saturation.

***β*-Branched only at *a* positions**

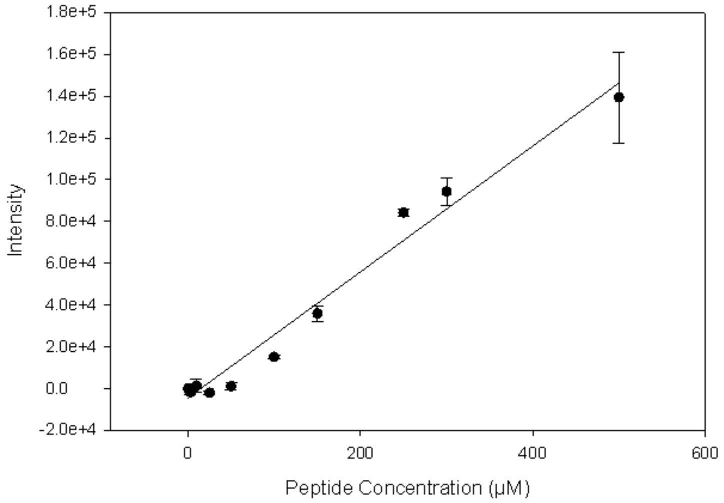

Supplementary Figure 68 CC-Type2-VL - Fluorescence DPH binding data fitted to a linear polynomial. Intensity values displayed are normalised to blank readings.

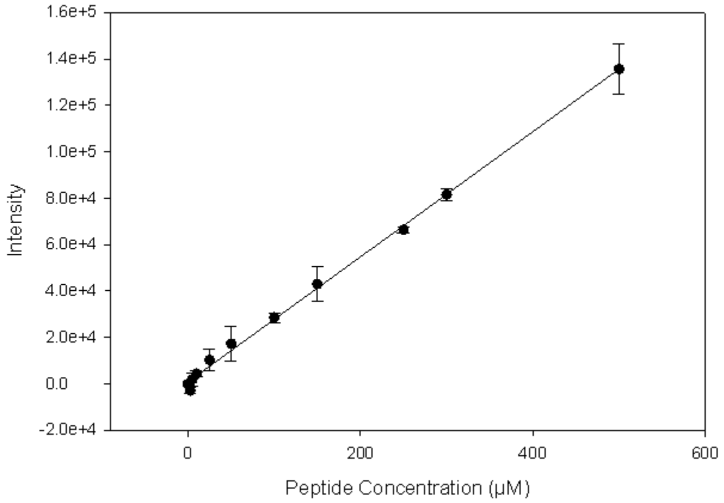

Supplementary Figure 69 CC-Type2-IL-Sg - Fluorescence DPH binding data fitted to a linear polynomial. Intensity values displayed are normalised to blank readings.

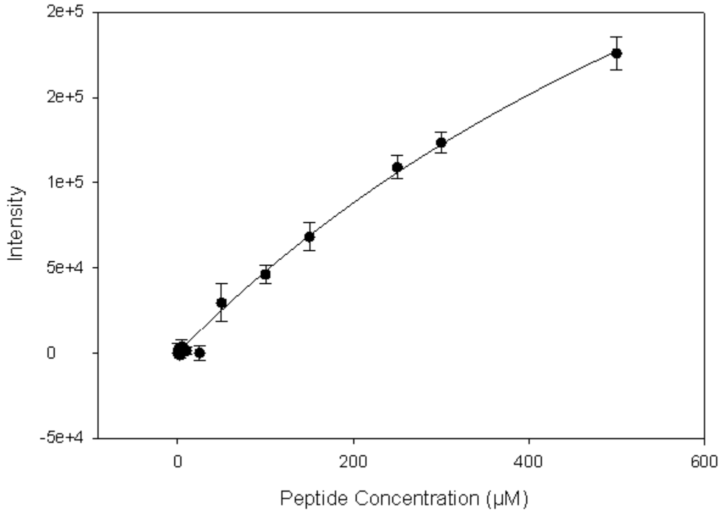

Supplementary Figure 70 CC-Type2-IL-Sg-L17E - Fluorescence DPH binding data fitted to a one-site saturation curve. Intensity values displayed are normalised to blank readings. K_d to peptide not determined due to not reaching saturation.

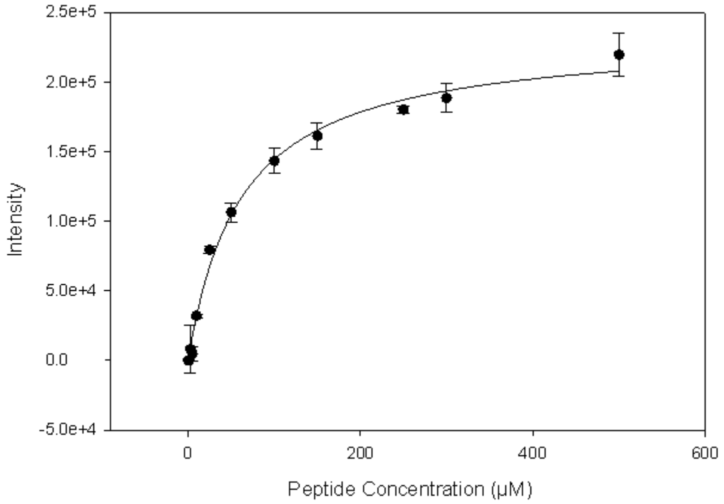

Supplementary Figure 71 CC-Type2-IL-Sg-L17E - Fluorescence TMA-DPH binding data fitted to a one-site saturation curve. Intensity values displayed are normalised to blank readings. K_d to peptide = 61.6 μM (standard error +/− 7.2 μM).

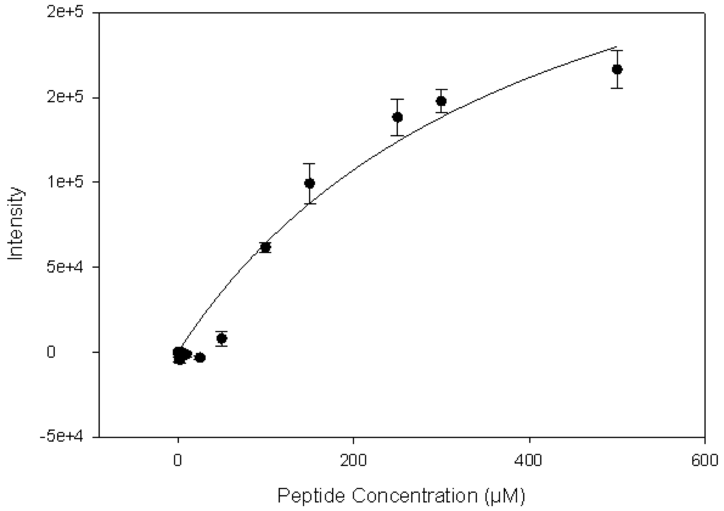

Supplementary Figure 72 CC-Type2-IL-Eg - Fluorescence DPH binding data fitted to a one-site saturation curve. Intensity values displayed are normalised to blank readings. K_d to peptide not determined due to abnormal behaviour of peptide at low concentrations.

Leucine in *a* and *d* positions

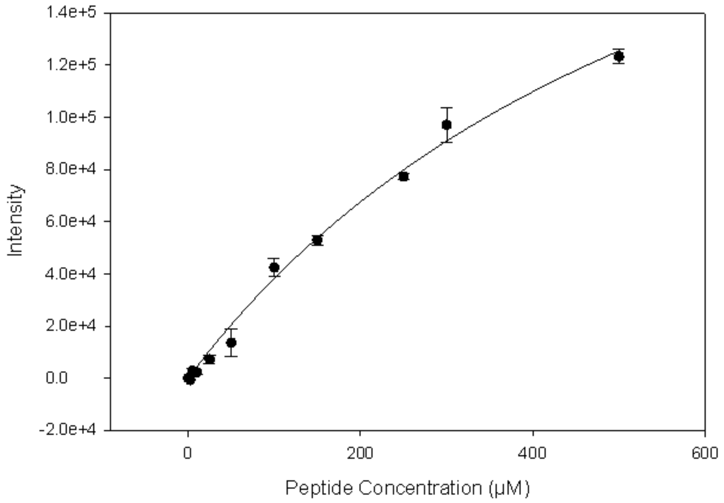

Supplementary Figure 73 CC-Type2-LL - Fluorescence DPH binding data fitted to a one-site saturation curve. Intensity values displayed are normalised to blank readings. K_d to peptide not determined due to not reaching saturation.

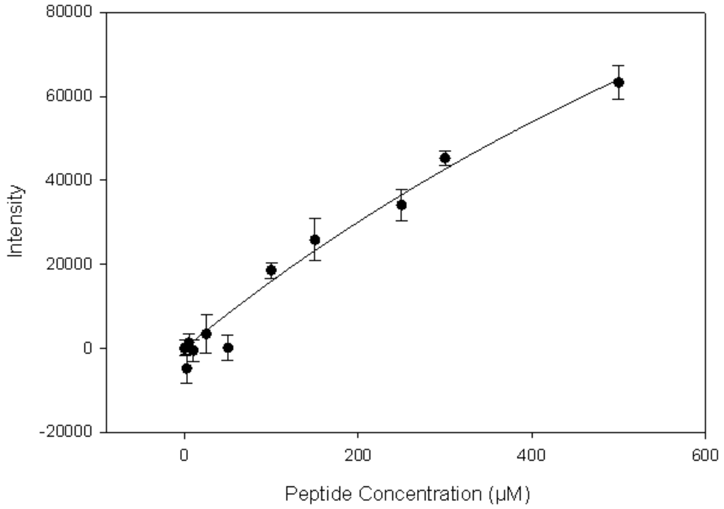

Supplementary Figure 74 CC-Type2-LL-Sg - Fluorescence DPH binding data fitted to a one-site saturation curve. Intensity values displayed are normalised to blank readings. K_d to peptide not determined due to not reaching saturation.

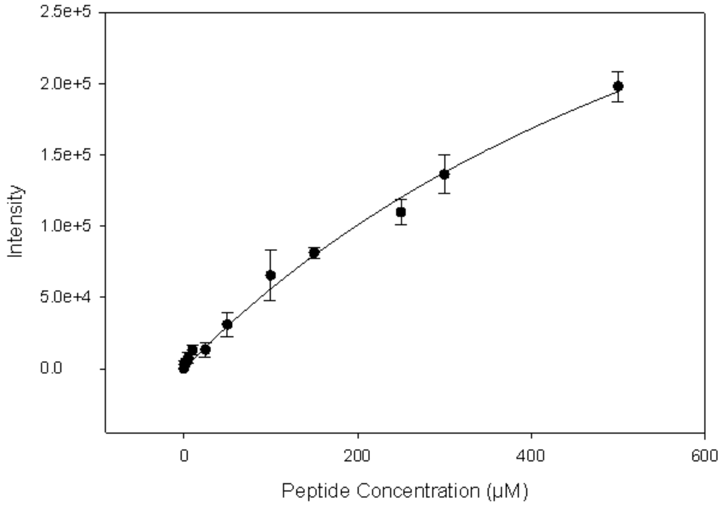

Supplementary Figure 75 CC-Type2-LL-L17Q - Fluorescence DPH binding data fitted to a one-site saturation curve. Intensity values displayed are normalised to blank readings. K_d to peptide not determined due to not reaching saturation.

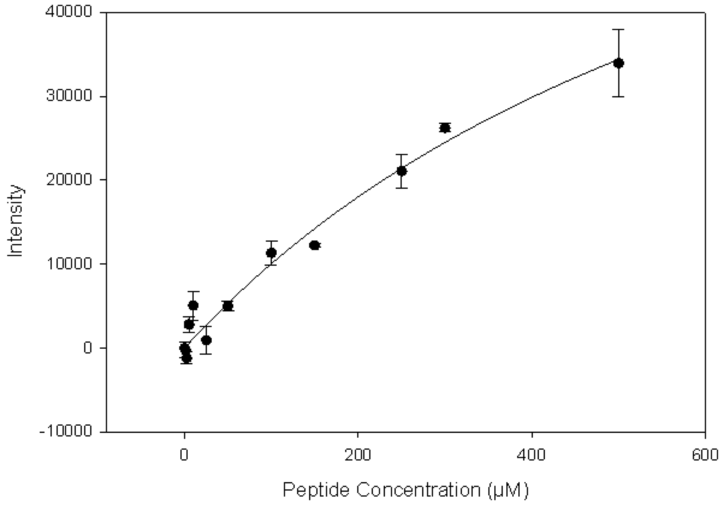

Supplementary Figure 76 CC-Type2-LL-L17E - Fluorescence DPH binding data fitted to a one-site saturation curve. Intensity values displayed are normalised to blank readings. K_d to peptide not determined due to not reaching saturation.

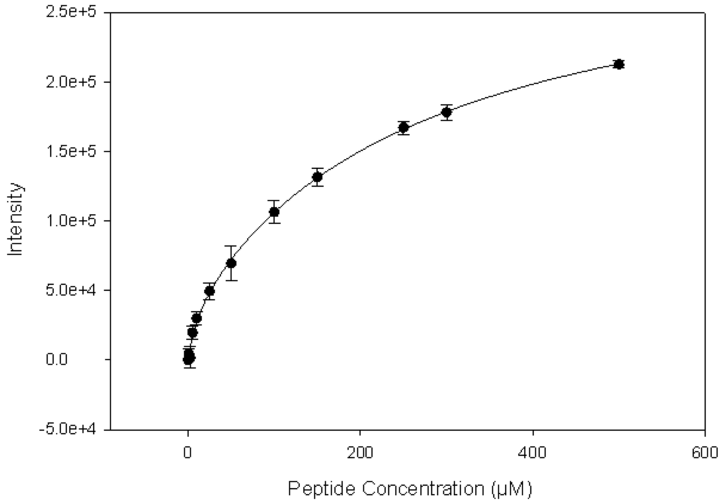

Supplementary Figure 77 CC-Type2-LL-L17E - Fluorescence TMA-DPH binding data fitted to a two site saturation curve. Intensity values displayed are normalised to blank readings. K_d to peptide = 10.3 μM and 272.4 μM (standard error +/− 6.9 μM and +/− 64.9 μM respectively).

Sequences containing phenylalanine residues

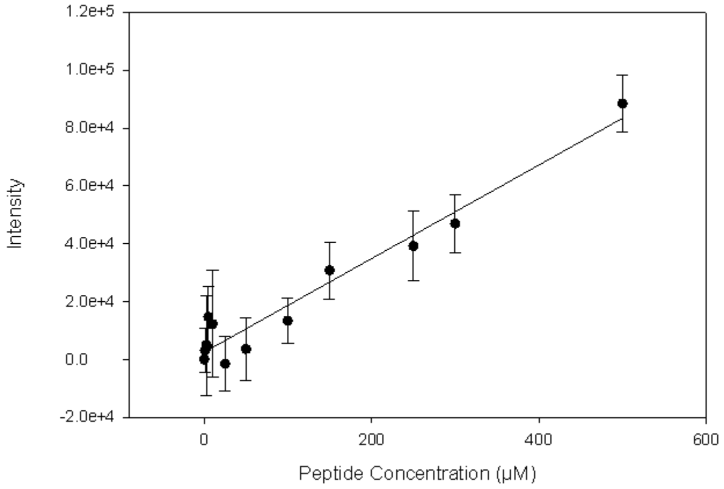

Supplementary Figure 78 CC-Type2-IF - Fluorescence DPH binding data fitted to a linear polynomial. Intensity values displayed are normalised to blank readings.

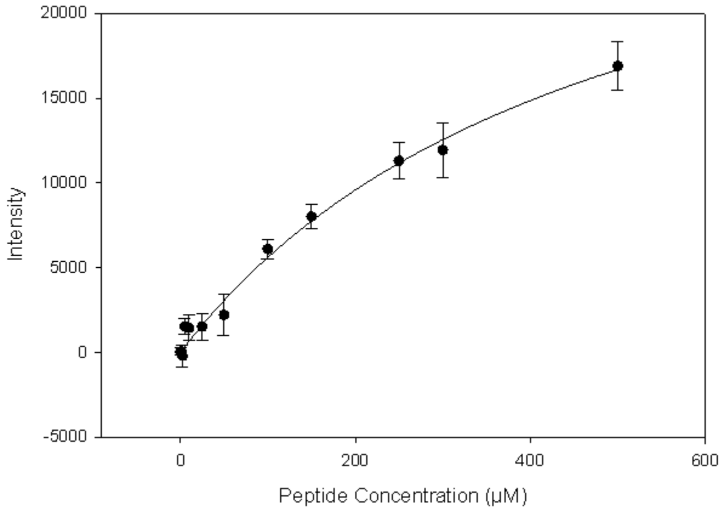

Supplementary Figure 79 CC-Type2-LF - Fluorescence DPH binding data fitted to a one-site saturation curve. Intensity values displayed are normalised to blank readings. K_d to peptide not determined due to not reaching saturation.

Crystal structures and analysis

	Crystallisation conditions	pH
CC-Type2-IV	50 mM HEPES and 8% w/v PEG 8000	7.0
CC-Type2-II	100 mM sodium citrate tribasic dihydrate, 50 mM sodium cacodylate and 15% v/v 2-propanol	6.5
CC-Type2-VV	5 mM manganese(II) chloride tetrahydrate, 50 mM sodium citrate and 1.25 M 1,6-hexanediol	5.6
CC-Type2-deLI	100 mM magnesium chloride hexahydrate, 50 mM sodium HEPES and 15 % w/v PEG 400	7.5
CC-Type2-IL-Sg	100 mM ammonium sulfate and 15% w/v PEG 8000	-
CC-Type2-IL-Sg-L17E	100 mM ammonium phosphate monobasic, 50 mM Tris and 25% v/v MPD	8.5
CC-Type2-LL	10 mM sodium/potassium phosphate, 50 mM Bis-Tris propane and 10% w/v PEG 3350	8.5
CC-Type2-LL-Sg	100 mM potassium formate and 10% w/v PEG 3350	-
CC-Type2-LL-L17Q	50 mM SPG buffer (succinic acid, sodium phosphate monobasic monohydrate and glycine) and 12.5% w/v PEG 1500	9.0
CC-Type2-LL-L17E	50 mM SPG buffer (succinic acid, sodium phosphate monobasic monohydrate and glycine) and 12.5% w/v PEG 1500	8.0
5H2L_2-I8L	20 mM potassium phosphate monobasic , 8% w/v PEG 8000 and 10% w/v glycerol	-
CC-Type2-FI	50 mM Tris and 10% w/v PEG 6000	8.5
CC-Type2-LF	50 mM Tris and 10% w/v PEG 6000	8.5

Supplementary Table 2 Crystallisation conditions for each crystal. Conditions shown are final conditions from half dilution with peptide stock.

	CC-Type2-IV	CC-Type2-II	CC-Type2-VV
Wavelength (Å)	0.9795	0.9795	0.9282
Resolution range (Å)	45.31 - 1.6 (1.657 - 1.6)	34.02 - 1.77 (1.833 - 1.77)	13.87 - 1.15 (1.191 - 1.15)
Space group	P 2 21 21	I 4 2 2	C 1 2 1
Unit cell lengths (Å)	38.25 48.34 129.98	48.11 48.11 126.97	65.56 96.79 56.40
Unit cell angles (°)	90 90 90	90 90 90	90 124.02 90
Total reflections	349721 (36014)	154569 (15080)	622871 (63196)
Unique reflections	32376 (3126)	7673 (742)	99212 (9992)
Multiplicity	10.8 (11.5)	20.1 (20.3)	6.3 (6.3)
Completeness (%)	99 (98)	100 (100)	96 (97)
Mean $I/\sigma I$	6.61 (2.09)	17.90 (3.84)	13.54 (5.11)
Wilson B-factor (Å^2)	20.4	27	7.34
R_{merge}	0.222 (0.858)	0.089 (0.814)	0.064 (0.293)
R_{meas}	0.234 (0.898)	0.091 (0.835)	0.070 (0.320)
CC1/2 (%)	98.2 (93.4)	99.9 (93.5)	99.7 (98.6)
CC* (%)	99.5 (98.3)	100 (98.3)	99.9 (99.7)
Reflections used in refinement	32334 (3122)	7660 (741)	98669 (9845)
Reflections used for R-free	1609 (151)	377 (37)	5031 (550)
R_{work}	0.206 (0.219)	0.207 (0.291)	0.176 (0.195)
R_{free}	0.205 (0.235)	0.243 (0.261)	0.189 (0.215)
CC_{work} (%)	93.4 (89.7)	94.3 (90.7)	95.3 (96.0)
CC_{free} (%)	95.2 (86.0)	93.9 (85.7)	93.8 (94.1)
Number of non-hydrogen atoms	1856	610	3193
macromolecules	1618	527	2795
ligands	35	14	48
Protein residues	219	64	384
RMS(bonds) (Å)	0.007	0.008	0.007
RMS(angles) (°)	0.86	0.97	0.73
Ramachandran favored (%)	100	100	100
Ramachandran allowed (%)	0	0	0
Ramachandran outliers (%)	0	0	0
Rotamer outliers (%)	1.3	0	1.1
Clashscore	6.24	3.47	2.27
Average B-factor (Å^2)	28.6	30.67	13.76
macromolecules (Å^2)	26.48	28.53	11.7
ligands (Å^2)	45.35	52	14.04
solvent (Å^2)	42.63	42.64	30.18
Number of TLS groups	7		12

	CC-Type2-deLI	CC-Type2-IL-Sg	CC-Type2-IL-Sg-L17E
Wavelength (Å)	0.9795	0.9282	0.9795
Resolution range (Å)	44.96 - 1.26 (1.305 - 1.26)	58.36 - 1.95 (2.02 - 1.95)	56.26 - 2.2 (2.279 - 2.2)
Space group	P 2 21 21	P 2 21 21	R 3 :H
Unit cell lengths (Å)	38.20 47.95 129.34	58.36 90.03 112.47	66.77 66.77 243.20
Unit cell angles (°)	90 90 90	90 90 90	90 90 120
Total reflections	746484 (67167)	530552 (52103)	189814 (19136)
Unique reflections	65144 (6398)	43942 (4324)	20520 (2051)
Multiplicity	11.5 (10.5)	12.1 (12.0)	9.3 (9.3)
Completeness (%)	100 (100)	100 (100)	100 (100)
Mean $I/\sigma I$	15.52 (2.06)	11.67 (2.10)	16.38 (2.01)
Wilson B-factor ($Å^2$)	16.44	30.72	52.92
R_{merge}	0.064 (1.086)	0.153 (1.924)	0.061 (1.053)
R_{meas}	0.067 (1.142)	0.159 (2.008)	0.064 (1.113)
CC1/2 (%)	99.7 (81.9)	99.9 (39.8)	99.9 (58.6)
CC* (%)	99.9 (94.9)	100 (75.4)	100 (86)
Reflections used in refinement	64921 (6393)	43919 (4322)	20500 (2051)
Reflections used for R-free	3262 (299)	2119 (203)	1103 (115)
R_{work}	0.192 (0.384)	0.179 (0.272)	0.202 (0.296)
R_{free}	0.213 (0.360)	0.210 (0.308)	0.263 (0.340)
CC_{work} (%)	95.7 (85.7)	97.0 (76.0)	95.6 (72.7)
CC_{free} (%)	96.7 (84.9)	95.1 (66.1)	95.9 (52.4)
Number of non-hydrogen atoms	2136	4575	3512
macromolecules	1547	4304	3442
ligands	418	40	55
Protein residues	220	548	419
RMS(bonds) (Å)	0.042	0.008	0.027
RMS(angles) (°)	3.01	0.84	2.62
Ramachandran favored (%)	100	100	98
Ramachandran allowed (%)	0	0.18	0.71
Ramachandran outliers (%)	0	0	0.95
Rotamer outliers (%)	0	1.7	8.7
Clashscore	4.12	2.46	16.66
Average B-factor ($Å^2$)	24.29	41.32	72.36
macromolecules ($Å^2$)	22.97	40.86	71.96
ligands ($Å^2$)	22.29	57.75	95.32
solvent ($Å^2$)	41.12	47.13	79.97
Number of TLS groups		18	14

	CC-Type2-LL	CC-Type2-LL-Sg	CC-Type2-LL-L17Q
Wavelength (Å)	0.9795	0.9795	0.9282
Resolution range (Å)	73.21 - 2.701 (2.798 - 2.701)	60.14 - 2.051 (2.124 - 2.051)	74.1 - 2.3 (2.383 - 2.3)
Space group	P 41 3 2	P 41 3 2	P 41 3 2
Unit cell lengths (Å)	103.53 103.53 103.53	104.16 104.16 104.16	104.79 104.79 104.79
Unit cell angles (°)	90 90 90	90 90 90	90 90 90
Total reflections	364604 (36968)	888358 (87460)	606720 (50433)
Unique reflections	5617 (550)	12657 (1224)	9242 (899)
Multiplicity	64.9 (67.2)	70.2 (71.2)	65.6 (56.1)
Completeness (%)	100 (100)	100 (100)	100 (100)
Mean $I/\sigma I$	21.25 (4.51)	25.24 (1.77)	38.13 (4.98)
Wilson B-factor (Å^2)	62.4	50.44	53.46
R_{merge}	0.205 (1.457)	0.152 (4.746)	0.082 (1.243)
R_{meas}	0.207 (1.468)	0.153 (4.78)	0.083 (1.254)
CC1/2 (%)	100 (60)	99.9 (44.1)	100 (87.8)
CC* (%)	100 (86.6)	100 (78.3)	100 (96.7)
Reflections used in refinement	5616 (550)	12653 (1228)	9242 (899)
Reflections used for R-free	279 (23)	601 (54)	453 (36)
R_{work}	0.219 (0.299)	0.206 (0.367)	0.220 (0.286)
R_{free}	0.267 (0.320)	0.240 (0.344)	0.254 (0.349)
CC_{work} (%)	96.9 (66.2)	96.5 (55.9)	98.2 (77.8)
CC_{free} (%)	80.2 (39.4)	95.5 (57.3)	94.9 (69.9)
Number of non-hydrogen atoms	680	766	690
macromolecules	671	737	676
ligands		12	
Protein residues	89	92	88
RMS(bonds) (Å)	0.012	0.073	0.01
RMS(angles) (°)	1.62	5.23	1.31
Ramachandran favored (%)	98.8	98.9	97.6
Ramachandran allowed (%)	1.2	1.1	1.2
Ramachandran outliers (%)	0	0	1.2
Rotamer outliers (%)	3.3	4.9	3.3
Clashscore	4.85	3.29	4
Average B-factor (Å^2)	65.32	67.3	61.42
macromolecules (Å^2)	65.35	66.52	61.31
ligands (Å^2)		116.52	
solvent (Å^2)	62.84	66.61	66.65
Number of TLS groups		3	

	CC-Type2-LL-L17E	5H2L 2.1-I9L	CC-Type2-FI
Wavelength (Å)	0.9795	0.9282	0.9762
Resolution range (Å)	58.1 - 1.55 (1.605 - 1.55)	41.68 - 1.7 (1.761 - 1.7)	48.77 - 1.7 (1.761 - 1.7)
Space group	P 1 21 1	P 21 21 21	P 1 21 1
Unit cell lengths (Å)	45.32 117.94 59.24	34.29 65.32 83.37	30.66 85.00 59.61
Unit cell angles (°)	90 101.24 90	90 90 90	90 92.49 90
Total reflections	525762 (53347)	219161 (19909)	166925 (15619)
Unique reflections	88192 (8800)	21242 (2066)	33453 (3299)
Multiplicity	6.0 (6.1)	10.3 (9.6)	5.0 (4.7)
Completeness (%)	100 (100)	99 (99)	100 (100)
Mean $I/\sigma I$	11.87 (2.29)	14.29 (4.00)	6.41 (2.12)
Wilson B-factor (Å^2)	18.99	20.51	18.71
R_{merge}	0.071 (0.768)	0.106 (1.088)	0.142 (0.902)
R_{meas}	0.077 (0.840)	0.112 (1.156)	0.158 (1.013)
CC1/2 (%)	99.8 (82.8)	99.8 (76.9)	99.2 (70.6)
CC* (%)	100 (95.2)	100 (93.3)	99.8 (91.0)
Reflections used in refinement	88138 (8792)	21203 (2051)	33430 (3294)
Reflections used for R-free	4473 (475)	1025 (96)	1670 (173)
R_{work}	0.187 (0.259)	0.183 (0.213)	0.176 (0.263)
R_{free}	0.229 (0.305)	0.232 (0.254)	0.222 (0.309)
CC_{work} (%)	96.9 (89.6)	96.6 (92.1)	97.4 (89.4)
CC_{free} (%)	94.8 (82.0)	94.6 (92.4)	96.0 (82.7)
Number of non-hydrogen atoms	6029	1614	3185
macromolecules	5669	1464	2879
ligands	54	19	37
Protein residues	737	174	375
RMS(bonds) (Å)	0.016	0.006	0.006
RMS(angles) (°)	1.57	0.77	0.67
Ramachandran favored (%)	100	100	100
Ramachandran allowed (%)	0	0	0
Ramachandran outliers (%)	0	0	0
Rotamer outliers (%)	0.97	0.58	1.2
Clashscore	4.67	3.99	3.6
Average B-factor (Å^2)	29.08	27.72	24.83
macromolecules (Å^2)	28.55	26.43	23.7
ligands (Å^2)	35.98	31.73	32.8
solvent (Å^2)	37.75	41.63	35.76
Number of TLS groups	24	5	

	CC-Type2-LF
Wavelength (Å)	0.9795
Resolution range (Å)	44.45 - 1.7 (1.761 - 1.7)
Space group	P 1 21 1
Unit cell lengths (Å)	42.00 58.99 47.05
Unit cell angles (°)	90 109.13 90
Total reflections	125623 (5039)
Unique reflections	22169 (1418)
Multiplicity	5.7 (3.6)
Completeness (%)	92 (59)
Mean $I/\sigma I$	36.74 (11.91)
Wilson B-factor ($Å^2$)	15.4
R_{merge}	0.028 (0.078)
R_{meas}	0.031 (0.091)
CC1/2 (%)	99.9 (99.1)
CC* (%)	100 (99.8)
Reflections used in refinement	22164 (1418)
Reflections used for R-free	1085 (79)
R_{work}	0.156 (0.189)
R_{free}	0.184 (0.308)
CC_{work} (%)	97.5 (93.7)
CC_{free} (%)	96.5 (81.5)
Number of non-hydrogen atoms	2234
macromolecules	1962
ligands	35
Protein residues	248
RMS(bonds) (Å)	0.006
RMS(angles) (°)	0.75
Ramachandran favored (%)	99.6
Ramachandran allowed (%)	0
Ramachandran outliers (%)	0.4
Rotamer outliers (%)	2.9
Clashscore	2.3
Average B-factor ($Å^2$)	18.51
macromolecules ($Å^2$)	16.74
ligands ($Å^2$)	35.43
solvent ($Å^2$)	30.69

Supplementary Table 3 Crystallisation data collection and refinement statistics. Highest-resolution shell shown in parentheses. R_{free} represents the R-factor calculated from 5% of reflections that were not used during refinement.

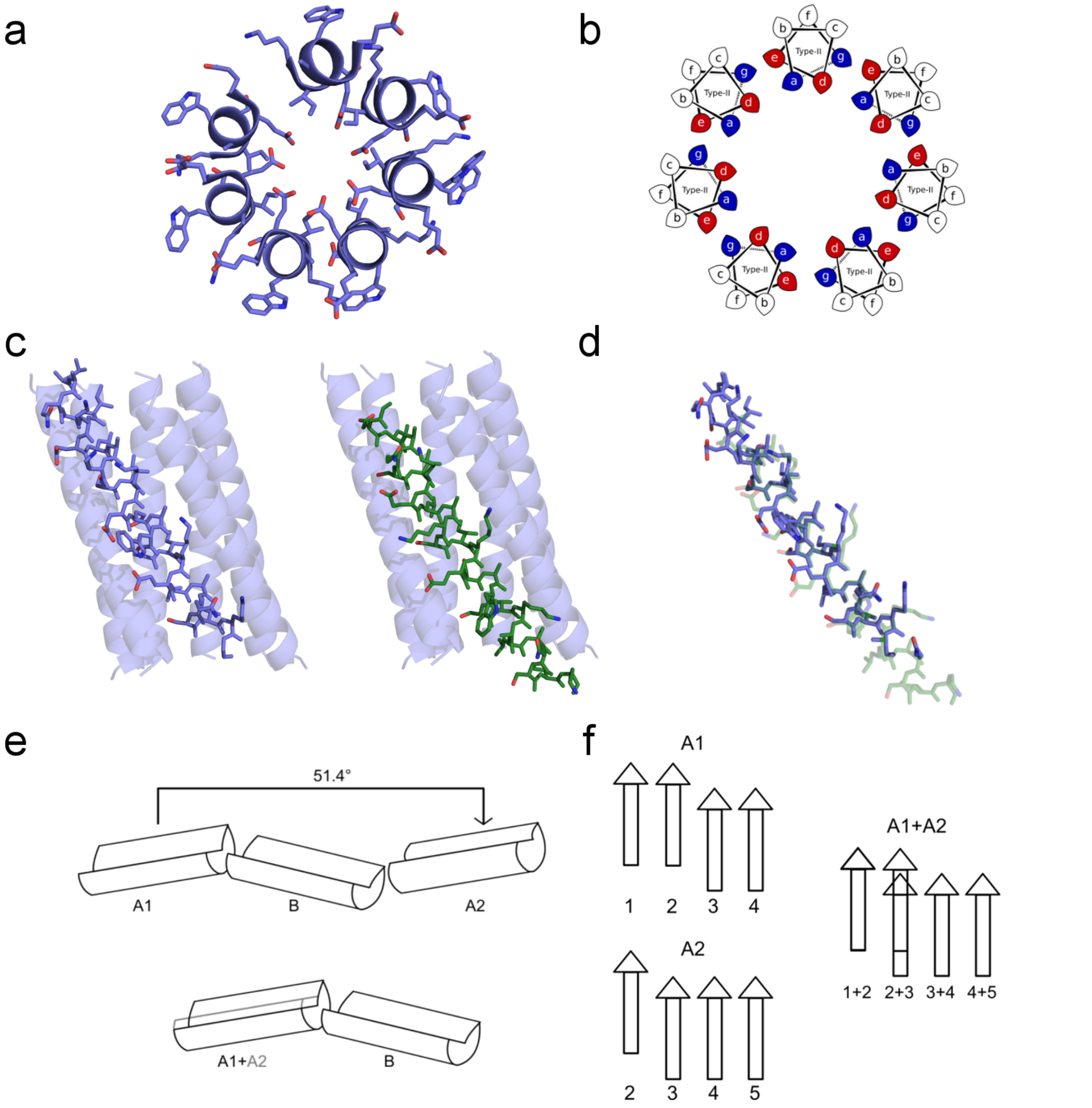

Supplementary Figure 80 X-ray structure of CC-Type2-IL-Sg-L17E and schematic showing the dual-confirmation of a chain in the asymmetric unit. (a) CC-Type2-IL-Sg-L17E viewed from the N termini and a (b) helical wheel representation. (c) The two conformations (purple and green) observed in the electron density map for one of the chains in a biological unit (d) and an image showing their superimposition. (e & f) Diagram showing X-ray crystal structure packing observed in the H3 space-group crystal-form of CC-Type2-IL-Sg-L17E. (a) Two distinct barrels in which barrel A is present in two different conformations, A1 and A2, believed to be stochastically distributed throughout the crystal. Superimposed A1 and A2 barrels that pack at their N termini to barrel B's C termini observed in the asymmetric unit of the crystal structure. (f) Two-dimensional arrow representation of four α-helices nearest to the heptad slip. Superimposition of chains from the A1 and A2 conformations lead to averaging of density where only 2+3 is affected, leading to half occupancy of the terminal residues.

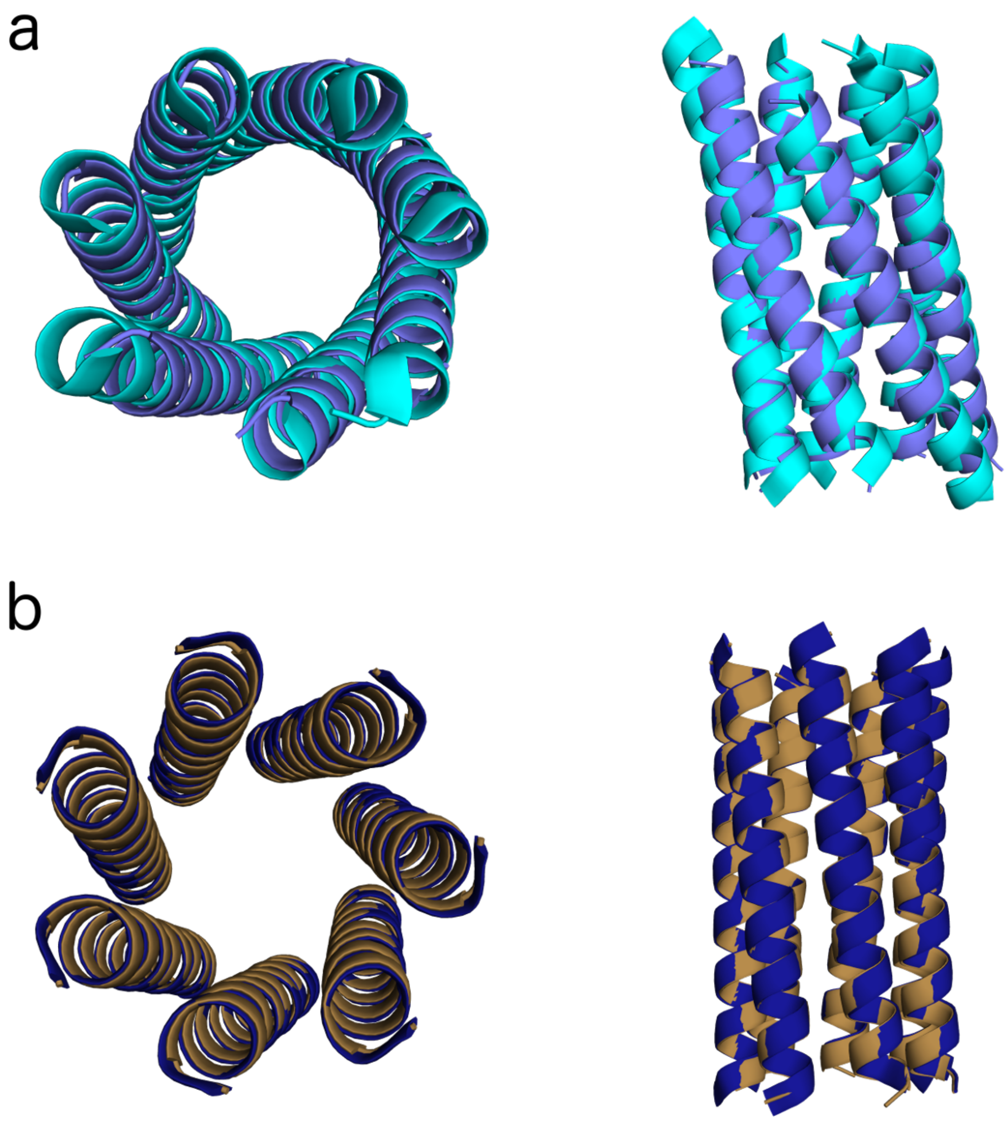

Supplementary Figure 81 Structure alignment of X-ray crystal structures discussed in the manuscript. (a) CC-Type2-IL-Sg (purple) aligned to GCN4-pAA (cyan, accession code: 2HY6) viewed from the N termini (left) and viewed from the side (right). The route-mean-squared deviation for the backbone atoms (564 to 564 atoms) was 0.761 Å. (b) CC-Type2-deLI (brown) aligned to CC-Type2-LI (purple, accession code: 4PNA) viewed from the N termini (left) and viewed from the side (right). The route-mean-squared deviation for the backbone atoms (746 to 746 atoms) was 0.271 Å. Structures were aligned using the Super function in PyMOL and images were created using PyMOL.

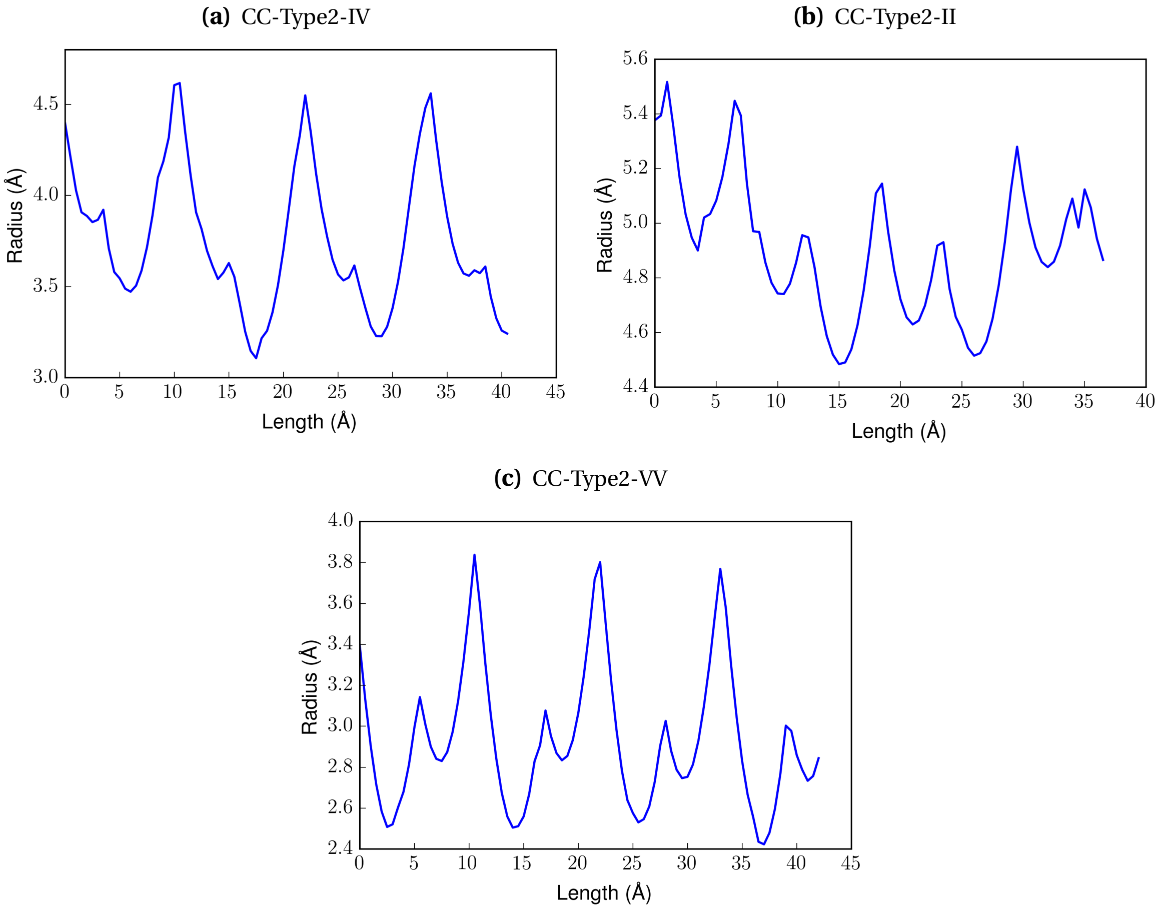

Supplementary Figure 82 Measured radius along the channel of $\beta\beta$-class α-helical barrels. Channel radii were measured with the PyMol plugin Caver 3.0.1[2] on (a) CC-Type2-IV (b) CC-Type2-II and (c) CC-Type2-VV.

CC-Type2-LL iSOCKET Knobs-into-holes interactions

Chain	Residue	Amino Acid	Crick Angle	Hole Residues
A	7	L	123.9852062	KnobIntoHole from A7 to B3 B6 B7 B10
A	8	K	55.68015283	KnobIntoHole from A8 to B2 B5 B6 B9
A	11	A	36.93970819	KnobIntoHole from A11 to B6 B9 B10 B13
A	14	L	125.338209	KnobIntoHole from A14 to B10 B13 B14 B17
A	18	A	39.97229069	KnobIntoHole from A18 to B13 B16 B17 B20
A	21	L	129.9074574	KnobIntoHole from A21 to B17 B20 B21 B24
A	25	A	36.57397903	KnobIntoHole from A25 to B20 B23 B24 B27
A	6	A	57.85328015	KnobIntoHole from A6 to F7 F10 F11 F14
A	13	A	46.060017	KnobIntoHole from A13 to F14 F17 F18 F21
B	6	A	76.60425347	KnobIntoHole from B6 to A8 A4 A7 A11
B	10	L	35.21407441	KnobIntoHole from B10 to A7 A10 A11 A14
B	13	A	64.24645895	KnobIntoHole from B13 to A11 A14 A15 A18
B	17	L	39.38851001	KnobIntoHole from B17 to A14 A17 A18 A21
B	20	A	59.68594966	KnobIntoHole from B20 to A18 A21 A22 A25
B	24	L	36.74337805	KnobIntoHole from B24 to A21 A24 A25 A28
B	7	L	119.3553966	KnobIntoHole from B7 to C3 C6 C7 C10
B	8	K	61.42265013	KnobIntoHole from B8 to C2 C5 C6 C9
B	11	A	39.50372408	KnobIntoHole from B11 to C6 C9 C10 C13
B	14	L	120.7336028	KnobIntoHole from B14 to C10 C13 C14 C17
B	15	K	58.95465499	KnobIntoHole from B15 to C9 C12 C13 C16
B	18	A	33.99922752	KnobIntoHole from B18 to C13 C16 C17 C20
B	21	L	124.8351464	KnobIntoHole from B21 to C17 C20 C21 C24
B	25	A	32.09916476	KnobIntoHole from B25 to C20 C23 C24 C27
C	6	A	69.85478527	KnobIntoHole from C6 to B4 B7 B8 B11
C	10	L	37.24068899	KnobIntoHole from C10 to B7 B10 B11 B14
C	13	A	62.88213867	KnobIntoHole from C13 to B11 B14 B15 B18
C	17	L	34.106602	KnobIntoHole from C17 to B14 B17 B18 B21
C	20	A	59.33938493	KnobIntoHole from C20 to B18 B21 B22 B25
C	24	L	31.36862232	KnobIntoHole from C24 to B21 B24 B25 B28
C	14	L	48.66502098	KnobIntoHole from C14 to D6 D9 D10 D13
C	21	L	54.24989339	KnobIntoHole from C21 to D13 D16 D17 D20
D	6	A	57.85328015	KnobIntoHole from D6 to C7 C10 C11 C14
D	13	A	46.060017	KnobIntoHole from D13 to C14 C17 C18 C21
D	7	L	123.9852062	KnobIntoHole from D7 to E3 E6 E7 E10
D	8	K	55.68015283	KnobIntoHole from D8 to E2 E5 E6 E9
D	11	A	36.93970819	KnobIntoHole from D11 to E6 E9 E10 E13
D	14	L	125.338209	KnobIntoHole from D14 to E10 E13 E14 E17
D	18	A	39.97229069	KnobIntoHole from D18 to E13 E16 E17 E20
D	21	L	129.9074574	KnobIntoHole from D21 to E17 E20 E21 E24
D	25	A	36.57397903	KnobIntoHole from D25 to E20 E23 E24 E27
E	6	A	76.60425347	KnobIntoHole from E6 to D8 D4 D7 D11
E	10	L	35.21407441	KnobIntoHole from E10 to D7 D10 D11 D14
E	13	A	64.24645895	KnobIntoHole from E13 to D11 D14 D15 D18

Chain	Residue	Amino Acid	Crick Angle	Hole Residues
E	17	L	39.38851001	KnobIntoHole from E17 to D14 D17 D18 D21
E	20	A	59.68594966	KnobIntoHole from E20 to D18 D21 D22 D25
E	24	L	36.74337805	KnobIntoHole from E24 to D21 D24 D25 D28
E	7	L	119.3553966	KnobIntoHole from E7 to F3 F6 F7 F10
E	8	K	61.42265013	KnobIntoHole from E8 to F2 F5 F6 F9
E	11	A	39.50372408	KnobIntoHole from E11 to F6 F9 F10 F13
E	14	L	120.7336028	KnobIntoHole from E14 to F10 F13 F14 F17
E	15	K	58.95465499	KnobIntoHole from E15 to F9 F12 F13 F16
E	18	A	33.99922752	KnobIntoHole from E18 to F13 F16 F17 F20
E	21	L	124.8351464	KnobIntoHole from E21 to F17 F20 F21 F24
E	25	A	32.09916476	KnobIntoHole from E25 to F20 F23 F24 F27
F	14	L	48.66502098	KnobIntoHole from F14 to A6 A9 A10 A13
F	21	L	54.24989339	KnobIntoHole from F21 to A13 A16 A17 A20
F	6	A	69.85478527	KnobIntoHole from F6 to E4 E7 E8 E11
F	10	L	37.24068899	KnobIntoHole from F10 to E7 E10 E11 E14
F	13	A	62.88213867	KnobIntoHole from F13 to E11 E14 E15 E18
F	17	L	34.106602	KnobIntoHole from F17 to E14 E17 E18 E21
F	20	A	59.33938493	KnobIntoHole from F20 to E18 E21 E22 E25
F	24	L	31.36862232	KnobIntoHole from F24 to E21 E24 E25 E28

Supplementary Table 4 - Identified knobs-into-holes interactions in the crystal structure.

CC-Type2-IL-Sg iSOCKET Knobs-into-holes interactions

Chain	Residue	Amino Acid	Crick Angle	Hole Residues
A	6	S	59.08674786	KnobIntoHole from A6 to B7 B10 B11 B14
A	13	S	59.91728947	KnobIntoHole from A13 to B14 B17 B18 B21
A	20	S	54.7933729	KnobIntoHole from A20 to B21 B24 B25 B28
A	8	K	24.07488374	KnobIntoHole from A8 to F3 F6 F7 F10
A	11	A	96.44530005	KnobIntoHole from A11 to F7 F10 F11 F14
A	15	K	23.63905377	KnobIntoHole from A15 to F10 F13 F14 F17
A	18	A	93.07185816	KnobIntoHole from A18 to F14 F17 F18 F21
A	22	K	19.03232794	KnobIntoHole from A22 to F17 F20 F21 F24
A	25	A	91.48037107	KnobIntoHole from A25 to F21 F24 F25 F28
B	14	I	53.76986911	KnobIntoHole from B14 to A6 A9 A10 A13
B	17	L	119.0727594	KnobIntoHole from B17 to A10 A13 A14 A17
B	21	I	54.48471886	KnobIntoHole from B21 to A13 A16 A17 A20
B	28	I	50.32027438	KnobIntoHole from B28 to A20 A23 A24 A27
B	6	S	72.20993912	KnobIntoHole from B6 to C3 C4 C7 C8
B	10	L	39.11818241	KnobIntoHole from B10 to C7 C10 C11 C14
B	13	S	63.23733891	KnobIntoHole from B13 to C14 C11 C15 C10
B	17	L	38.78135238	KnobIntoHole from B17 to C14 C17 C18 C21
B	20	S	64.39624773	KnobIntoHole from B20 to C21 C18 C22 C17
B	24	L	38.77526505	KnobIntoHole from B24 to C21 C24 C25 C28

Chain	Residue	Amino Acid	Crick Angle	Hole Residues
C	7	I	124.9823325	KnobIntoHole from C7 to B3 B6 B7 B10
C	14	I	121.1415683	KnobIntoHole from C14 to B10 B13 B14 B17
C	18	A	36.2939725	KnobIntoHole from C18 to B13 B16 B17 B20
C	21	I	116.7046228	KnobIntoHole from C21 to B17 B20 B21 B24
C	25	A	33.64397051	KnobIntoHole from C25 to B20 B23 B24 B27
C	6	S	65.12560711	KnobIntoHole from C6 to D4 D7 D8 D11
C	10	L	36.72871867	KnobIntoHole from C10 to D7 D10 D11 D14
C	17	L	28.3173748	KnobIntoHole from C17 to D14 D17 D18 D21
C	24	L	29.5993386	KnobIntoHole from C24 to D21 D24 D25 D28
D	7	I	118.6774586	KnobIntoHole from D7 to C3 C6 C7 C10
D	11	A	37.17884973	KnobIntoHole from D11 to C6 C9 C10 C13
D	14	I	111.6169921	KnobIntoHole from D14 to C10 C13 C14 C17
D	21	I	110.6004798	KnobIntoHole from D21 to C17 C20 C21 C24
D	10	L	35.49611546	KnobIntoHole from D10 to E7 E10 E11 E14
D	17	L	37.07966546	KnobIntoHole from D17 to E14 E17 E18 E21
D	24	L	36.06195377	KnobIntoHole from D24 to E21 E24 E25 E28
E	10	L	38.75436725	KnobIntoHole from E10 to A7 A10 A11 A14
E	17	L	38.15456292	KnobIntoHole from E17 to A14 A17 A18 A21
E	24	L	36.62431313	KnobIntoHole from E24 to A21 A24 A25 A28
E	7	I	115.0521485	KnobIntoHole from E7 to D3 D6 D7 D10
E	8	K	57.36479445	KnobIntoHole from E8 to D2 D5 D6 D9
E	14	I	119.9943035	KnobIntoHole from E14 to D10 D13 D14 D17
E	21	I	122.6771327	KnobIntoHole from E21 to D17 D20 D21 D24
E	6	S	56.97757942	KnobIntoHole from E6 to F4 F7 F8 F11
E	20	S	52.55707636	KnobIntoHole from E20 to F18 F21 F22 F25
E	24	L	49.03415102	KnobIntoHole from E24 to F21 F24 F25 F28
F	7	I	24.22960276	KnobIntoHole from F7 to A4 A7 A8 A11
F	10	L	93.93793955	KnobIntoHole from F10 to A8 A11 A12 A15
F	14	I	25.74598859	KnobIntoHole from F14 to A11 A14 A15 A18
F	17	L	97.73974048	KnobIntoHole from F17 to A15 A18 A19 A22
F	21	I	21.653659	KnobIntoHole from F21 to A18 A21 A22 A25
F	24	L	94.92486684	KnobIntoHole from F24 to A22 A25 A26 A29
F	7	I	124.6527451	KnobIntoHole from F7 to E3 E6 E7 E10
F	11	A	43.89401235	KnobIntoHole from F11 to E6 E9 E10 E13
F	14	I	121.4417266	KnobIntoHole from F14 to E10 E13 E14 E17
F	21	I	126.4395494	KnobIntoHole from F21 to E17 E20 E21 E24
F	25	A	48.50925375	KnobIntoHole from F25 to E20 E23 E24 E27

Supplementary Table 5 - Identified knobs-into-holes interactions in the crystal structure.

CC-Type2-FI iSOCKET Knobs-into-holes interactions

Chain	Residue	Amino Acid	Crick Angle	Hole Residues
A	6	A	97.44271034	KnobIntoHole from A6 to B7 B8 B10 B11

Chain	Residue	Amino Acid	Crick Angle	Hole Residues
A	10	I	32.4052092	KnobIntoHole from A10 to B7 B10 B11 B14
A	13	A	95.33708697	KnobIntoHole from A13 to B14 B15 B17 B18
A	17	I	31.96896784	KnobIntoHole from A17 to B14 B17 B18 B21
A	20	A	96.17070311	KnobIntoHole from A20 to B21 B22 B24 B25
A	11	A	57.72207321	KnobIntoHole from A11 to F3 F6 F7 F10
A	15	K	35.96987962	KnobIntoHole from A15 to F6 F9 F10 F13
A	18	A	57.23296607	KnobIntoHole from A18 to F10 F13 F14 F17
A	22	K	33.24724332	KnobIntoHole from A22 to F13 F16 F17 F20
A	25	A	59.87210751	KnobIntoHole from A25 to F17 F20 F21 F24
B	7	F	118.4858408	KnobIntoHole from B7 to A3 A6 A7 A10
B	14	F	114.3784942	KnobIntoHole from B14 to A10 A13 A14 A17
B	21	F	116.3302642	KnobIntoHole from B21 to A17 A20 A21 A24
B	6	A	74.64441966	KnobIntoHole from B6 to C4 C7 C8 C11
B	10	I	30.60997005	KnobIntoHole from B10 to C7 C10 C11 C14
B	13	A	105.4541644	KnobIntoHole from B13 to C18 C15 C14 C11
B	17	I	28.68367616	KnobIntoHole from B17 to C14 C17 C18 C21
B	20	A	72.92448644	KnobIntoHole from B20 to C25 C21 C22 C24
C	7	F	122.7871322	KnobIntoHole from C7 to B3 B6 B7 B10
C	11	A	31.82990744	KnobIntoHole from C11 to B6 B9 B10 B13
C	14	F	127.8042116	KnobIntoHole from C14 to B10 B13 B14 B17
C	18	A	33.58520666	KnobIntoHole from C18 to B13 B16 B17 B20
C	21	F	129.1668129	KnobIntoHole from C21 to B17 B20 B21 B24
C	10	I	40.42654461	KnobIntoHole from C10 to D4 D7 D8 D11
C	14	F	47.25353415	KnobIntoHole from C14 to D7 D10 D11 D14
C	17	I	38.15914393	KnobIntoHole from C17 to D11 D14 D15 D18
C	21	F	46.90230639	KnobIntoHole from C21 to D14 D17 D18 D21
C	24	I	36.39165947	KnobIntoHole from C24 to D18 D21 D22 D25
D	11	A	61.06453607	KnobIntoHole from D11 to C10 C13 C14 C17
D	15	K	32.01517518	KnobIntoHole from D15 to C13 C16 C17 C20
D	18	A	61.03918613	KnobIntoHole from D18 to C17 C20 C21 C24
D	22	K	35.47882812	KnobIntoHole from D22 to C20 C23 C24 C27
D	6	A	93.91890042	KnobIntoHole from D6 to E7 E8 E10 E11
D	10	I	32.2038181	KnobIntoHole from D10 to E7 E10 E11 E14
D	13	A	95.26281996	KnobIntoHole from D13 to E14 E15 E17 E18
D	17	I	30.93332339	KnobIntoHole from D17 to E14 E17 E18 E21
D	20	A	94.20660422	KnobIntoHole from D20 to E21 E22 E24 E25
D	24	I	31.38441878	KnobIntoHole from D24 to E21 E24 E25 E28
E	7	F	113.1759868	KnobIntoHole from E7 to D3 D6 D7 D10
E	14	F	117.0339547	KnobIntoHole from E14 to D10 D13 D14 D17
E	15	K	71.05249895	KnobIntoHole from E15 to D9 D12 D13 D16
E	21	F	121.8369567	KnobIntoHole from E21 to D17 D20 D21 D24
E	6	A	109.3424969	KnobIntoHole from E6 to F11 F8 F7 F4
E	10	I	30.85785076	KnobIntoHole from E10 to F7 F10 F11 F14
E	13	A	108.1047481	KnobIntoHole from E13 to F18 F15 F14 F11
E	17	I	31.61805651	KnobIntoHole from E17 to F14 F17 F18 F21
E	20	A	80.44303789	KnobIntoHole from E20 to F18 F21 F24 F25

Chain	Residue	Amino Acid	Crick Angle	Hole Residues
F	3	I	51.75059708	KnobIntoHole from F3 to A4 A7 A8 A11
F	7	F	47.41780171	KnobIntoHole from F7 to A7 A10 A11 A14
F	10	I	41.71985225	KnobIntoHole from F10 to A11 A14 A15 A18
F	14	F	45.69258037	KnobIntoHole from F14 to A14 A17 A18 A21
F	17	I	42.02541447	KnobIntoHole from F17 to A18 A21 A22 A25
F	21	F	40.02823115	KnobIntoHole from F21 to A21 A24 A25 A28
F	7	F	126.9531399	KnobIntoHole from F7 to E3 E6 E7 E10
F	11	A	31.93990613	KnobIntoHole from F11 to E6 E9 E10 E13
F	14	F	127.4667793	KnobIntoHole from F14 to E10 E13 E14 E17
F	18	A	32.97820831	KnobIntoHole from F18 to E13 E16 E17 E20
F	21	F	134.0255407	KnobIntoHole from F21 to E17 E20 E21 E24
F	25	A	70.95837533	KnobIntoHole from F25 to E19 E20 E23 E24

Supplementary Table 6 - Identified knobs-into-holes interactions in the crystal structure.

CC-Type2-LF iSOCKET Knobs-into-holes interactions

Chain	Residue	Amino Acid	Crick Angle	Hole Residues
A	2	E	129.3409683	KnobIntoHole from A2 to B8 B11 B12 B15
A	3	F	50.33932504	KnobIntoHole from A3 to B7 B10 B11 B14
A	6	A	47.02021707	KnobIntoHole from A6 to B11 B14 B15 B18
A	9	E	128.2730839	KnobIntoHole from A9 to B15 B18 B19 B22
A	10	F	40.23726734	KnobIntoHole from A10 to B14 B17 B18 B21
A	13	A	52.4339522	KnobIntoHole from A13 to B18 B21 B22 B25
A	17	F	41.01833806	KnobIntoHole from A17 to B21 B24 B25 B28
A	8	K	26.46915403	KnobIntoHole from A8 to H3 H6 H10 H7
A	15	K	39.35741883	KnobIntoHole from A15 to H10 H13 H14 H17
A	22	K	38.07166918	KnobIntoHole from A22 to H17 H20 H21 H24
B	14	L	129.4476712	KnobIntoHole from B14 to A3 A6 A7 A10
B	15	K	43.09042228	KnobIntoHole from B15 to A2 A5 A6 A9
B	18	A	42.55803392	KnobIntoHole from B18 to A6 A9 A10 A13
B	21	L	128.2876026	KnobIntoHole from B21 to A10 A13 A14 A17
B	22	K	46.3281655	KnobIntoHole from B22 to A9 A12 A13 A16
B	25	A	43.07193783	KnobIntoHole from B25 to A13 A16 A17 A20
B	28	L	125.7571245	KnobIntoHole from B28 to A17 A20 A21 A24
B	6	A	47.53047594	KnobIntoHole from B6 to C4 C7 C8 C11
B	10	F	47.75608398	KnobIntoHole from B10 to C7 C10 C11 C14
B	13	A	51.00911137	KnobIntoHole from B13 to C11 C14 C15 C18
B	17	F	53.52254632	KnobIntoHole from B17 to C14 C17 C18 C21
B	20	A	45.44961501	KnobIntoHole from B20 to C18 C21 C22 C25
B	23	E	115.9422306	KnobIntoHole from B23 to C22 C25 C26 C29
B	24	F	52.37036717	KnobIntoHole from B24 to C21 C24 C25 C28
B	10	F	59.78132673	KnobIntoHole from B10 to E7 E10 E11 E14
B	17	F	54.0325834	KnobIntoHole from B17 to E14 E17 E18 E21

Chain	Residue	Amino Acid	Crick Angle	Hole Residues
B	24	F	55.7076088	KnobIntoHole from B24 to E21 E24 E25 E28
C	7	L	127.6764291	KnobIntoHole from C7 to B3 B6 B7 B10
C	8	K	46.07612584	KnobIntoHole from C8 to B2 B5 B6 B9
C	11	A	48.06183319	KnobIntoHole from C11 to B6 B9 B10 B13
C	14	L	125.2763281	KnobIntoHole from C14 to B10 B13 B14 B17
C	18	A	52.87302495	KnobIntoHole from C18 to B13 B16 B17 B20
C	21	L	131.0847365	KnobIntoHole from C21 to B17 B20 B21 B24
C	25	A	59.81079791	KnobIntoHole from C25 to B20 B23 B24 B27
C	6	A	73.12110751	KnobIntoHole from C6 to D4 D7 D8 D11
C	10	F	32.01129028	KnobIntoHole from C10 to D7 D10 D11 D14
C	16	E	133.0844828	KnobIntoHole from C16 to D15 D18 D19 D22
C	17	F	31.01327541	KnobIntoHole from C17 to D14 D17 D18 D21
C	20	A	67.22704519	KnobIntoHole from C20 to D18 D21 D22 D25
C	24	F	26.54924196	KnobIntoHole from C24 to D21 D24 D25 D28
D	7	L	110.5307669	KnobIntoHole from D7 to C3 C6 C7 C10
D	11	A	34.17483573	KnobIntoHole from D11 to C6 C9 C10 C13
D	14	L	112.9764291	KnobIntoHole from D14 to C10 C13 C14 C17
D	21	L	116.2283775	KnobIntoHole from D21 to C17 C20 C21 C24
D	25	A	30.85415905	KnobIntoHole from D25 to C20 C23 C24 C27
D	10	F	63.6076973	KnobIntoHole from D10 to E8 E11 E12 E15
D	17	F	66.03523356	KnobIntoHole from D17 to E15 E18 E19 E22
E	8	K	40.26068756	KnobIntoHole from E8 to D3 D6 D7 D10
E	15	K	36.71747929	KnobIntoHole from E15 to D10 D13 D14 D17
E	22	K	36.46430967	KnobIntoHole from E22 to D17 D20 D21 D24
E	6	A	51.10725638	KnobIntoHole from E6 to F4 F7 F8 F11
E	10	F	43.52796146	KnobIntoHole from E10 to F7 F10 F11 F14
E	13	A	54.9561738	KnobIntoHole from E13 to F11 F14 F15 F18
E	17	F	37.57028818	KnobIntoHole from E17 to F14 F17 F18 F21
E	20	A	54.48817444	KnobIntoHole from E20 to F18 F21 F22 F25
E	24	F	39.52561246	KnobIntoHole from E24 to F21 F24 F25 F28
F	10	F	57.8407591	KnobIntoHole from F10 to A7 A10 A11 A14
F	17	F	57.43895041	KnobIntoHole from F17 to A14 A17 A18 A21
F	24	F	59.34550035	KnobIntoHole from F24 to A21 A24 A25 A28
F	7	L	128.0516752	KnobIntoHole from F7 to E3 E6 E7 E10
F	8	K	47.42516418	KnobIntoHole from F8 to E2 E5 E6 E9
F	11	A	46.86611201	KnobIntoHole from F11 to E6 E9 E10 E13
F	14	L	128.8694152	KnobIntoHole from F14 to E10 E13 E14 E17
F	18	A	140.3228306	KnobIntoHole from F18 to E20 E17 E16 E13
F	21	L	127.7639683	KnobIntoHole from F21 to E17 E20 E21 E24
F	25	A	42.94287013	KnobIntoHole from F25 to E20 E23 E24 E27
F	6	A	46.61768509	KnobIntoHole from F6 to G4 G7 G8 G11
F	10	F	54.43520759	KnobIntoHole from F10 to G7 G10 G11 G14
F	13	A	43.7708229	KnobIntoHole from F13 to G11 G14 G15 G18
F	16	E	120.7592068	KnobIntoHole from F16 to G15 G18 G19 G22
F	17	F	54.44566732	KnobIntoHole from F17 to G14 G17 G18 G21
F	20	A	40.8155342	KnobIntoHole from F20 to G18 G21 G22 G25

Chain	Residue	Amino Acid	Crick Angle	Hole Residues
F	24	F	53.07419274	KnobIntoHole from F24 to G21 G24 G25 G28
G	7	L	130.4643907	KnobIntoHole from G7 to F3 F6 F7 F10
G	8	K	45.2758577	KnobIntoHole from G8 to F2 F5 F6 F9
G	11	A	52.79511585	KnobIntoHole from G11 to F6 F9 F10 F13
G	14	L	128.0442106	KnobIntoHole from G14 to F10 F13 F14 F17
G	18	A	52.85981982	KnobIntoHole from G18 to F13 F16 F17 F20
G	21	L	130.8556093	KnobIntoHole from G21 to F17 F20 F21 F24
G	25	A	60.00282014	KnobIntoHole from G25 to F20 F23 F24 F27
G	10	F	28.93895257	KnobIntoHole from G10 to H7 H10 H11 H14
G	17	F	28.51593741	KnobIntoHole from G17 to H14 H17 H18 H21
G	20	A	75.0483443	KnobIntoHole from G20 to H18 H21 H22 H25
H	10	F	63.92927486	KnobIntoHole from H10 to A8 A11 A12 A15
H	17	F	66.16679329	KnobIntoHole from H17 to A15 A18 A19 A22
H	7	L	107.522062	KnobIntoHole from H7 to G3 G6 G7 G10
H	14	L	110.7778544	KnobIntoHole from H14 to G10 G13 G14 G17
H	21	L	109.7419949	KnobIntoHole from H21 to G17 G20 G21 G24
H	25	A	28.5481871	KnobIntoHole from H25 to G20 G23 G24 G27

Supplementary Table 7 - Identified knobs-into-holes interactions in the crystal structure.

pH Titration experiments

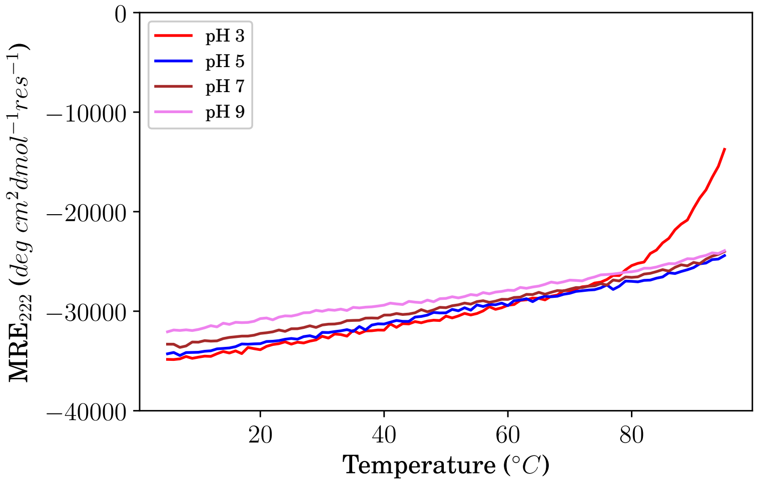

Supplementary Figure 83 CC-Type2-LL-L17Q - Thermal denaturation profile monitored at 222 nm. Conditions: 10 μM peptide concentration, 100 mM NaCl and pH-specific buffer (see materials and methods).

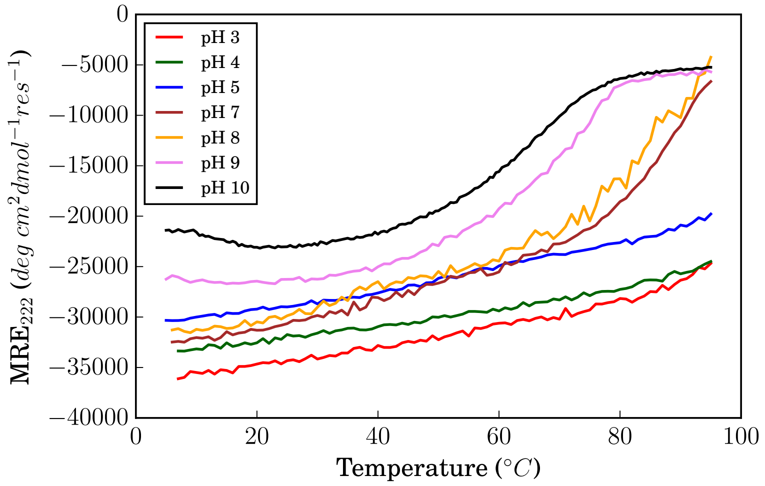

Supplementary Figure 84 CC-Type2-LL-L17E - Thermal denaturation profile monitored at 222 nm. Conditions: 10 μM peptide concentration, 100 mM NaCl and pH-specific buffer (see materials and methods). pH 7 spectrum was too noisy to be presented.

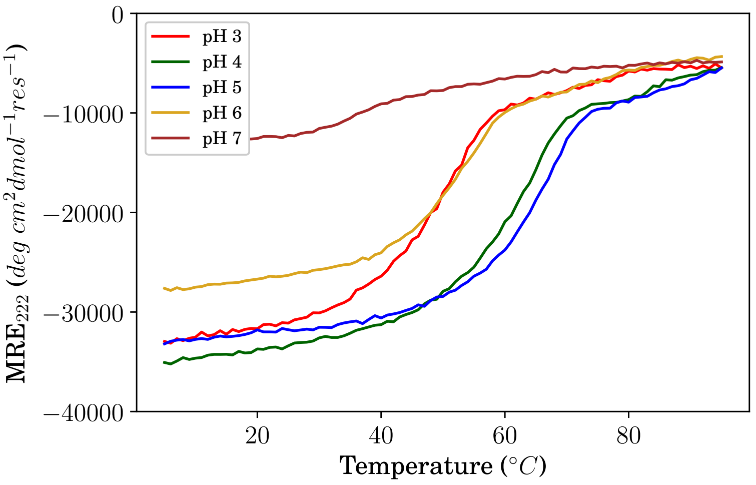

Supplementary Figure 85 CC-Type2-IL-Sg-L17E - Thermal denaturation profile monitored at 222 nm. Conditions: 10 μM peptide concentration, 100 mM NaCl and pH-specific buffer (see materials and methods).

Constant pH replica-exchange molecular dynamics

Supplementary Note 1

Constant pH molecular dynamics simulations - The molecular dynamics package AMBER 16[3] was used to run constant pH molecular dynamics (CpHMD) and pH replica exchange MD (pH-REMD) simulations using the AMBER ff14SB force field.[4] In AMBER 16, the CpHMD method is based on the approach developed by Mongan et al.[5] relying on a discrete protonation state model in which exchanges between different protonation states are attempted throughout the MD simulation using Monte Carlo sampling based on the calculated generalized Born protonation or deprotonation free energy. The method was further extended by Swails et al. so it can be run in explicit solvent[6] and pH-REMD can be used to improve protonation state sampling.[7]

System preparation - Starting from the crystal structures of CC-Type2-LL-L17E (accession code: 6G6C) and CC-Type2-IL-SG-L17E (accession code: 6G69) and keeping all crystallographic water molecules, Maestro's Protein Preparation Wizard (Schrödinger Release 2017-4: Maestro, Schrödinger, LLC, New York, NY, 2017) was used to add hydrogens, choose between possible flipped Gln conformations, cap N- and C- terminal residues with an acetyl and an amine respectively, and optimise the hydrogen bonds network. The resulting structures were solvated with the SOLVATE program developed by H. Grubmüller and V. Groll (http://www.mpibpc.mpg.de/grubmueller/solvate, last visited 12 February 2017) to create a solvation shell of TIP3P water molecules of at least 5 Å around the protein using 8 Gaussians. The tleap program, part of the Amber modelling suite[3] was then used to create a truncated octahedron cell of TIP3P water molecules setting the padding to 6 Å and the closeness to 0.75 Å. The charge of the system was neutralised using NaCl and additional ions were added to simulate a concentration of 0.1 M in NaCl. Using the cpinutil.py program, every ionisable residues of CC-Type2-LL-L17E and CC-Type2-IL-SG-L17E, that is every Lys and Glu residues, were made titratable, leading to a total of 60 and 70 titratable residues respectively.

Minimisation, heating and equilibration - The two systems were minimised in 6 phases, first the water, hydrogen atoms and ions were minimised applying a restraint on the protein atoms of 25 kcal.mol^{-1}.Å^{-2}, then the side chains, except the Lys and Glu residues were minimised, first keeping a 25 kcal.mol^{-1}.Å^{-2} restraint on the backbone as well as on the Lys and Glu residues, and then decreasing it to 10 and then 5 kcal.mol^{-1}.Å^{-2}, finally only the Cα and Lys and Glu atoms were

restrained, first with a 2 then 1 kcal.mol^{-1}.Å^{-2} restraint. Each minimisation phase consisted in up to 1000 steps of steepest descent followed by up to 10000 steps of conjugate gradient. The systems were then heated to 300 K over 100 ps applying a 5 kcal.mol^{-1}.Å^{-2} restraint on backbone atoms as well as on Lys and Glu residues. This was followed by a 500-ps initial equilibration in the NPT ensemble at a temperature of 300 K controlled via Langevin dynamics using a collision frequency of 5 ps^{-1} and a pressure 101325 Pa maintained with a Berendsen barostat and a pressure relaxation time of 1 ps and using a 5 kcal.mol^{-1}.Å^{-2} restraint on backbone atoms, Lys and Glu residues.

Using the same pressure and temperature regulations, a careful equilibration protocol was initiated in which the ionisable residues were allowed to titrate with an exchange between states attempted every 100 steps, using a relaxation dynamic of 100 steps, an implicit salt concentration of 0.1 M and a pH of 4.0 as it is the pH at which both CC-Type2-LL-L17E and CC-Type2-IL-SG-L17E are the most stable experimentally. Although running CpHMD simulation in the NPT ensemble is not recommended in AMBER 16, we found that the fully protonated internal Glu residues (each having 4 protons on the carboxylate, named GL4 in AMBER) led to a destabilisation of the coiled coils yielding significant deviations from the crystal structures, leading in turn to a poor starting point for the CpHMD and pH-REMD simulations. Keeping restraints throughout the NPT equilibrations could prevent this but the relaxation of the protein upon shifting to NVT without restraint would lead to changes in pressure, thereby defeating the purpose of the NPT equilibration. We therefore decided to allow the ionisable residues to titrate during the NPT equilibration, carefully monitoring the equilibration. To further test the validity of our approach we also ran equilibrations in which the protonation states were allowed to change for a short period of time and fixing them again afterwards. We found no differences in the outcome of the equilibration except for a lower sampling of the protonation states and the possibility to stop the simulation at unfavourable protonation states leading to deviations from the crystal structure. In light of these results we therefore performed the NPT equilibration allowing the ionisable residues to titrate.

The NPT CpHMD equilibration was first run for 1 ns using the same 5 kcal.mol^{-1}.Å^{-2} restraint on backbone atoms, Lys and Glu residues, then for 1 ns using 2 kcal.mol^{-1}.Å^{-2} and then another 1 ns using 1 kcal.mol^{-1}.Å^{-2}. The restraint of 1 kcal.mol^{-1}.Å^{-2} was then kept only on the Cα, thereby releasing the Lys and Glu side chains, for 1 ns. The restraint on Cα was decreased to 0.5, 0.25, 0.1 and

0.05 kcal.mol^{-1}.Å^{-2} and the equilibration run for 1, 1, 0.5 and 0.5 ns respectively. This was followed by 2.5 ns of equilibration without restraint in the NPT ensemble. The barostat was then switched off and the equilibration continued without any restraints for 35 ns in the NVT ensemble, with the last frame used as a starting point for the pH-REMD simulations.

pH-REMD simulations - Starting form an equilibrated structure at pH 4.0, two different pH-REMD simulations were started for both CC-Type2-LL-L17E and CC-Type2-IL-SG-L17E. For each protein the first pH-REMD simulation was set up with a protonation state exchange attempt of 50 steps and a relaxation dynamic of 50 steps (parameter set 1) whereas the second simulation was performed using a protonation state exchange attempt rate of 100 steps and a relaxation dynamic of 100 steps (parameter set 2). In both cases the exchange between replicas was attempted every 200 steps. A total of 16 replicas were used to cover a pH range comprised between 3.0 and 10.5, with one replica per 0.5 pH unit. For each parameter set, 25 ns of pH-REMD were run as equilibration followed by 200 ns of production pH-REMD resulting in an aggregate sampling time of 6.4 μs for each structure.

Analysis of the pH-REMD simulations - Protonation state statistics were calculated with the cphstats program part of AMBER 16. MD trajectories were pre-processed (to remove unwanted translational and rotational motions of the protein) and analysed with the CCPTRAJ program.[8] The radii of the channels were calculated using HOLE[9] with the MDAnalysis toolkit.[10]

From the pH-REMD simulations, the pKa of the ionisable residues can be calculated using the generalised Henderson–Hasselbalch (HH) equation or Hill equation:

$$f_d = 1/1 + 10^{n(pK_a - pH)} \quad (8.1)$$

where f_d is the deprotonated fraction and n the Hill coefficient. In the ideal case of an ionisable residue not interacting with any other ionisable residue, n=1 and the standard HH equation describe the titration curve. However, as interactions with other ionisable residues increases, the HH equation will less accurately describe the titration curve, leading to n<1 in the case of anti-cooperativity between protonation state changes or n>1 in the case of cooperativity.

Convergence of the pH-REMD simulations and exchange between replicas - Convergence of

the protonation state sampling for the internal Glu17 residues (the slowest to converge) were confirmed by calculating the cumulative average of the deprotonated fraction (Supplementary Figs. 86 and 87) for the combined two parameter set simulations.

Analysis of the random walk of each replica across the pH ladder reveals that for the CC-Type2-LL-L17E simulations (Supplementary Figs. 88 and 89), replicas sampling the higher pH values tend to exchange only with other high pH walkers (replicas 1 and 14 for parameter set 1 and replicas 9 and 15 for parameter set 2). This is due the fact that at high pH values the conformations of CC-Type2-LL-L17E deviate significantly from the crystal structure (opening and even dissociation of the barrels). Nonetheless, exchanges between lower pH walkers occur occasionally, ensuring an efficient sampling in the pH space. For CC-Type2-IL-SG-L17E simulations, in which less drastic conformational changes are observed, almost every replica walks across the entire pH space (Supplementary Figs. 90 and 91). One major exception is replica 7 with parameter set 2 for which conformations sampled at very high pH were suddenly brought to pH 3 leading to the dissociation of the barrel. This replica was then unable to exchange with higher pH walkers, except for some exchanges at pH 3.5.

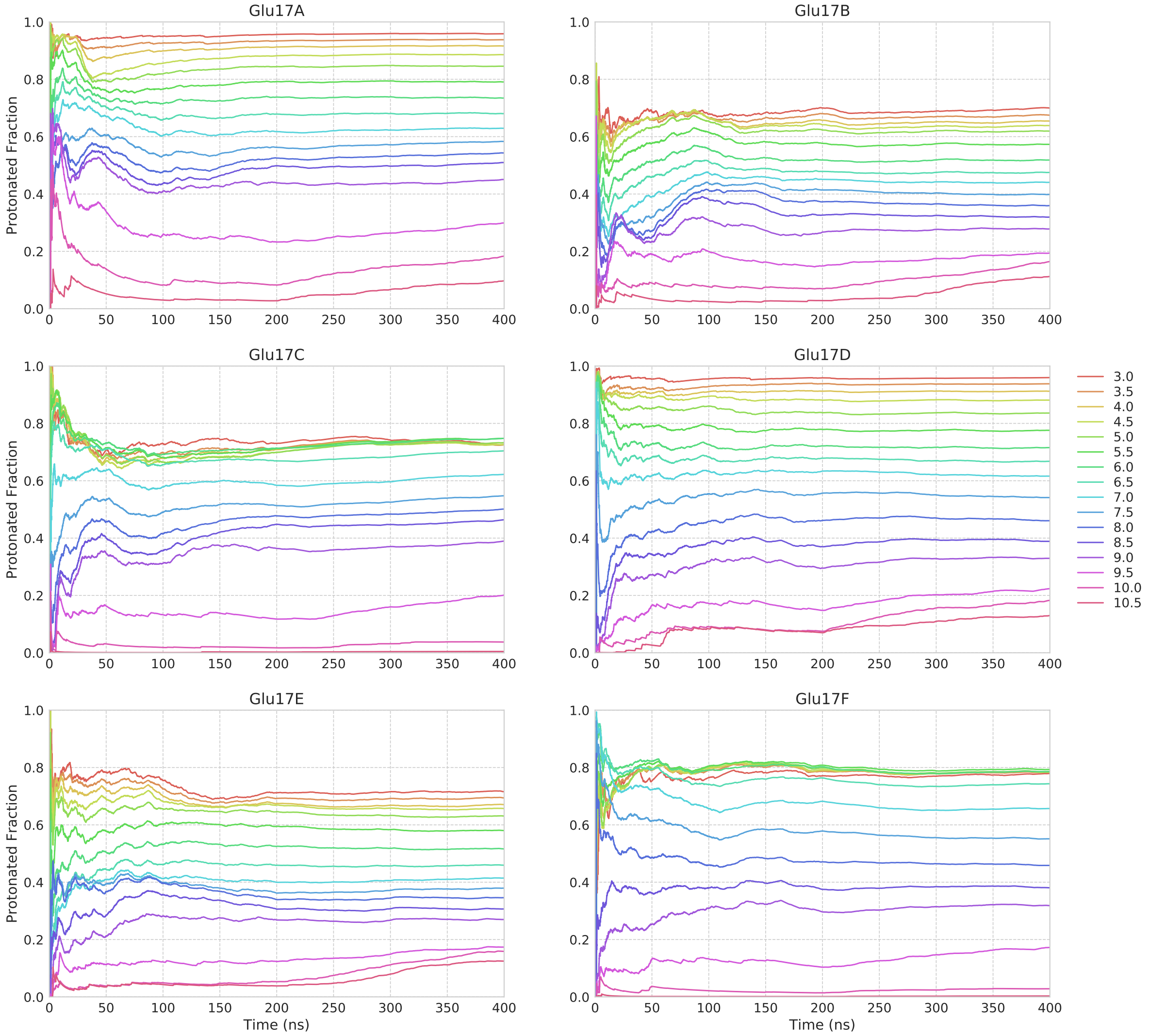

Supplementary Figure 86 Convergence of the protonation state sampling of Glu17 for each chain of CC-Type2-LL-L17E - Cumulative average of the protonated fraction of Glu17 for each chain at each studied pH as a function of simulation time obtained by combining results from the two different sets of parameters.

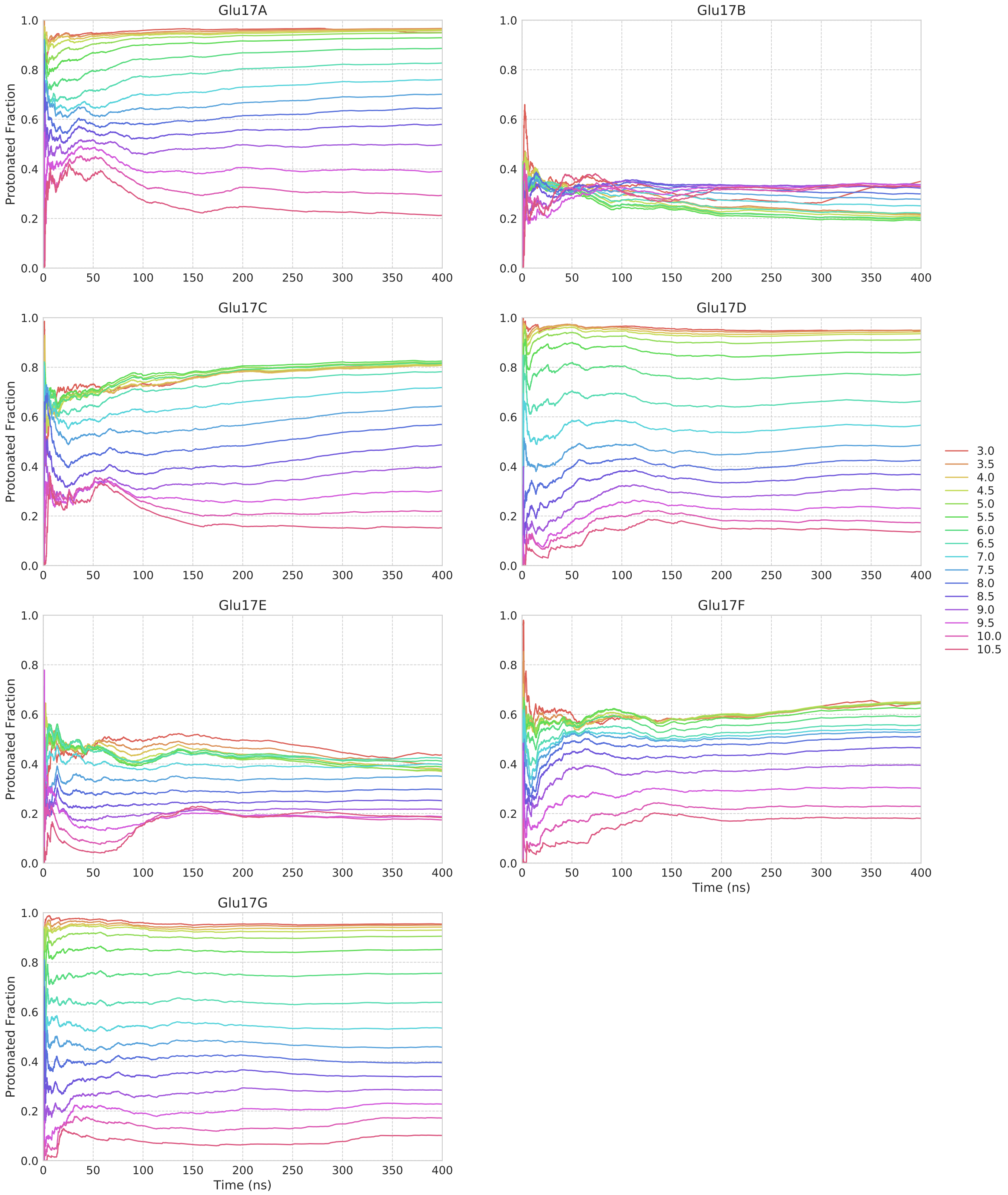

Supplementary Figure 87 Convergence of the protonation state sampling of Glu17 for each chain of CC-Type2-IL-Sg-L17E - Cumulative average of the protonated fraction of Glu17 for each chain at each studied pH as a function of simulation time obtained by combining results from the two different sets of parameters.

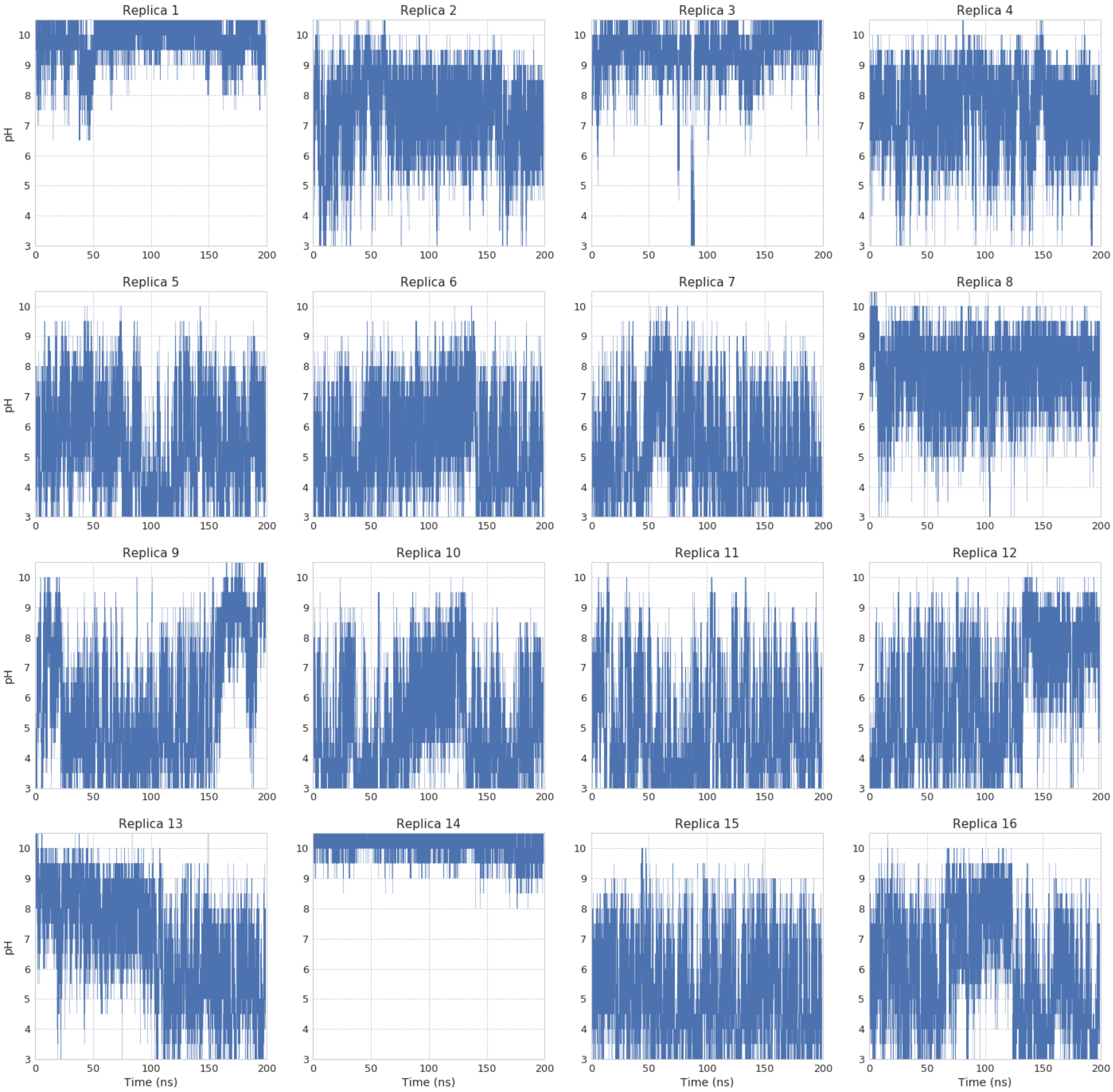

Supplementary Figure 88 Replica walk in pH space for CC-Type2-LL-L17E pH-REMD simulations parameter set 1 - Walk of each replica across the pH ladder as a function of simulation time.

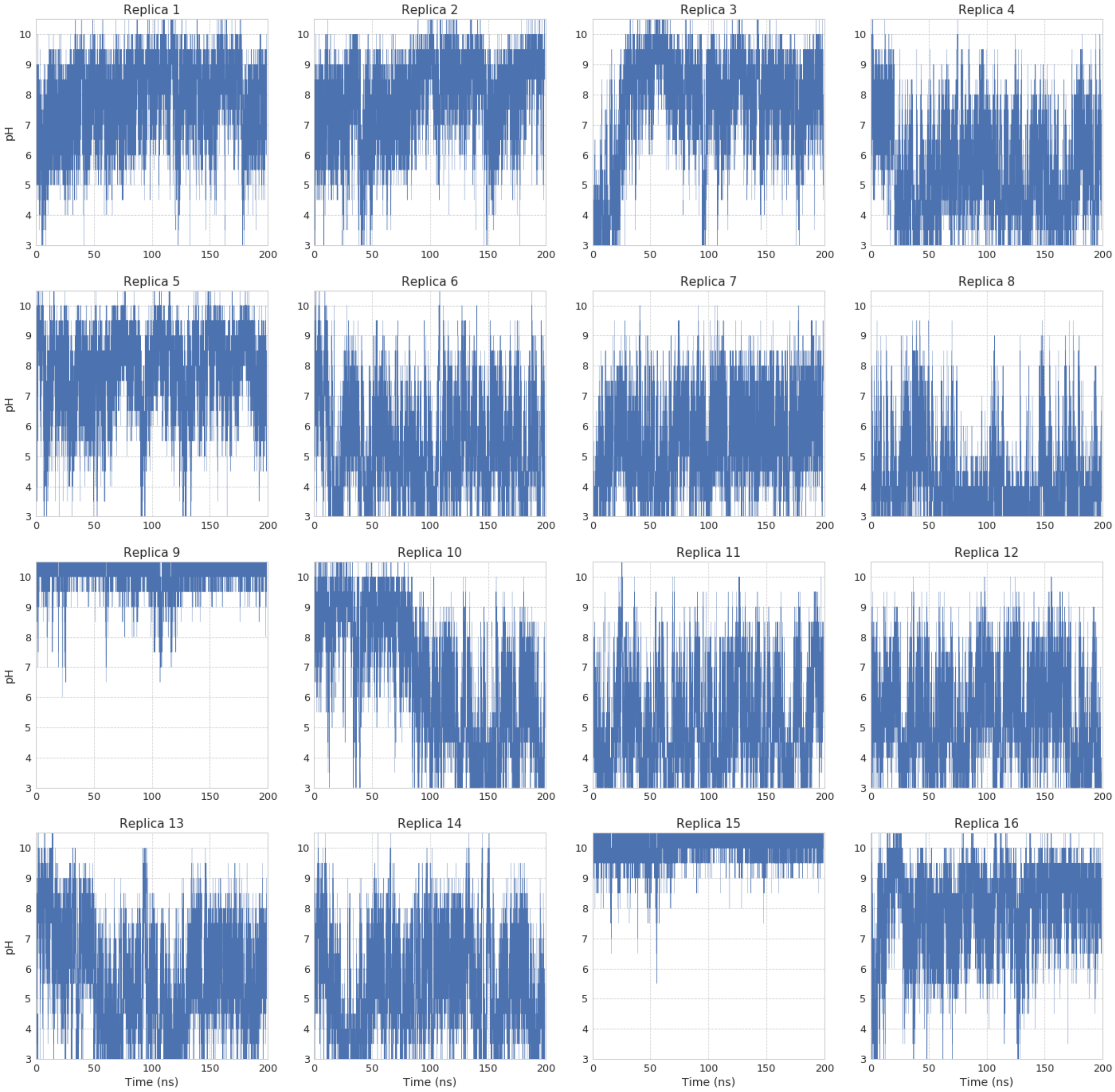

Supplementary Figure 89 Replica walk in pH space for CC-Type2-LL-L17E pH-REMD simulations parameter set 2 - Walk of each replica across the pH ladder as a function of simulation time.

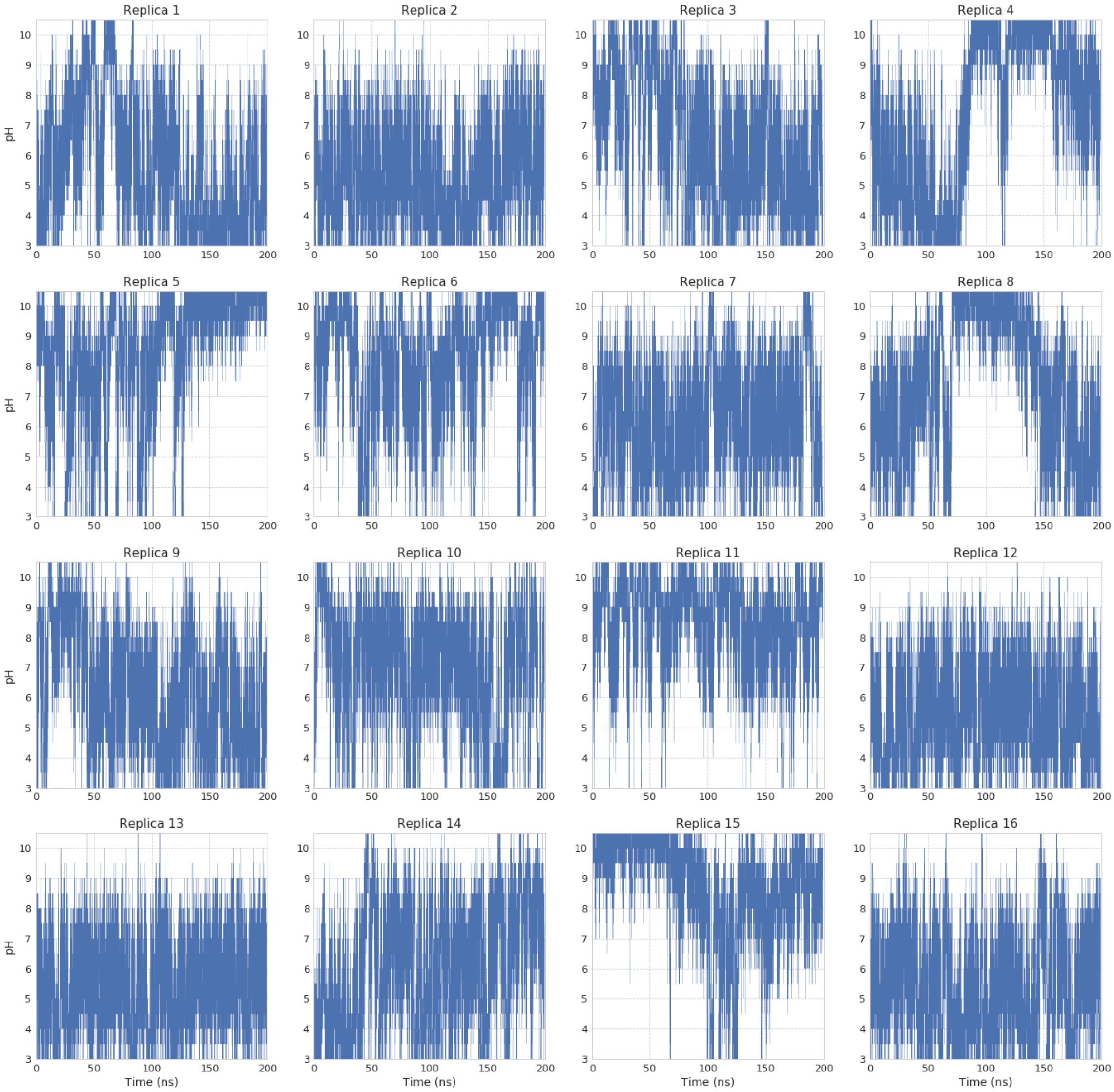

Supplementary Figure 90 Replica walk in pH space for CC-Type2-IL-Sg-L17E pH-REMD simulations parameter set 1 - Walk of each replica across the pH ladder as a function of simulation time.

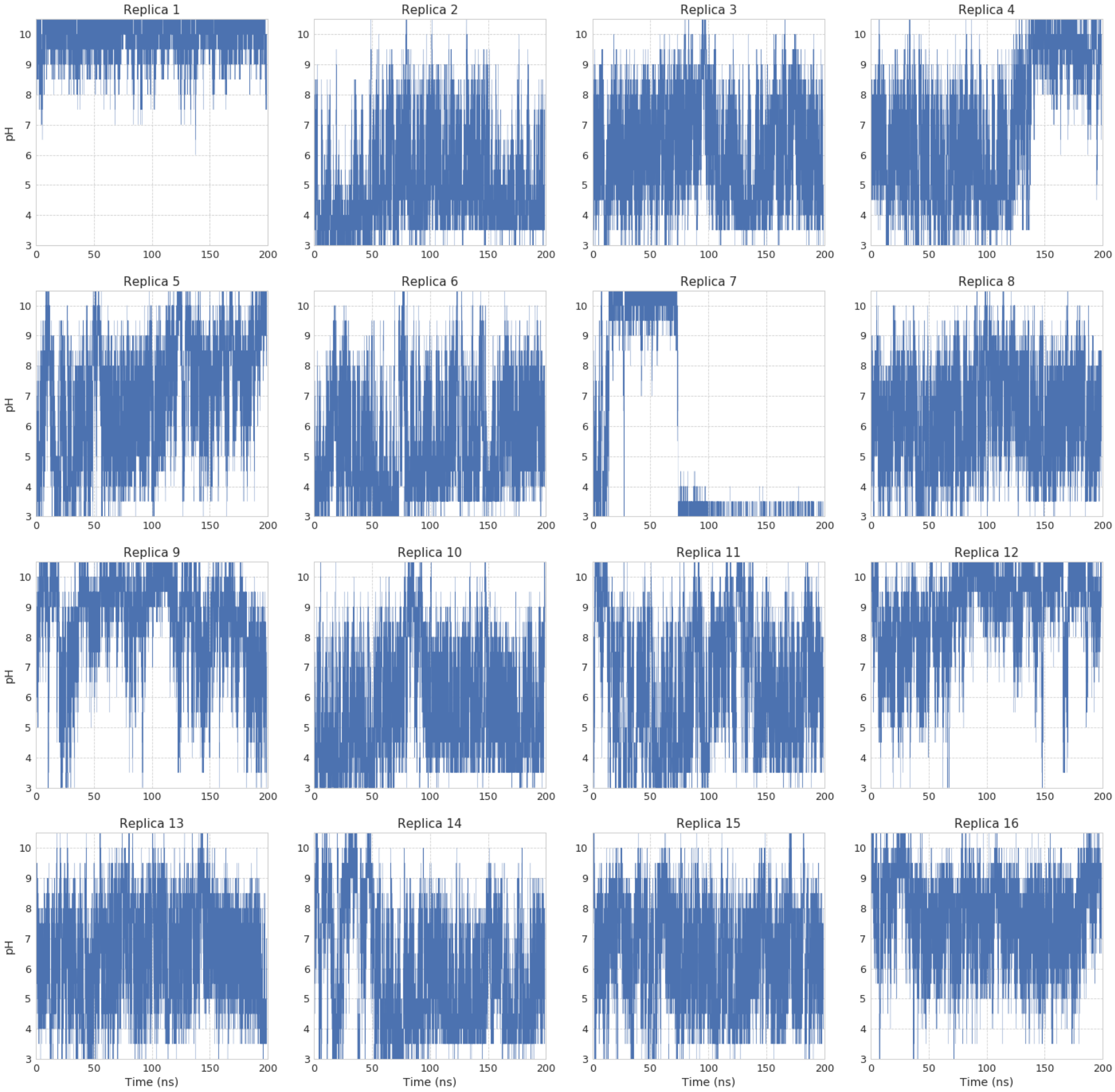

Supplementary Figure 91 Replica walk in pH space for CC-Type2-IL-Sg-L17E pH-REMD simulations parameter set 2 - Walk of each replica across the pH ladder as a function of simulation time.

Transition	pK_a	Hill coefficient
$(\mathrm{Glu17})_6^{0}$ to $(\mathrm{Glu17})_6^{1-}$	not sampled	not sampled
$(\mathrm{Glu17})_6^{1-}$ to $(\mathrm{Glu17})_6^{2-}$	4.7	0.5
$(\mathrm{Glu17})_6^{2-}$ to $(\mathrm{Glu17})_6^{3-}$	6.9	0.8
$(\mathrm{Glu17})_6^{3-}$ to $(\mathrm{Glu17})_6^{4-}$	8.3	0.7
$(\mathrm{Glu17})_6^{4-}$ to $(\mathrm{Glu17})_6^{5-}$	9.5	1.0
$(\mathrm{Glu17})_6^{5-}$ to $(\mathrm{Glu17})_6^{6-}$	10.0	0.9

Supplementary Table 8 pH-REMD modelled macroscopic acid-dissociation constants for Glu residues at the 17th positions of CC-Type2-LL-L17E. The hexameric structure of CC-Type2-LL-L17E was treated as a single polyprotic acid with six titratable groups $(\mathrm{Glu17})_6$. The pK_a values are estimated by fitting the Hill equation $f_d = 1/(1+10^{n(pK_a-pH)})$, where f_d is the deprotonated fraction and n the Hill coefficient.

Transition	pK_a	Hill coefficient
$(\mathrm{Glu17})_7^{0}$ to $(\mathrm{Glu17})_7^{1-}$	not sampled	not sampled
$(\mathrm{Glu17})_7^{1-}$ to $(\mathrm{Glu17})_7^{2-}$	2.5	1.4
$(\mathrm{Glu17})_7^{2-}$ to $(\mathrm{Glu17})_7^{3-}$	5.9	0.7
$(\mathrm{Glu17})_7^{3-}$ to $(\mathrm{Glu17})_7^{4-}$	7.5	0.7
$(\mathrm{Glu17})_7^{4-}$ to $(\mathrm{Glu17})_7^{5-}$	8.9	0.7
$(\mathrm{Glu17})_7^{5-}$ to $(\mathrm{Glu17})_7^{6-}$	10.1	0.7
$(\mathrm{Glu17})_7^{6-}$ to $(\mathrm{Glu17})_7^{7-}$	11.8	0.6

Supplementary Table 9 pH-REMD modelled macroscopic acid-dissociation constants for Glu residues at the 17th positions of CC-Type2-IL-Sg-L17E. The heptameric structure of CC-Type2-LL-L17E was treated as a single polyprotic acid with seven titratable groups $(\mathrm{Glu17})_7$. The pK_a values are estimated by fitting the Hill equation $f_d = 1/(1+10^{n(pK_a-pH)})$, where f_d is the deprotonated fraction and n the Hill coefficient.

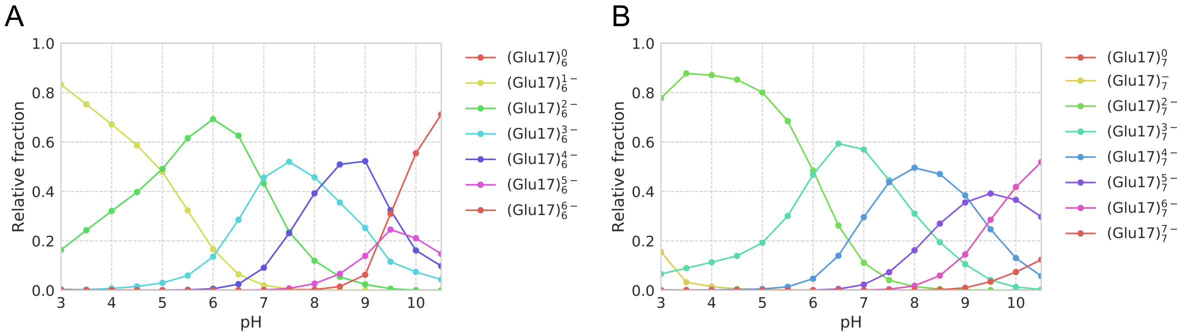

Supplementary Figure 92 Probability of charge states at a given pH for CC-Type2-LL-L17E and CC-Type2-IL-Sg-L17E - Charge state distribution for (A) the Glu17 hexad of CC-Type2-LL-L17E (B) and the Glu17 heptad of CC-Type2-IL-Sg-L17E.

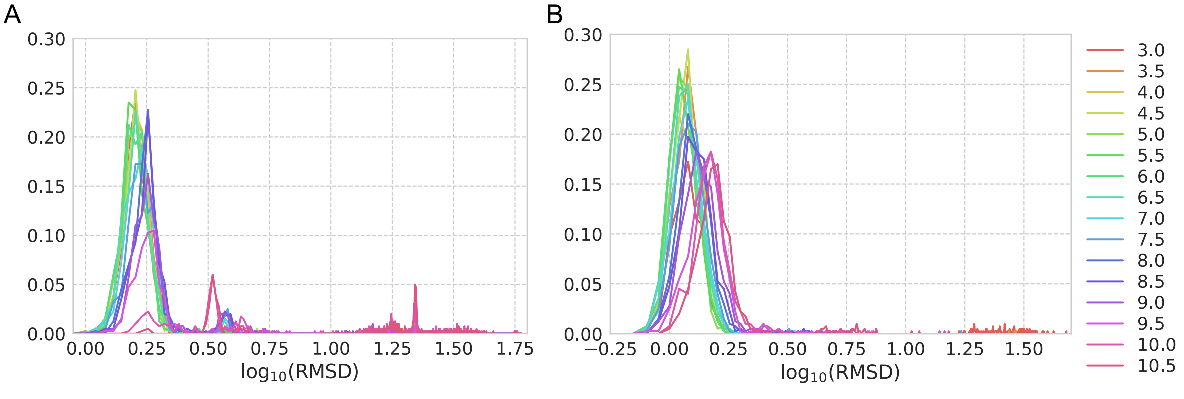

Supplementary Figure 93 Normalised distribution of the backbone root mean square deviation (RMSD) of (A) CC-Type2-LL-L17E and (B) CC-Type2-IL-Sg-L17E between pH 3 and 10.5 - The X-ray crystal structures of CC-Type2-LL-L17E and CC-Type2-IL-Sg-L17E were used as reference for the calculations. Curves are coloured by pH value according to the key on the right-hand side. A logarithmic RMSD scale was used so that large RMSD values corresponding to dissociation of the chains can be visualised.

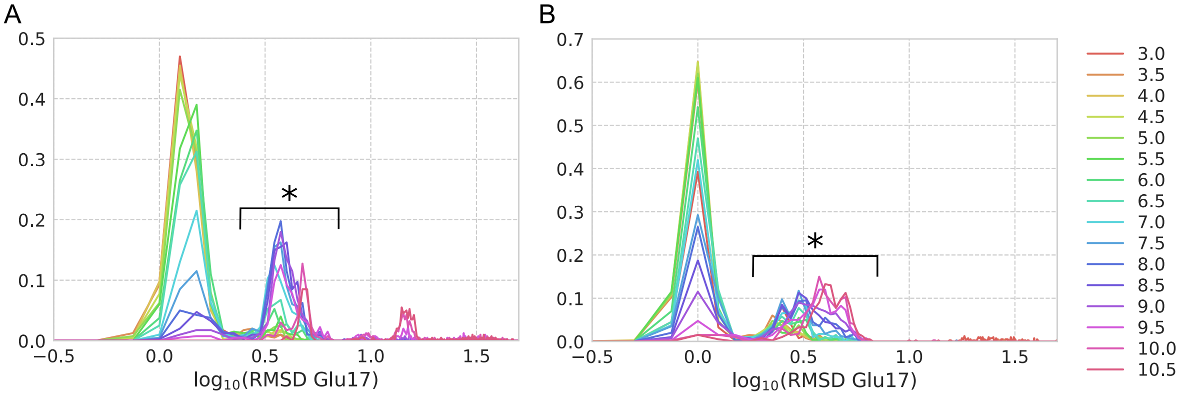

Supplementary Figure 94 Normalised distribution of the RMSD of the inner ring of Glu17 residues of (A) CC-Type2-LL-L17E and (B) CC-Type2-IL-Sg-L17E between pH 3 and 10.5 - The X-ray crystal structures of CC-Type2-LL-L17E and CC-Type2-IL-Sg-L17E were used as reference for the calculations. All the atoms except hydrogen atoms were used for the calculations. The regions indicated with an asterisk correspond to the populations of structures for which one or more Glu17 has its side chain pointing toward the outside of the structure. Curves are coloured by pH value according to the key on the right-hand side. A logarithmic RMSD scale was used so that large RMSD values corresponding to dissociation of chains can be visualised.

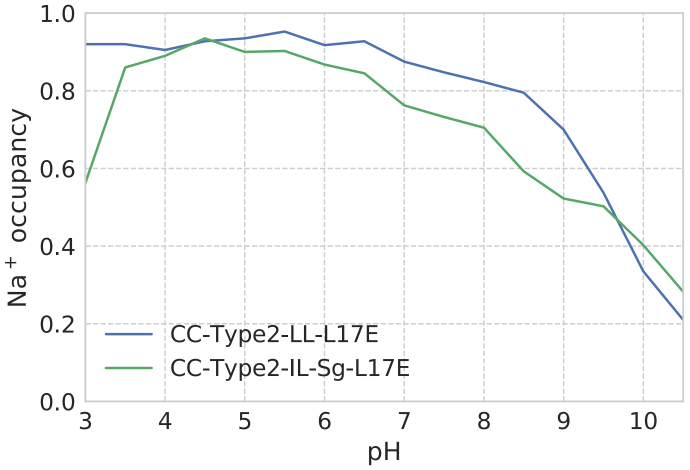

Supplementary Figure 95 Occupancy of a sodium ion interacting with the inner ring of Glu17 residues in CC-Type2-LL-L17E (blue) and CC-Type2-IL-Sg-L17E (green) between pH 3 and 10.5 - A sodium ion is considered as interacting inside the ring of Glu17 residues if it is at a distance of less than 12 Å from each Glu17 residues. From pH ~7 and above the occupancy diminish mainly because of the increased sampling of opened and dissociated barrels, but also partly because the metrics is sensitive to conformations for which more than one side chains of Glu17 point toward the outside of the structure.

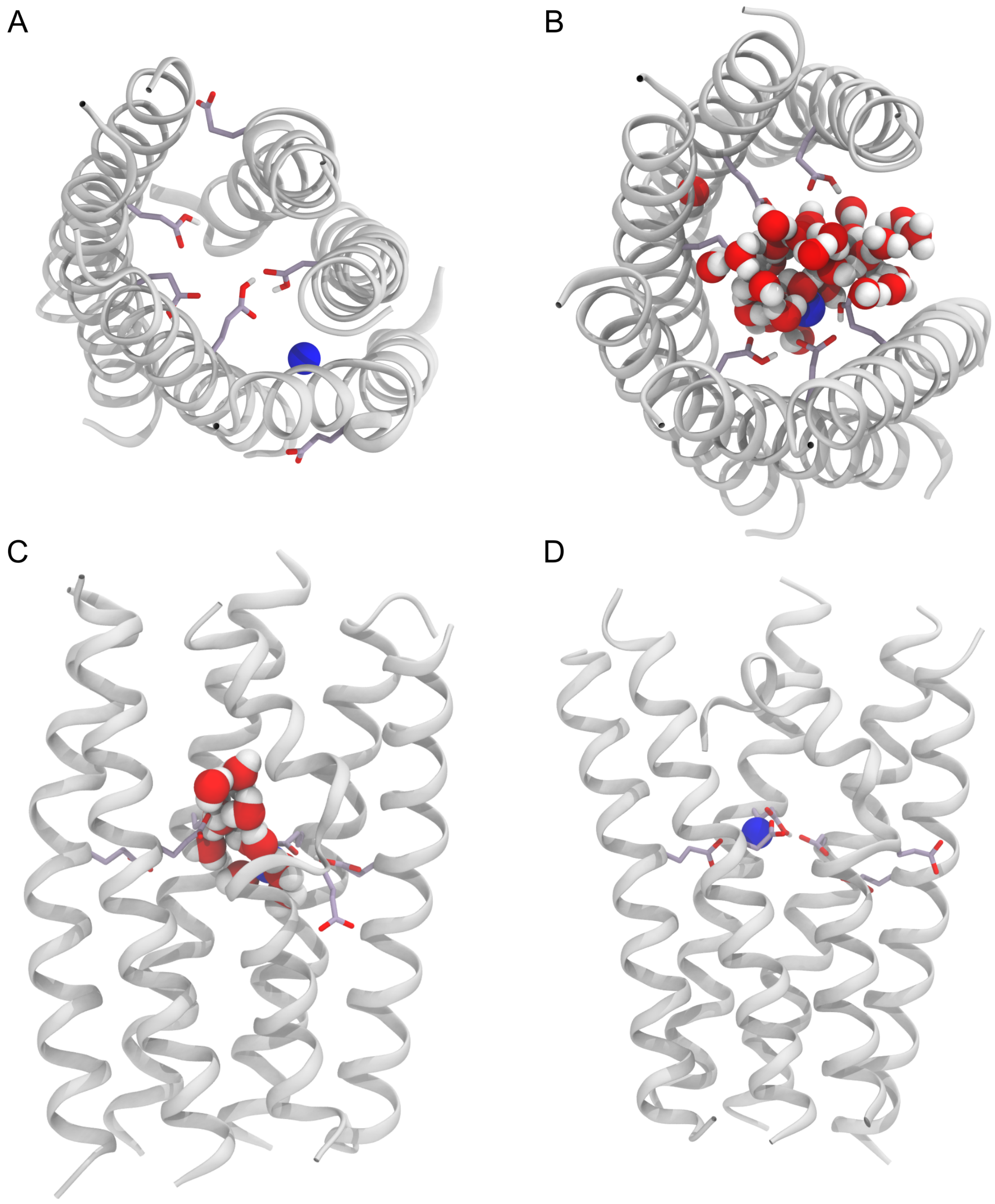

Supplementary Figure 96 Example of CC-Type2-LL-L17E conformations sampled during pH-REMD simulations that deviate significantly from the crystal structure - (A) Top view showing two Glu17 side chains pointing toward the outside of the structure. (B) Top view of the opening of the protein fold. (C & D) Large secondary structure deformation and kink subsequent to a Glu17 residue pointing toward the outside of the structure (side view). The structure is represented with light grey ribbons, Glu17 are shown as purple sticks, water molecules and sodium ions are shown as Van der Waals' spheres with sodium ions coloured in blue.

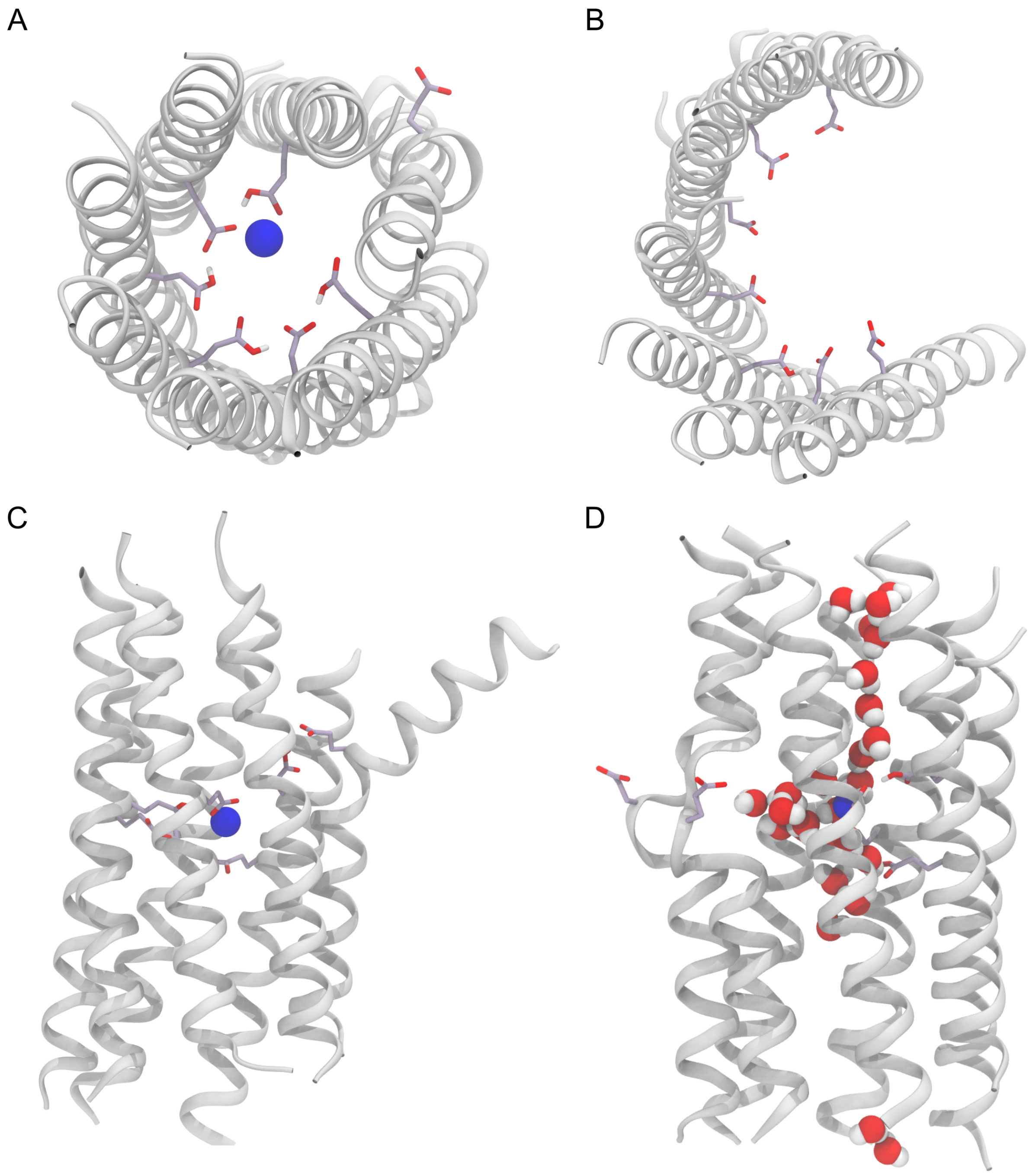

Supplementary Figure 97 Example of CC-Type2-IL-Sg-L17E conformations sampled during pH-REMD simulations that deviate significantly from the crystal structure - (A) Top view showing a Glu17 side chain pointing toward the outside of the structure. (B) Opening of the barrel. (C) Helical kink. (D) Large secondary structure deformation subsequent to a Glu17 residue pointing toward the outside of the structure. The structure is represented with light grey ribbons, Glu17 are shown as purple sticks, water molecules and sodium ions are shown as Van der Waals' spheres with sodium ions coloured in blue.

Sequence threading

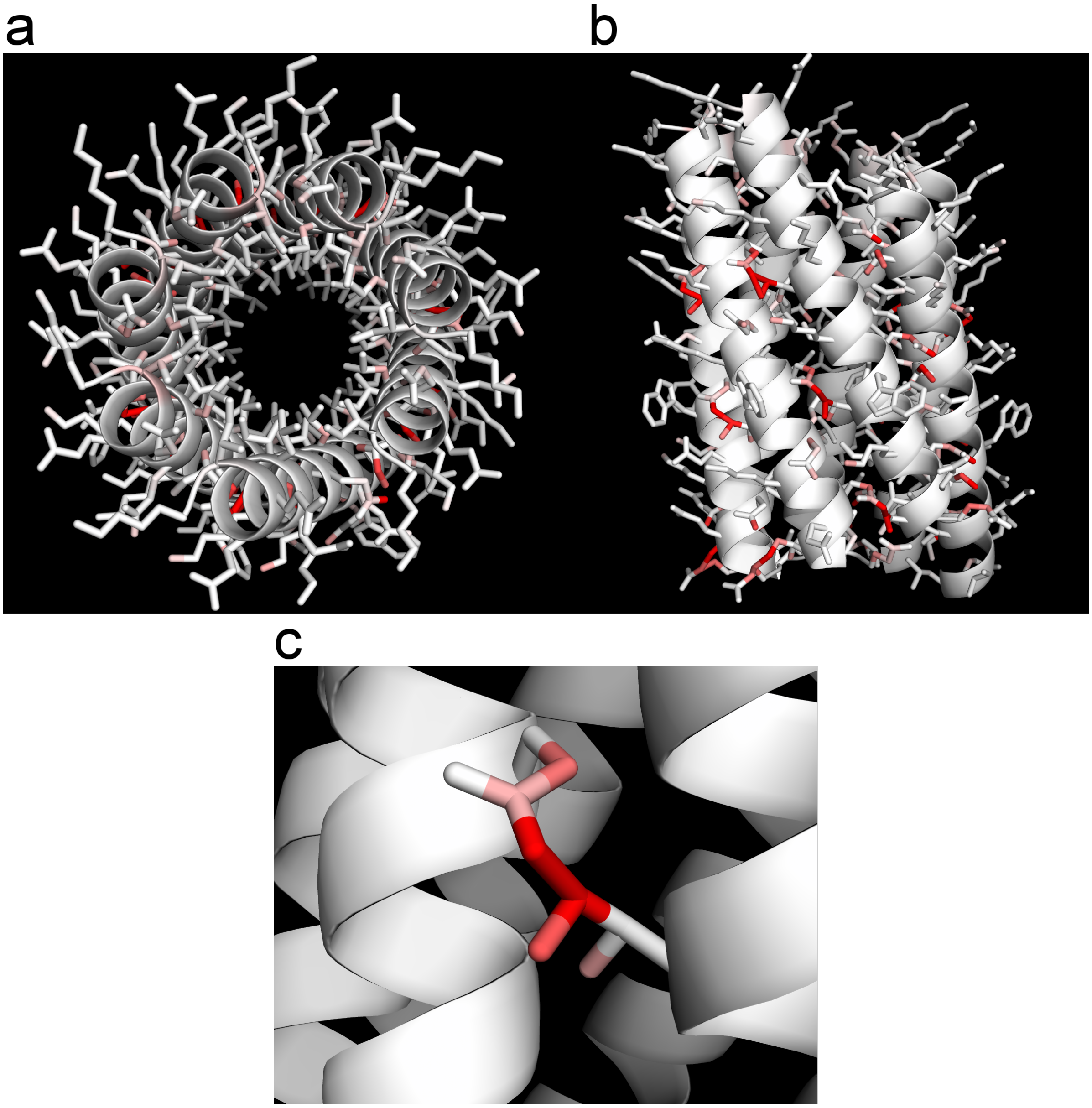

Supplementary Figure 98 Visual representation of clash scores per atom - Viewed from (a) the N termini and (b) the side is the sequence of CC-Pent threaded onto GCN4-pAA highlighting clashes at the peripheral ***e*** and ***g*** positions. (c) An image focused on a pair of clashing (left) Glu and (right) Ile residues at ***e*** and ***g*** positions, respectively. A colour spectrum is used to represent the clash scores for each atom. No clashes are coloured white and minimal to extensive clashes are coloured from pink to red respectively. At very short distance, atoms that are not covalently bound are rendered as being covalently bound.

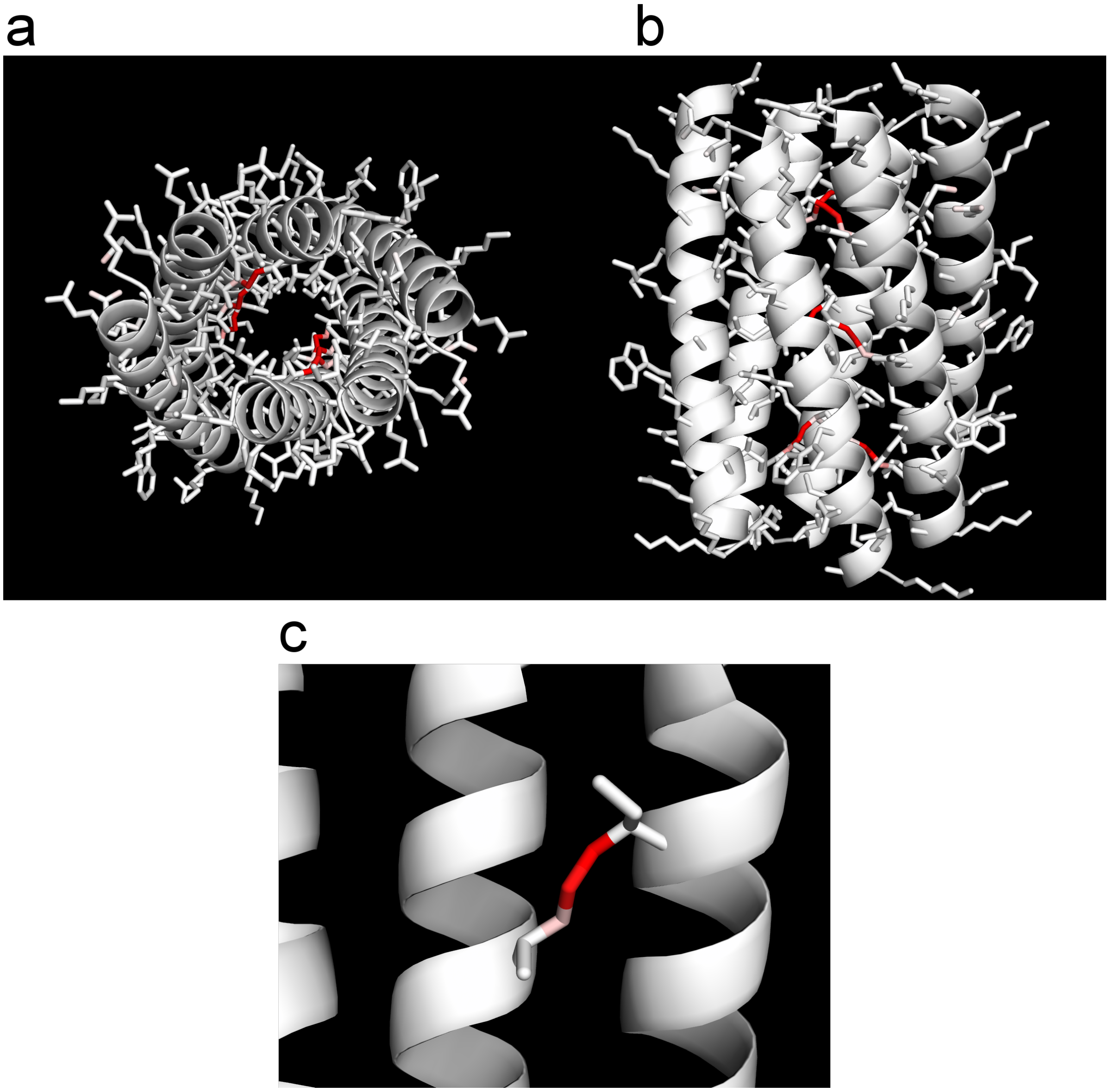

Supplementary Figure 99 Visual representation of clash scores per atom - Viewed from (a) the N termini and (b) the side is the sequence of CC-Type2-II threaded onto CC-Type2-LL-Sg highlighting clashes at the internal ***a*** and ***d*** positions. (c) An image focused on a pair of clashing Ile residues at ***a*** and ***d*** positions. A colour spectrum is used to represent the clash scores for each atom. No clashes are coloured white and minimal to extensive clashes are coloured from pink to red respectively. At very short distance, atoms that are not covalently bound are rendered as being covalently bound.

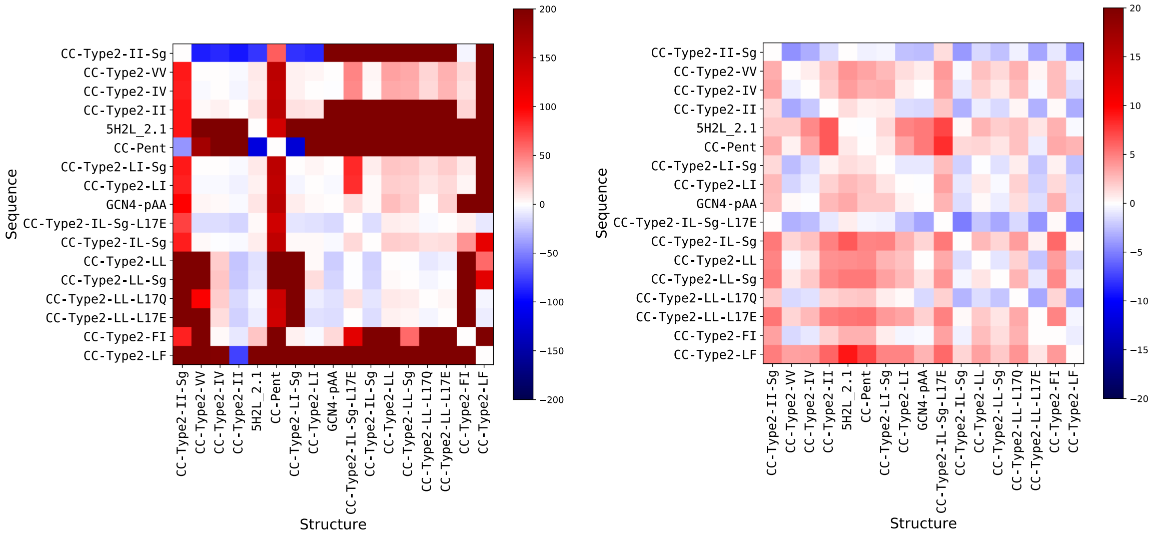

Supplementary Figure 100 Rescoring of the sequence-threading matrix with alternative force fields. Total energy from scoring with (left) amber99-SB-ILDN forcefield[11] with amber99 OBC implicit solvent (a physical-based force field) and (right) DFIRE2.0[12] (a statistical-based force field). Scores are normalised to the diagonal line and are divided by the number of chains in the assembly.

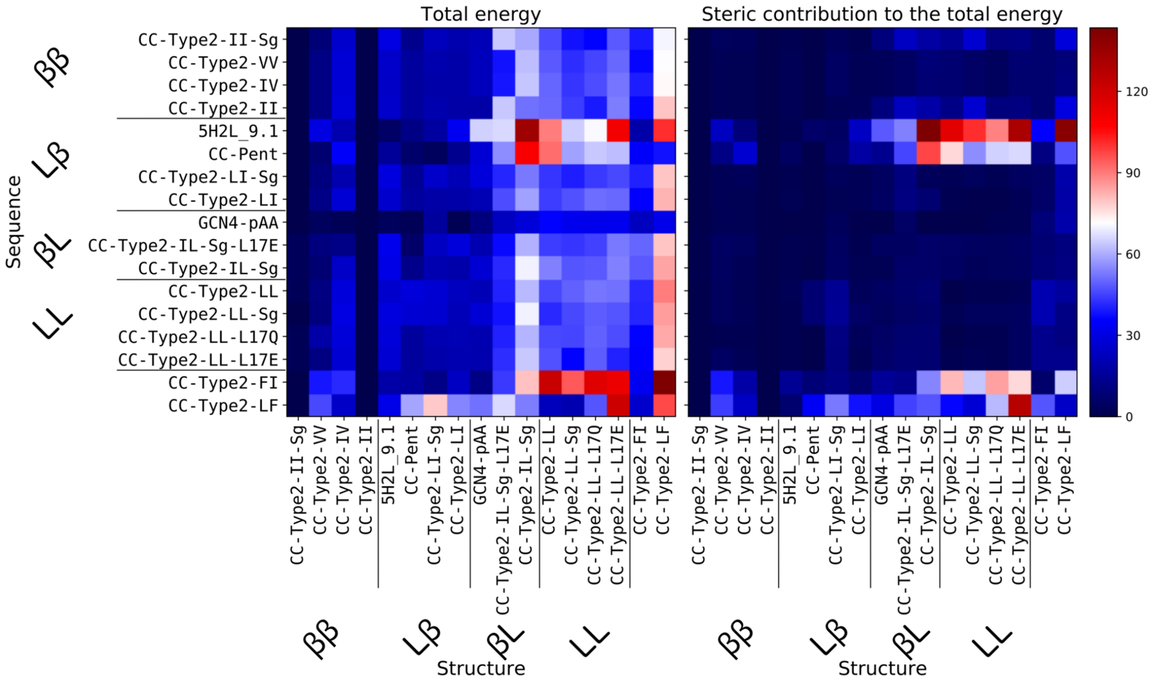

Supplementary Figure 101 Standard deviation of the chain scores for each sequence threading - The standard deviation of chain BUDE scores from modelling sequences discussed in the manuscript onto all parallel structures discussed in the manuscript. Colormaps show the (left) standard deviation in BUDE points (a pseudo-energy function) and extracted from this (right) the standard deviation of the steric component of the BUDE score.

Supplementary References

[1] Thomson, A.R., Wood, C.W., Burton, A.J., Bartlett, G.J., Sessions, R.B. et al. Computational design of water-soluble α-helical barrels *Science* **346**, 485–488 (2014).

[2] Blomberg, R., Kries, H., Pinkas, D.M., Mittl, P.R.E., Grütter, M.G. et al. Precision is essential for efficient catalysis in an evolved Kemp eliminase. *Nature* **503**, 418–421 (2013).

[3] Case, D., Betz, R., Botello-Smith, W., Cerutti, D., Cheatham, T. et al. AMBER 2016 (2016).

[4] Maier, J.A., Martinez, C., Kasavajhala, K., Wickstrom, L., Hauser, K.E. et al. ff14SB: Improving the Accuracy of Protein Side Chain and Backbone Parameters from ff99SB *J. Chem. Theory Comput.* **11**, 3696–3713 (2015).

[5] Mongan, J., Case, D.A. & McCammon, J.A. Constant pH molecular dynamics in generalized Born implicit solvent *J. Comput. Chem.* **25**, 2038–2048 (2004).

[6] Swails, J.M., York, D.M. & Roitberg, A.E. Constant pH replica exchange molecular dynamics in explicit solvent using discrete protonation states: Implementation, testing, and validation *J. Chem. Theory Comput.* **10**, 1341–1352 (2014).

[7] Swails, J.M. & Roitberg, A.E. Enhancing conformation and protonation state sampling of hen egg white lysozyme using pH replica exchange molecular dynamics *J. Chem. Theory Comput.* **8**, 4393–4404 (2012).

[8] Roe, D.R. & Cheatham, T.E. PTRAJ and CPPTRAJ: Software for processing and analysis of molecular dynamics trajectory data *J. Chem. Theory Comput.* **9**, 3084–3095 (2013).

[9] Smart, O.S., Neduvelil, J.G., Wang, X., Wallace, B.A. & Sansom, M.S.P. HOLE: A program for the analysis of the pore dimensions of ion channel structural models *J. Mol. Graph.* **14**, 354–360 (1996).

[10] Michaud-Agrawal, N., Denning, E.J., Woolf, T.B. & Beckstein, O. MDAnalysis: A toolkit for the analysis of molecular dynamics simulations *J. Comput. Chem.* **32**, 2319–2327 (2011).

[11] Lindorff-Larsen, K., Piana, S., Palmo, K., Maragakis, P., Klepeis, J.L. et al. Improved side-chain torsion potentials for the Amber ff99SB protein force field *Proteins Struct. Funct. Bioinforma.* **78**, 1950–1958 (2010).

[12] Yang, Y. & Zhou, Y. Ab initio folding of terminal segments with secondary structures reveals the fine difference between two closely related all-atom statistical energy functions *Protein Sci.* **17**, 1212–1219 (2008).
