# Supplementary material for: Maintaining and breaking symmetry in homomeric coiled-coil assemblies
Source: Nat Commun. 2018 Oct 8;9:4132. doi: 10.1038/s41467-018-06391-y (PMC6175849; doi:10.1038/s41467-018-06391-y)
Supplement: Supplementary file 3 — Description of Additional Supplementary Files [file 41467_2018_6391_MOESM3_ESM.pdf]

Description of Additional Supplementary Files

File Name: Supplementary Movie 1
Description: Trajectory of CC-Type2-IL-Sg-L17E at pH 4.0 obtained from the pHREMD simulations – Top view from the C-terminal end with water molecules removed (left) and side view (right) of the protein fold. The structure is represented with light grey ribbons, Glu17 are shown as purple sticks, water molecules and sodium ions are shown as van der Waals' spheres with sodium ions coloured in blue.

File Name: Supplementary Movie 2
Description: Trajectory of CC-Type2-LL-L17E at pH 4.0 obtained from the pHREMD simulations –Top view from the C-terminal end (left) and side view (right) of the protein fold. The structure is represented with light grey ribbons, Glu17 are shown as purple sticks, water molecules and sodium ions are shown as Van der Waals' spheres with sodium ions coloured in blue.
